# Radical cation Diels–Alder reactions of arylidene cycloalkanes

**DOI:** 10.3762/bjoc.18.112

**Published:** 2022-08-25

**Authors:** Kaii Nakayama, Hidehiro Kamiya, Yohei Okada

**Affiliations:** 1 Department of Chemical Engineering, Tokyo University of Agriculture and Technology, 2-24-16 Naka-cho, Koganei, Tokyo 184-8588, Japan; 2 Department of Applied Biological Science, Tokyo University of Agriculture and Technology, 3-5-8 Saiwai-cho, Fuchu, Tokyo 183-8509, Japan

**Keywords:** arylidene cycloalkane, Diels–Alder reaction, radical cation, single-electron transfer, spiro ring system

## Abstract

TiO_2_ photoelectrochemical and electrochemical radical cation Diels–Alder reactions of arylidene cycloalkanes are described, leading to the construction of spiro ring systems. Although the mechanism remains an open question, arylidene cyclobutanes are found to be much more effective in the reaction than other cycloalkanes. Since the reaction is completed with a substoichiometric amount of electricity, a radical cation chain pathway is likely to be involved.

## Introduction

**Figure 1:**
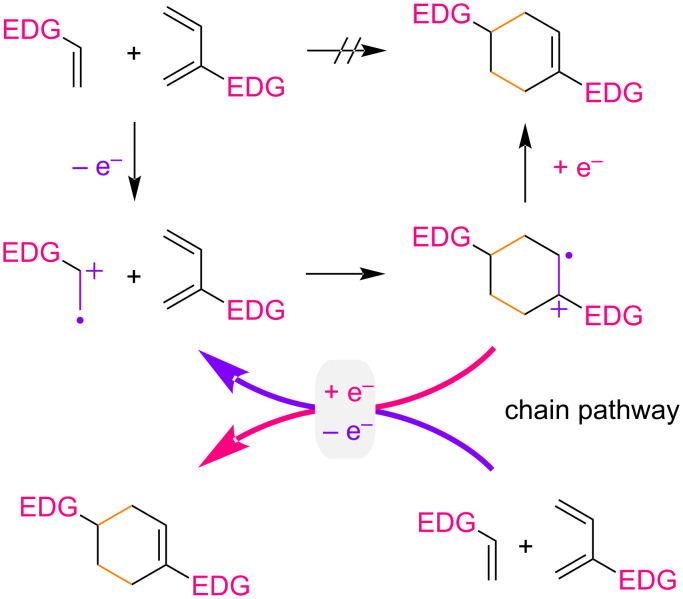
Plausible mechanism of the radical cation Diels–Alder reaction (EDG: electron-donating group).

**Figure 2:**
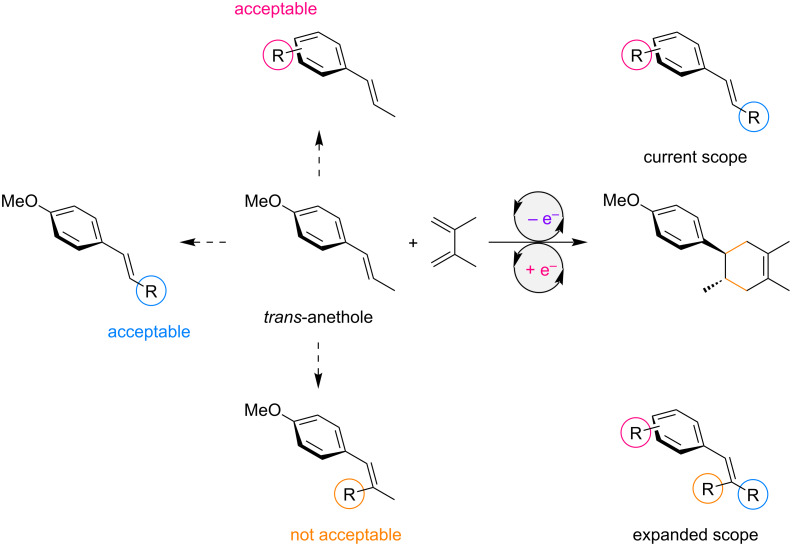
Landscape of the radical cation Diels–Alder reaction.

Single-electron transfer is one of the simplest modes for small molecule activation, employing a polarity inversion to generate radical ions which have proven to be unique reactive intermediates in the field of synthetic organic chemistry. A radical cation Diels–Alder reaction is a typical example of this activation mode since both the original diene and dienophile are electron-rich and thus not an effective combination of reactants [1–11]. Single-electron transfer makes the construction of six-membered ring systems possible. In general, single-electron oxidation of an electron-rich dienophile generates its radical cation which is then trapped by the diene (Figure 1). Since the forming cyclohexene remains in the radical cation state as well, one electron reduction is required to complete the net redox neutral transformation. Therefore, a chain pathway can be involved, where an electron acts as a catalyst rather than a reagent [12–18]. In this reaction format, *trans*-anethole is an electron-rich dienophile and has widely been studied as a benchmark for single-electron transfer using photochemical and electrochemical methods [19–32]. A one electron oxidant can also be an initiator for this transformation [33–35]. Overall, the scope of the reaction has been expanding. Starting from *trans*-anethole, several functionalities at the β-position are found to be compatible with the reaction, while those at the aryl ring are somewhat limited (Figure 2). It should be noted that a second substituent at the β-position of *trans*-anethole has a significant impact on the reaction and surprisingly, even an additional methyl group is not acceptable.

**Scheme 1:**
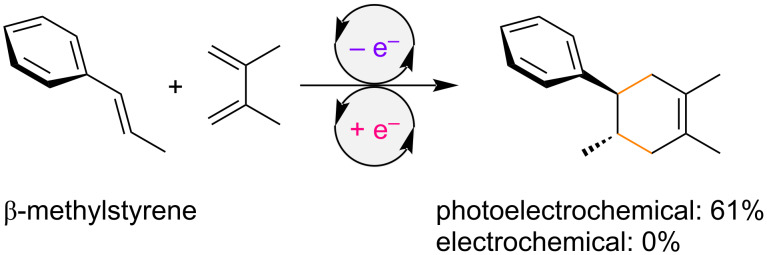
Radical cation Diels–Alder reaction of β-methylstyrene.

We have developed radical cation cycloadditions using (photo)electrochemical single-electron transfer in lithium perchlorate (LiClO_4_)/nitromethane (CH_3_NO_2_) solution [36–44]. During the course of our studies, we found that the TiO_2_ photoelectrochemical approach was more beneficial than simple electrochemistry in most cases, probably because both single-electron oxidation and reduction are made possible at the same surface [45]. This is especially true for the radical cation Diels–Alder reaction, since non-substituted β-methylstyrene, which was previously reported as an unsuccessful dienophile, was found to participate under TiO_2_ photoelectrochemical conditions (Scheme 1) [46–47]. We questioned whether the scope of the radical cation Diels–Alder reaction could be further expanded, with particular interest on the installation of a second substituent at the β-position. Described herein is our unexpected finding that various spiro ring systems can be constructed by a radical cation Diels–Alder reaction of arylidene cycloalkanes.

## Results and Discussion

**Scheme 2:**
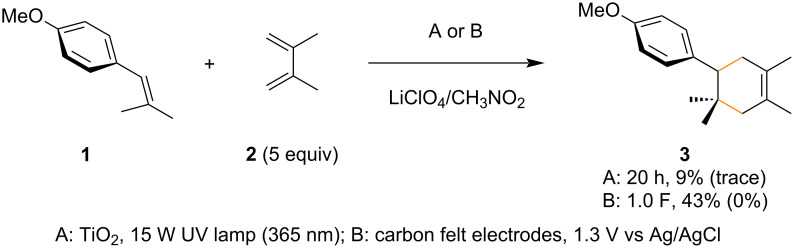
Radical cation Diels–Alder reaction of β-methylanethole (**1**). Recovered starting material is reported in parentheses.

**Figure 3:**
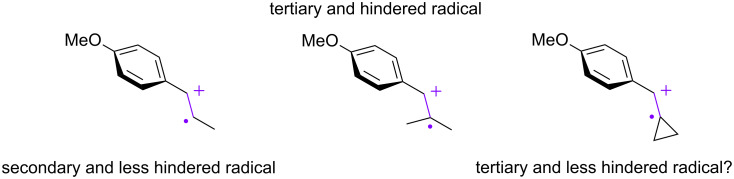
Formal expression of radical cations.

**Scheme 3:**
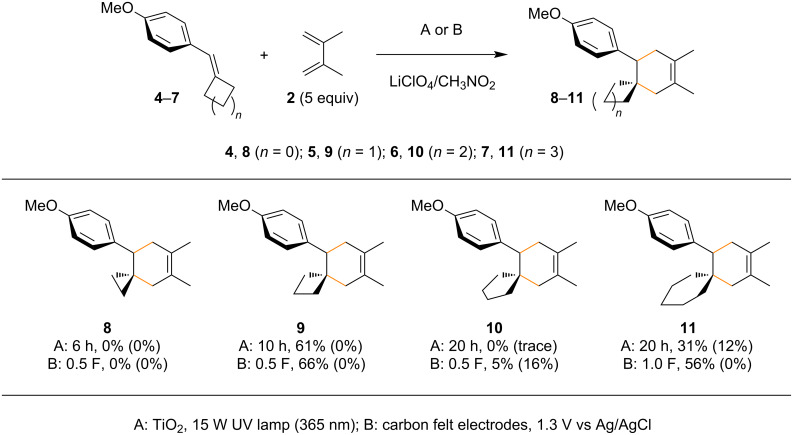
Radical cation Diels–Alder reactions of the arylidene cycloalkanes (**4**–**7**). Recovered starting material is reported in parentheses.

The present work began with the reaction of β-methylanethole (**1**) with 2,3-dimethyl-1,3-butadiene (**2**) under TiO_2_ photoelectrochemical and electrochemical conditions (Scheme 2). The initial attempts using both conditions provided us two small indications that the reaction was not totally inaccessible. The simple electrochemical approach gave a better result than TiO_2_ photoelectrochemistry. Furthermore, we confirmed that the additional methyl group at the β-position had a significant impact on the reaction. In general, tertiary radicals (or cations) are more stable than secondary ones and therefore, the additional methyl group seems to have a strong steric effect (Figure 3). If so, tying up the two methyl groups as a cyclopropane ring may decrease the steric hindrance at the β-position and improve the reaction. Unfortunately, the arylidene cyclopropane **4** was found to be totally unreactive under both conditions (Scheme 3). However, to our surprise, the arylidene cyclobutane **5** was found to be productive and the corresponding spiro ring compound **9** was obtained in good yield. The arylidene cyclopentane **6** and cyclohexane **7** were found to be less effective for the reaction and in particular, the former (**6**) was almost totally unsuccessful. Although the mechanism remains unclear, Knowles and Romanov-Michailidis recently reported that a similar trend is observed in photosensitized [2 + 2] cycloadditions [48]. Since their report was not a [4 + 2] but a [2 + 2] reaction and they proposed an energy transfer mechanism as opposed to an electron transfer pathway, it cannot be directly compared to our results. Even so, it would be fair to say that there is some correlation between these arylidene cycloalkane cycloadditions.

Control studies are summarized in Table 1. LiClO_4_, TiO_2_, and light were crucial for the reaction (Table 1, entries 1–4) and the equivalents of the diene **2** was also key (entries 5 and 6 in Table 1). The reaction was sensitive toward atmosphere; both oxygen and argon had a negative impact (Table 1, entries 7 and 8). In the electrochemical approach, potentiostatic conditions gave better results than galvanostatic conditions and more importantly, it was found that the reaction was completed within 0.5 F/mol (Table 1, entries 9–11). This result clearly suggests that a chain pathway is involved in the reaction.

**Table 1:** Control studies for the radical cation Diels–Alder reaction of the arylidene cyclobutane **5**.

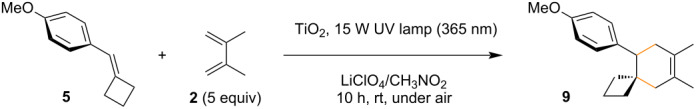		

entry	deviation from the standard conditions	yield (%)^a^

1		61 (0)
2	no LiClO_4_	0 (40)
3	no light	0 (83)
4	no TiO_2_	15 (32)
5	2 equiv of diene	22 (0)
6	10 equiv of diene	58 (0)
7	under O_2_	19 (16)
8	under Ar	21 (0)
9	1.3 V vs. Ag/AgCl, 0.1 F/mol	22 (52)
10	1.3 V vs. Ag/AgCl, 0.5 F/mol	66 (0)
11	1.0 mA, 0.5 F/mol	46 (trace)

^a^recovered starting material is reported in parentheses.

**Scheme 4:**
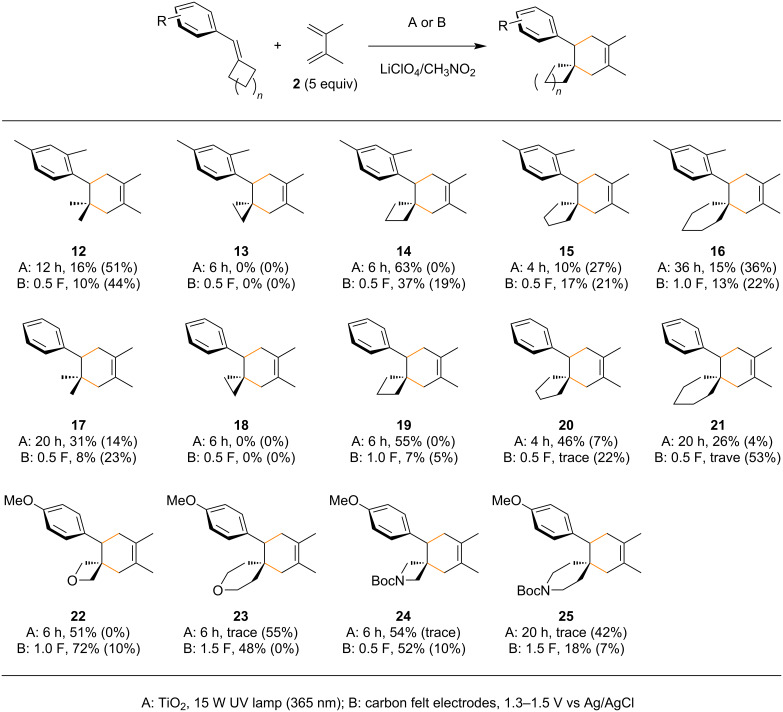
Scope of the radical cation Diels–Alder reaction of arylidene cycloalkanes (recovered starting material is reported in parentheses).

The scope of the reaction was studied using dimethyl and non-substituted aryl rings in combination with several cycloalkanes (Scheme 4). The TiO_2_ photoelectrochemical approach was more beneficial than simple electrochemistry in many cases for these dienophiles, which accords well with our previous reports. The ring size effect of cycloalkanes was also clearly observed and cyclobutane was much more effective than the others. A similar trend was observed using some heterocycles, which also accorded well with the previous report by Knowles and Romanov-Michailidis.

## Conclusion

In conclusion, we have demonstrated that radical cation Diels–Alder reactions of arylidene cycloalkanes are enabled under TiO_2_ photoelectrochemical and electrochemical conditions to construct various spiro ring systems. Although further detailed experimental and/or theoretical studies are required to elucidate the complete mechanistic picture, arylidene cyclobutanes were found to be much more effective than others. A similar ring size effect was observed by Knowles and Romanov-Michailidis in photosensitized [2 + 2] cycloadditions of benzylidene cycloalkanes and therefore, the results described herein may support a detailed mechanistic understanding. Further experimental and theoretical studies of radical cation cycloadditions of arylidene cycloalkanes are under investigation in our laboratory.

## Experimental

**Photoelectrochemical:** The appropriate arylidene cycloalkane (0.20 mmol), 2,3-dimethyl-1,3-butadiene (**2**, 113 μL, 1.0 mmol), and TiO_2_ (100 mg) were added to a solution of LiClO_4_/CH_3_NO_2_ (1.0 M, 4.0 mL) while stirring at room temperature. The resulting reaction mixture was stirred at room temperature in front of a 15 W UV lamp (365 nm). Then, the solution was diluted with water and extracted with EtOAc. The combined organic layers were dried over Na_2_SO_4_, filtered, and concentrated in vacuo. Yields were determined by ^1^H NMR analysis using dibromomethane as an internal standard. Silica gel column chromatography (hexane/ethyl acetate) gave the corresponding spiro ring compound.

**Electrochemical:** The appropriate arylidene cycloalkane (0.20 mmol) and 2,3-dimethyl-1,3-butadiene (**2**, 113 μL, 1.0 mmol) were added to a solution of LiClO_4_/CH_3_NO_2_ (1.0 M, 4.0 mL) while stirring at room temperature. The resulting reaction mixture was electrolyzed at 1.3–1.5 V vs Ag/AgCl using carbon felt electrodes (10 mm × 10 mm) in an undivided cell with stirring. Then, the solution was diluted with water and extracted with EtOAc. The combined organic layers were dried over Na_2_SO_4_, filtered, and concentrated in vacuo. Yields were determined by ^1^H NMR analysis using dibromomethane as an internal standard. Silica gel column chromatography (hexane/ethyl acetate) gave the corresponding spiro ring compound.

## Supporting Information

File 1 General remarks, photocatalyst analyzation data, synthesis procedure, additional control studies, electrochemical measurements, and characterization data, including copies of ^1^H and ^13^C NMR spectra.
