# Supplementary material for: Radical cation Diels–Alder reactions of arylidene cycloalkanes
Source: Beilstein J Org Chem. 2022 Aug 25;18:1100–6. doi: 10.3762/bjoc.18.112 (PMC9443414; doi:10.3762/bjoc.18.112)
Supplement: File 1 — General remarks, photocatalyst analyzation data, synthesis procedure, additional control studies, electrochemical measurements, and characterization data, including copies of 1H and 13C NMR spectra. [file Beilstein_J_Org_Chem-18-1100-s001.pdf]

BEILSTEIN JOURNAL OF ORGANIC CHEMISTRY

Supporting Information

for

Radical cation Diels–Alder reactions of arylidene cycloalkanes

Kaii Nakayama, Hidehiro Kamiya and Yohei Okada

Beilstein J. Org. Chem. **2022,** *18,* 1100–1106. doi:10.3762/bjoc.18.112

General remarks, photocatalyst analyzation data, synthesis procedure, additional control studies, electrochemical measurements, and characterization data, including copies of ^{1}H and ^{13}C NMR spectra

License and Terms: This is a supporting information file under the terms of the Creative Commons Attribution License (https://creativecommons.org/licenses/by/4.0). Please note that the reuse, redistribution and reproduction in particular requires that the author(s) and source are credited and that individual graphics may be subject to special legal provisions.
The license is subject to the *Beilstein Journal of Organic Chemistry* terms and conditions: (https://www.beilstein-journals.org/bjoc/terms)

Table of contents

1. General remarks S1

2. TiO_2 analysis.. S2

3. Synthetic procedure S3

4. Additional optimization of the reaction conditions.. S9

5. Electrochemical measurements.. S10

6. Characterization data, and copies of ^{1}H and ^{13}C NMR spectra.. S21

1. General remarks

All reagents and solvents were purchased from commercial sources and used without further purification. TiO_2 used in this work was AEROXIDE® TiO2 P 25. Reactions were monitored by thin layer chromatography (TLC) carried out on silica gel plates, with detection by UV absorption (254 nm) and by heating the plates after dipping them in a solution of 12 M molybdo(VI) phosphoric acid *n*-hydrate in 95% ethanol. Silica gel (particle size 40–50 µm, normal or reversed phase) was used for column chromatography. ^{1}H NMR spectra were collected on a 500 or 300 MHz NMR spectrometer using the deuterated solvent as an internal deuterium reference. Chemical shift data are given in δ units calibrated with residual protic solvent. The multiplicity of a signal is indicated as follows: s, singlet; d, doublet; t, triplet; q, quartet; quint, quintet; m, multiplet. ^{13}C NMR spectra were collected at 125 MHz with proton decoupling using the deuterated solvent as an internal carbon reference. Chemical shift data are given in δ units calibrated with residual solvent. High-resolution mass spectra (HRMS) were collected by electrospray ionization (ESI)- or direct analysis in real time (DART)-time-of-flight (TOF) spectrometers.

2. TiO_2 analysis

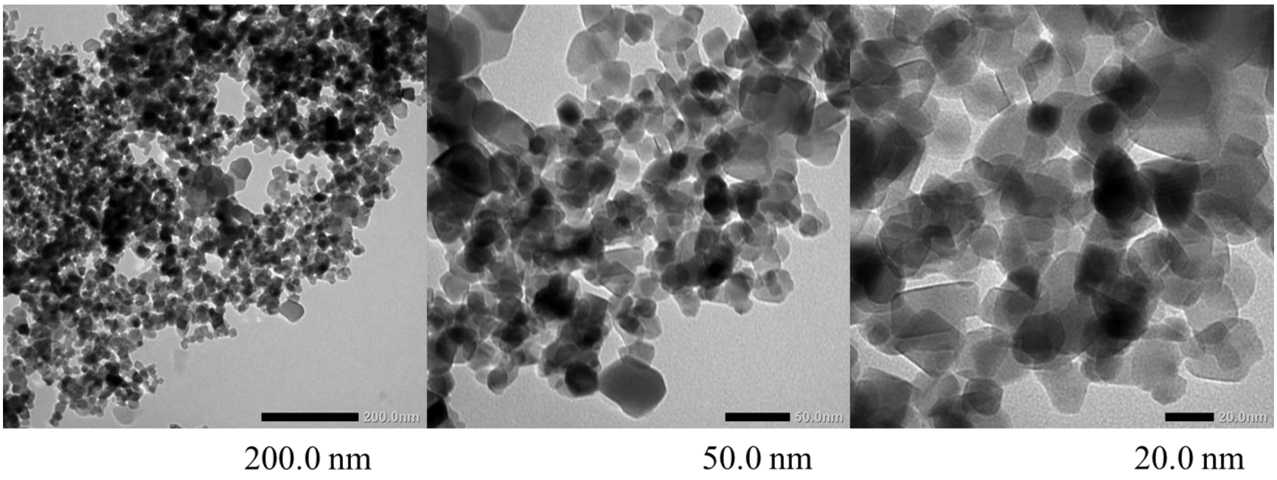

Figure S1. TEM images of TiO_2 used in this work.

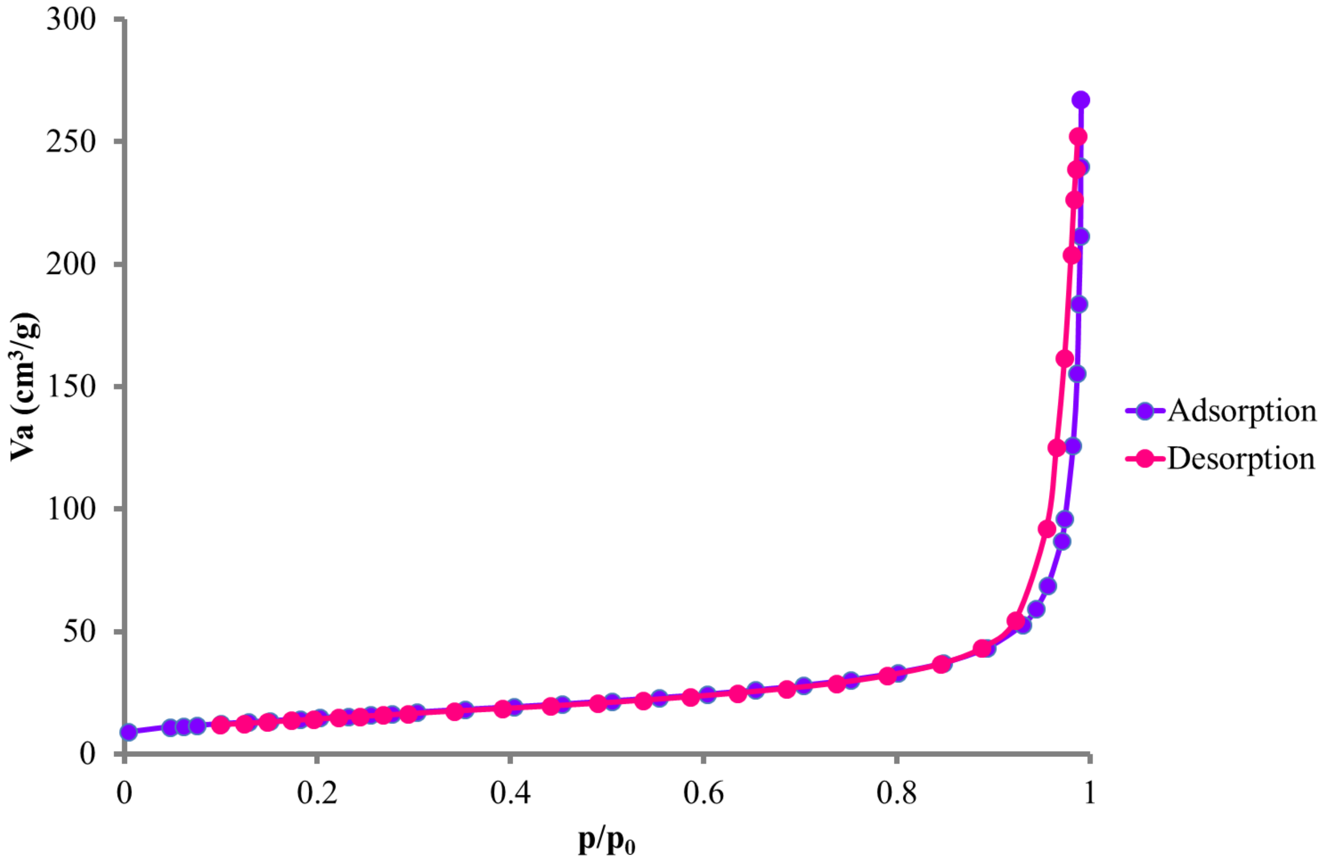

Figure S2. Adsorption isotherm of TiO_2 used in this work.

3. Synthetic procedure

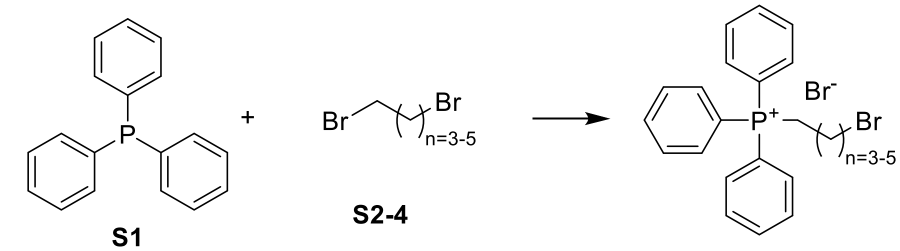

S1 S2-4 S5-7

*General procedure for the synthesis of Wittig reagents (**S5-7**).*[1] The mixture of triphenylphosphine (**S1**, 1.3 g, 5 mmol) and respective dibromo alkane (**S2–4**, 25 mmol) was stirred at 90 °C for 5.5 h. After that, the resulting reaction mixture was cooled to rt, washed with toluene, and dried *in vacuo*. The mixture was sonicated with diethyl ether for 1 h and filtration gave the title compound quantitatively as a white solid.

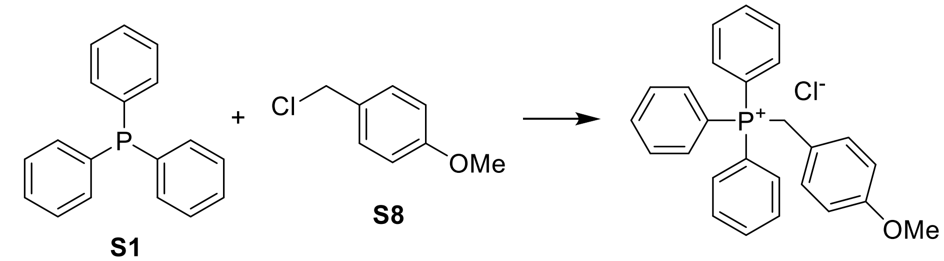

S1 S8 S9

*Synthesis of p-methoxybenzyl triphenyl phosphonium chloride (**S9**).* To a solution of the triphenylphosphine (**S1**, 5.2 g, 20.0 mmol) in toluene (50 mL) stirred at rt was added *p*-methoxybenzyl chloride (**S8**, 1.7 g, 20.0 mmol). The resulting reaction mixture was stirred at 110 °C for 12 h and washed with toluene; filtration gave the title compound in 66% yield (5.5 g, 13.2 mmol) as a white solid.

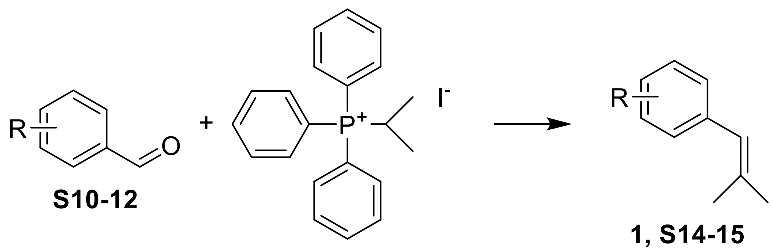

S10-12 S13 1, S14-15

*General procedure for the synthesis of β-dimethyl arylidenes (**1, S14-15**).*[2] To a solution of the Wittig reagent (**S13**, 4.3 g, 10.0 mmol) in THF (50 mL) stirred at rt was added KO*t*-Bu (1.1 g, 10.0 mmol). The resulting reaction mixture was stirred at rt for 30 min, respective aldehyde (**S10-12**, 5.0 mmol) was added, and the reaction mixture was stirred at rt for 12 h. Hydrogen peroxide (2 mL) was added, diluted with water, and extracted with EtOAc. The combined organic layers were dried over sodium sulfate, filtered, and concentrated in vacuo. Silica gel column chromatography (hexane only) gave the titled compound.

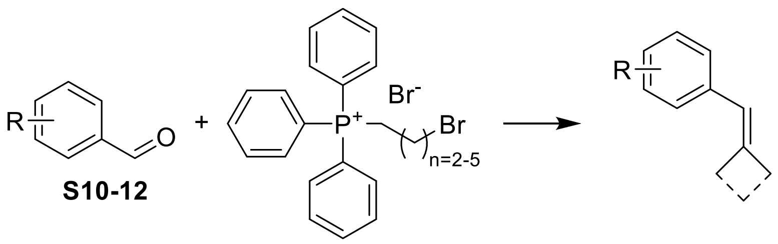

S10-12 S5-7, S16 4, 5, 7, S17-24

General procedure for the synthesis of β-cycloalkyl arylidenes (4, 5, 7, S17-24).[3] To a solution of the Wittig reagent (**S5-7, S16**, 7.5 mmol) in THF (50 mL) stirred at rt was added KO*t*-Bu (1.7 g, 15.0 mmol). The resulting reaction mixture was stirred at 70 °C for 1 h, then the respective aldehyde (**S10-12**, 5.0 mmol) was added, and the reaction mixture was stirred at 70 °C for 3 h. After the reaction mixture was cooled to rt and stirred for 1 h, hydrogen peroxide (2 mL) was added, diluted with water, and extracted with EtOAc. The combined organic layers were dried over sodium sulfate, filtered, and concentrated in vacuo. Silica gel column chromatography (hexane only or hexane/ethyl acetate 20:1) gave the title compound.

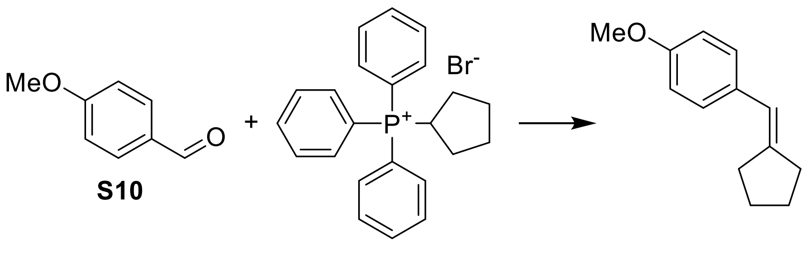

S10 **S25** **6**

Synthesis of p-methoxy-β-cyclopentyl arylidene (6).[4] To a solution of the cyclopentyltriphenylphosphonium bromide (**S25**, 5.0 mmol) in THF (25 mL) stirred at 0 °C was added *n*-butyl lithium (1.6 M in hexanes, 6.3 mmol). The resulting reaction mixture was stirred at 0 °C for 1 h, *p*-anisaldehyde (**S10**, 5.0 mmol) was added, and the reaction mixture was stirred at 0 °C for 16 h. *n*-Pentane was added to precipitate triphenylphosphine oxide, and the mixture was filtered and concentrated in vacuo. Silica gel column chromatography (hexane/ethyl acetate 20:1) gave the title compound.

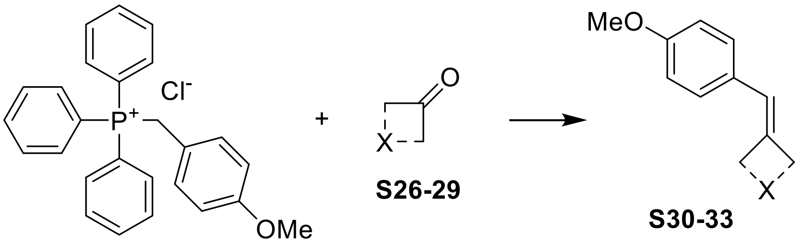

S9 **S26-29** **S30-33**

*General procedure for the synthesis of β-heterocyclic arylidenes (**S30-33**).*[5] To a solution of the Wittig reagent (**S9**, 2.1 g, 5.0 mmol) and KO*t*-Bu (561 mg, 5.0 mmol) in THF (50 mL) stirred at 0 °C was added the respective cyclic ketone (**S26-29**, 6.0 mmol). The resulting reaction mixture was stirred at 0 °C for 2 h, hydrogen peroxide (2 mL) was added, the mixture diluted with water and extracted with EtOAc. The combined organic layers were dried over sodium sulfate, filtered, and concentrated in vacuo. Silica gel column chromatography (hexane/ethyl acetate 10:1) gave the title compound.

(1) K. J. Deane; R. L. Summers; A. M. Lehane; R. E. Martin; R. A. Barrow, *ACS Med. Chem. Lett.*, **2014**, *5*, 576–581.

(2) G. Occhialini; V. Palani; A. E. Wendlandt, *J. Am. Chem. Soc.* **2022**, *144*, 145−152.

(3) Z. An; Y. Liu; Y. Sun; R. Yan, *Chem. Asian J.* **2020**, *15*, 3812–3815.

(4) D. Toryn; S. Grebies; D. Mowpriya; N. Maximilian; S. L. Malte; G. Christian; R. J. Bart; G. Frank, *Angew. Chem. Int. Ed.* **2021**, *60*, 8537–8541.

(5) P. R. D. Murray; W. M. M. Bussink; G. H. M. Davies, F. W. Mei; A. H. Antropow; J. T. Edwards; L. A. D'Agostino; J. M. Ellis; L. G. Hamann; F. R.-Michailidis; R. R. Knowles, *J. Am. Chem. Soc.* **2021**, *143*, 4055−4063.

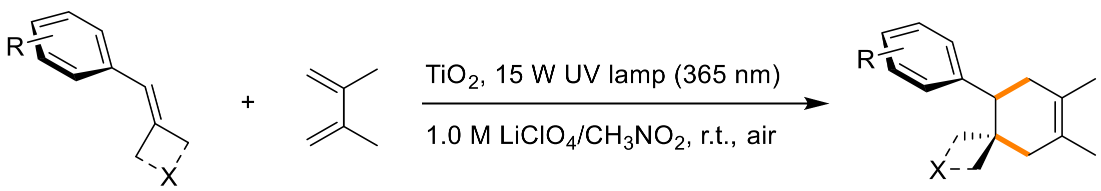

1, 4-7, S14-15
S17-24, S30-33

3, 8-11, 12-25

General procedure for the TiO_2 photocatalytic reactions. To a solution of $LiClO_4$ (1.0 M, 426 mg) in CH_3NO_2 (4 mL) with TiO_2 (100 mg) stirred at rt were added the respective arylidene (0.20 mmol) and 2,3-dimethyl-1,3-butadiene (113 μL, 1.0 mmol). The resulting reaction mixture was irradiated by a 15 W UV lamp (365 nm) at rt under air, diluted with water, and extracted with EtOAc. The combined organic layers were dried over Na_2SO_4, filtered, and concentrated in vacuo. Yields reported in the manuscript were determined by ^{1}H NMR analysis using CH_2Br_2 as an internal standard. Reversed phase silica gel column chromatography (acetonitrile only) and normal phase silica gel column chromatography (hexane only or hexane/ethyl acetate 20:1) gave the target compounds.

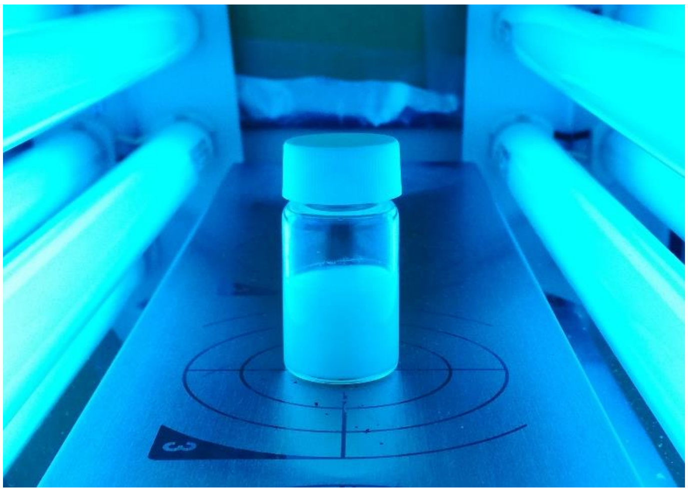

Figure S3. Setup of TiO_2 photocatalytic reactions.

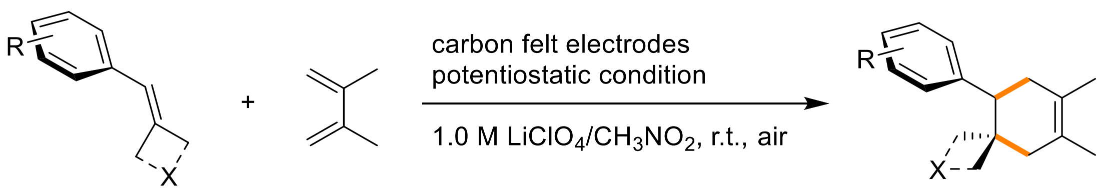

1, 4-7, S14-15
S17-24, S30-33

3, 8-11, 12-25

General procedure for the electrolytic reactions. To a solution of $LiClO_4$ (1.0 M, 426 mg) in CH_3NO_2 (4 mL) stirred at rt were added the respective arylidene (0.20 mmol) and 2,3-dimethyl-1,3-butadiene (113 µL, 1.0 mmol). The resulting reaction mixture was electrolyzed with carbon felt electrodes (1 cm × 1 cm) at rt under air, diluted with water, and extracted with EtOAc. The combined organic layers were dried over Na_2SO_4, filtered, and concentrated in vacuo. Yields reported in the manuscript were determined by ^1H NMR analysis using CH_2Br_2 as an internal standard. Reversed phase silica gel column chromatography (acetonitrile only) and normal phase silica gel column chromatography (hexane only or hexane/ethyl acetate 20:1) gave the target compounds.

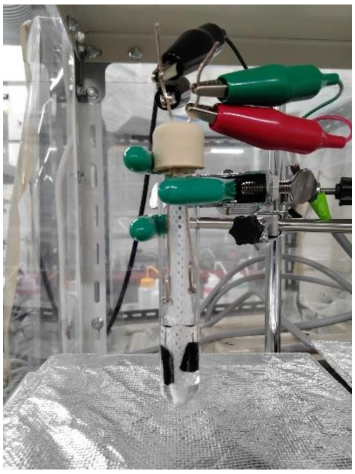

Figure S4. Setup of electrolytic reactions.

4. Additional optimization of the reaction conditions

To a solution of $LiClO_4$ in the solvent (4 mL) with TiO_2 (100 mg) stirred at rt were added the β-cyclobutylstyrene (**S22**, 0.20 mmol, 29.4 μL) and 2,3-dimethyl-1,3-butadiene (**2**). The resulting reaction mixture was irradiated by a 15 W UV lamp (365 nm) or electrolyzed with carbon felt electrodes (1 cm × 1 cm) at rt under air, diluted with water, and extracted with EtOAc. The combined organic layers were dried over Na_2SO_4, filtered, and concentrated in vacuo. Yields reported in the manuscript were determined by ^{1}H NMR analysis using CH_2Br_2 as an internal standard.

Table S1. Optimization of the conditions for radical cation Diels–Alder reaction using arylidene **S22**.

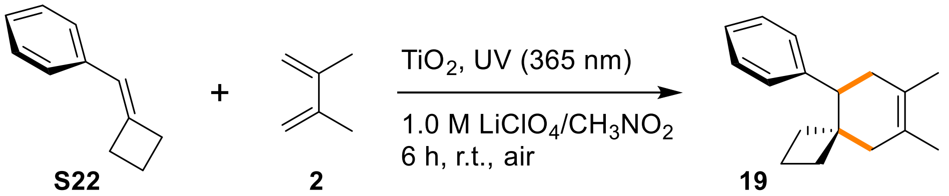

entry	conditions[a]	yield (%)[b]
1		55% (0%)
2	no $LiClO_4$	0% (28%)
3	no light	0% (trace)
4	no TIO_2	0% (26%)
5	2 eq of diene	34% (0%)
6	10 eq of diene	47% (trace)
7	CH_3CN solvent	13% (17%)
8	under O_2	33% (trace)
9	under Ar	9% (16%)
10	reaction for 4 h	50% (6%)
11	CF (+) | CF (-), constant voltage (1.5 V, 0.5 F/mol)	3% (24%)
12	CF (+) | CF (-), constant voltage (1.5 V, 1 F/mol)	7% (5%)

[a]Unless otherwise stated, reactions were carried out on a 0.2 mmol scal of *β*-cyclobutyl-styrene (**S22**) with 5 eq of 2,3-dimethyl-1,3-butadiene (**2**) and 100 mg TiO_2 in 4 ml of CH_3NO_2 using 15 W UV lamp. [b]Determined by ^{1}H NMR analysis using CH_2Br_2 as an internal standard. Recovered starting material is reported in parentheses.

5. Electrochemical measurements

Cyclic voltammograms were recorded using 2 mM of arylidenes in 4 mL of CH_3NO_2. $LiClO_4$ (1.0 M) was used as the supporting electrolyte, a glassy carbon (GC) disk was used as the working electrode, and a Pt wire was used as the counter electrode. All potentials are referenced against the Ag/AgCl redox couple. The scan rate is 50 mV/s.

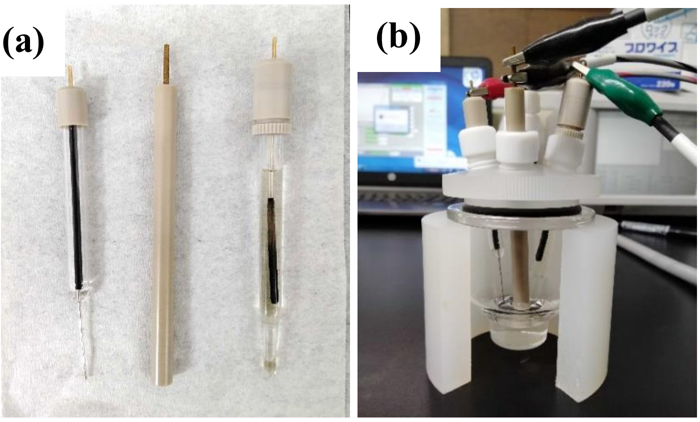

Figure S5. (a) Photograph of used electrodes in this electrochemical measurements, from left: Pt wire, GC disk, and Ag/AgCl electrode, (b) setup of cyclic voltammetry measurements.

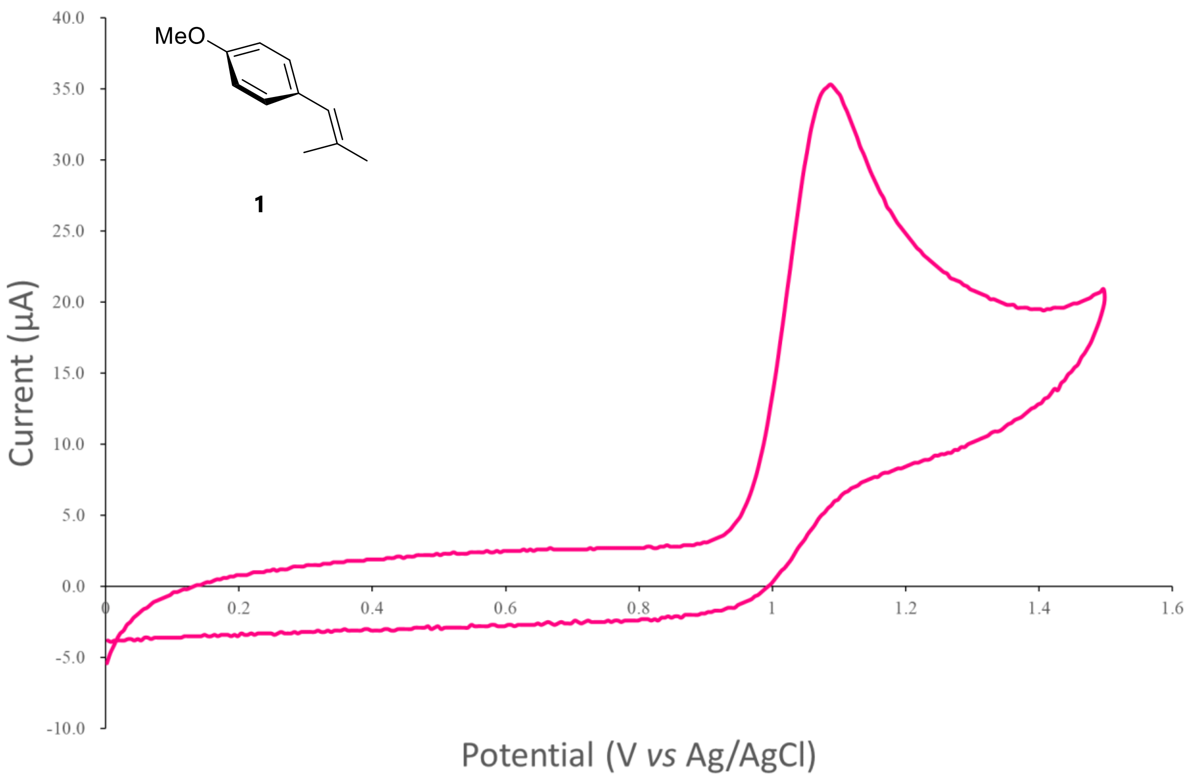

Figure S6. Cyclic voltammogram of the arylidene (**1**).

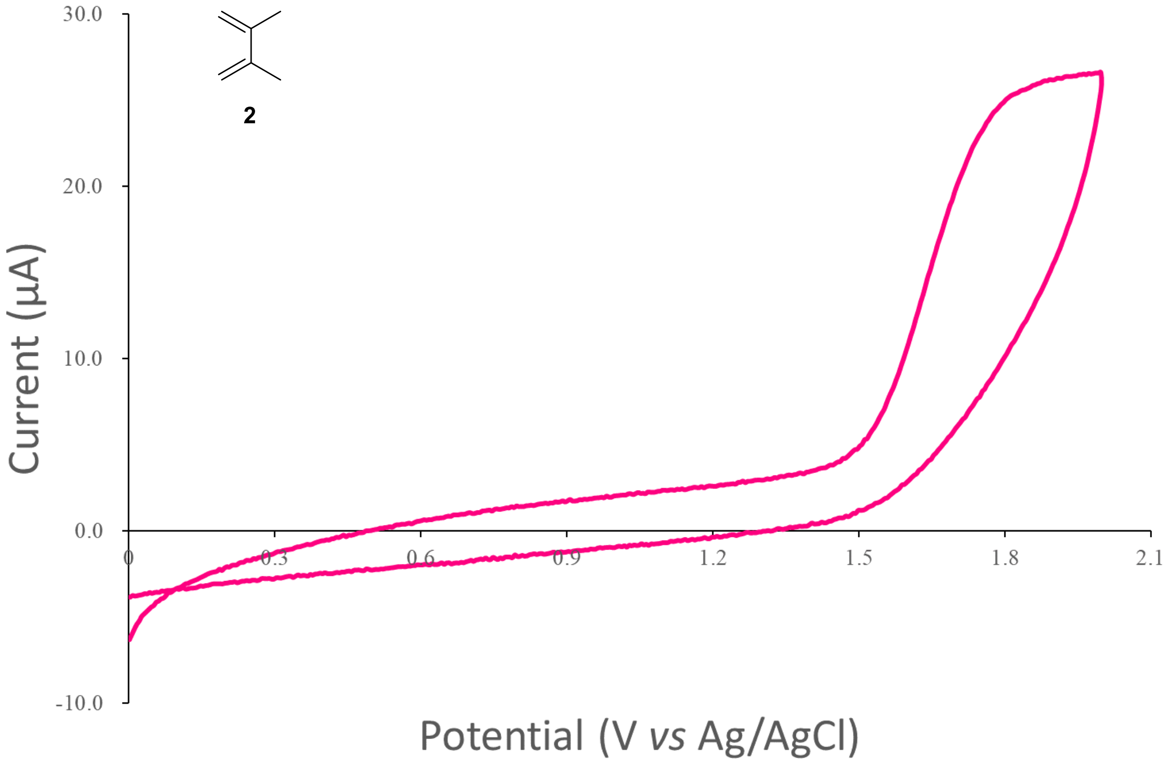

Figure S7. Cyclic voltammogram of the 2,3-dimethyl-1,3-butadiene (**2**).

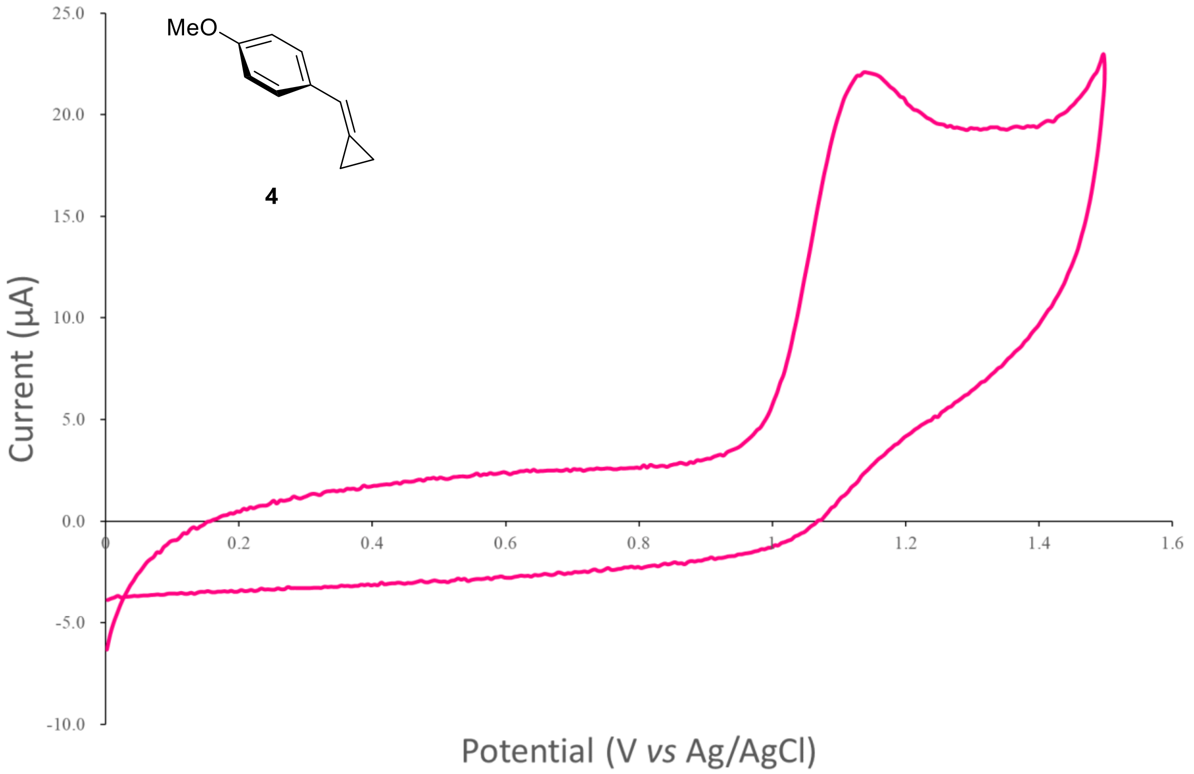

Figure S8. Cyclic voltammogram of the arylidene **4**.

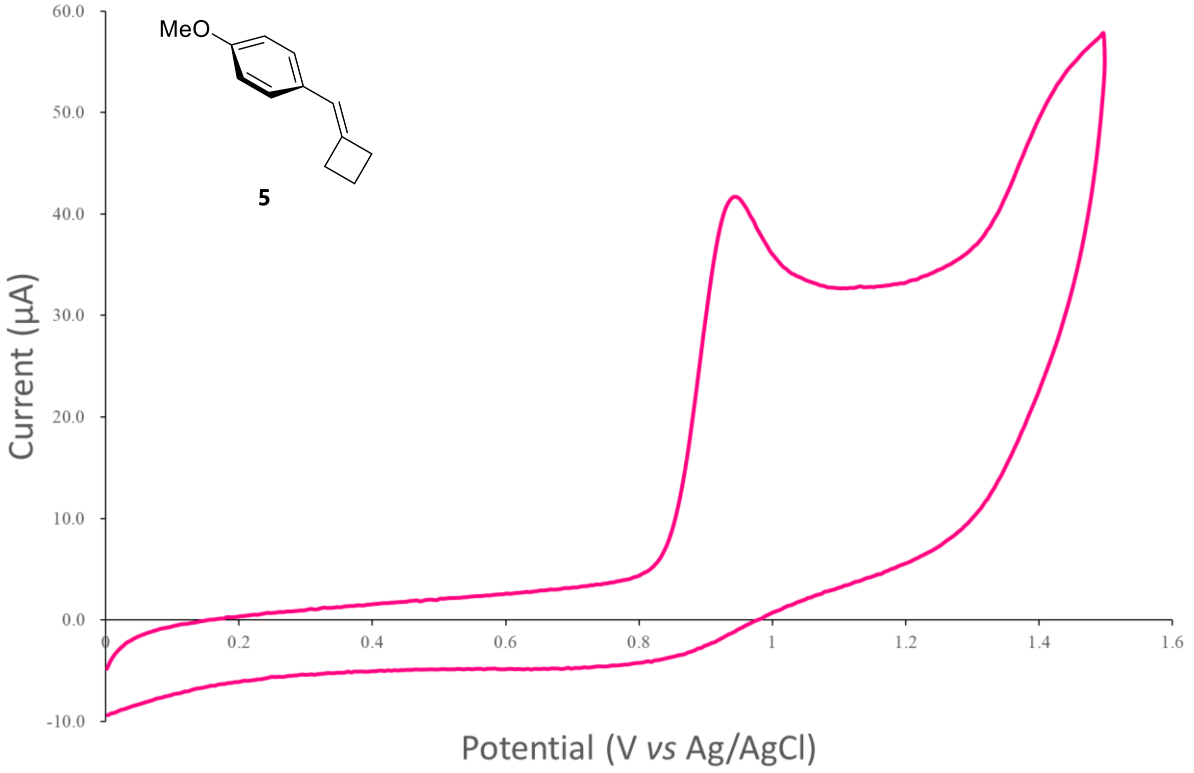

Figure S9. Cyclic voltammogram of the arylidene **5**.

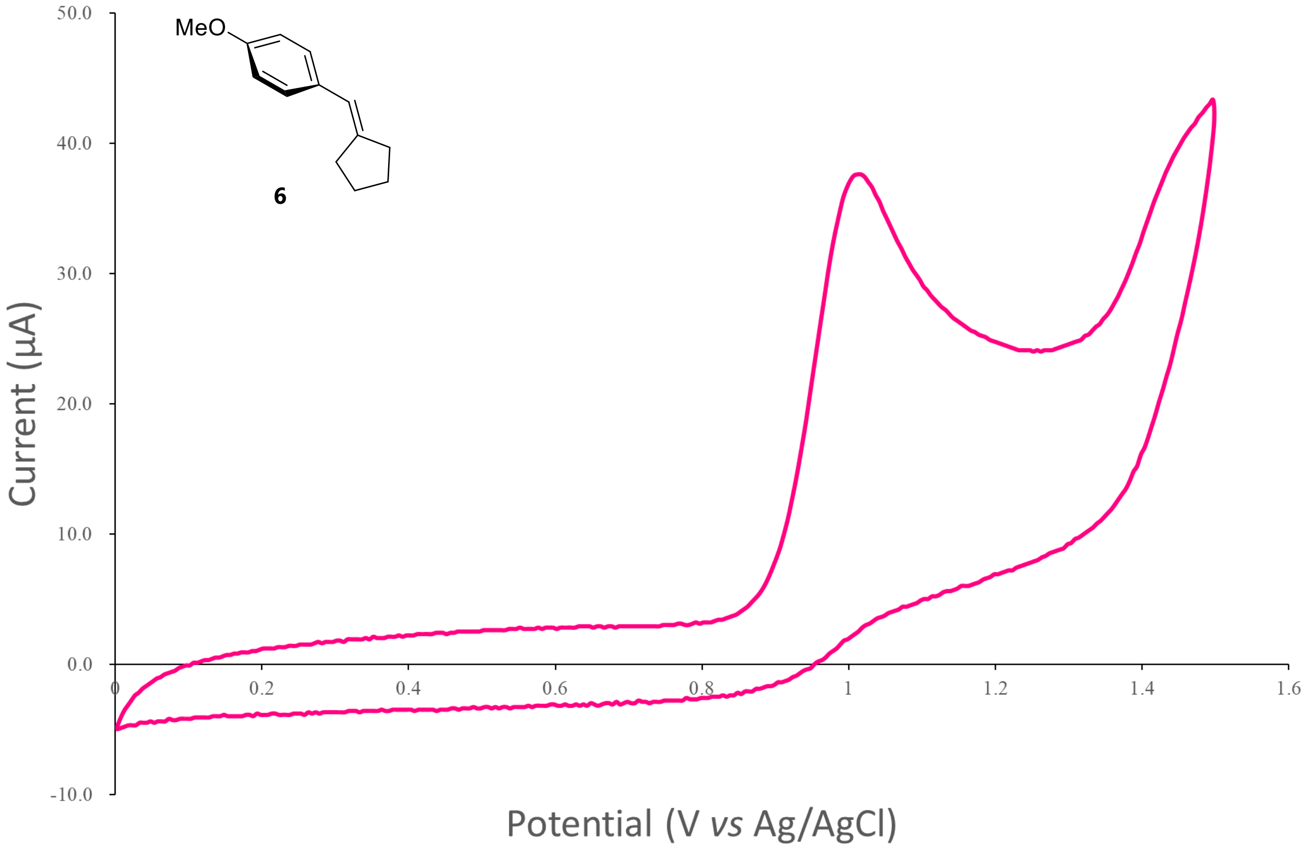

Figure S10. Cyclic voltammogram of the arylidene **6**.

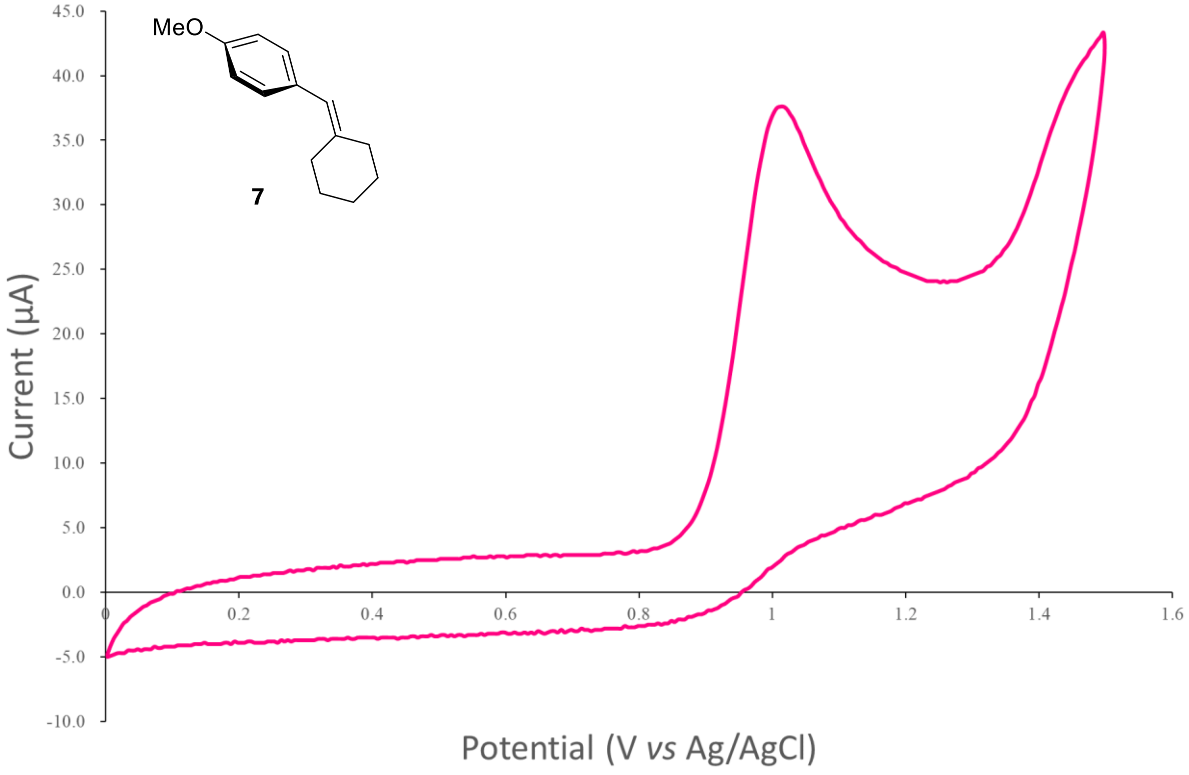

Figure S11. Cyclic voltammogram of the arylidene **7**.

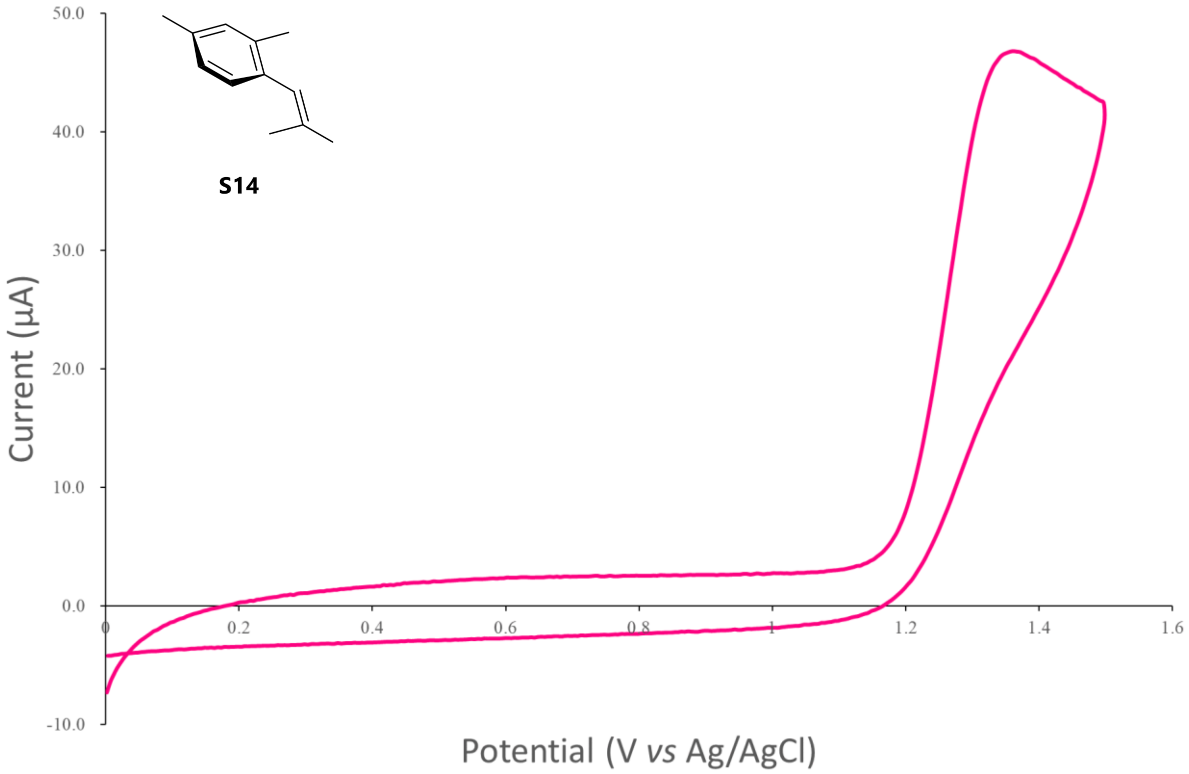

Figure S12. Cyclic voltammogram of the arylidene **S14**.

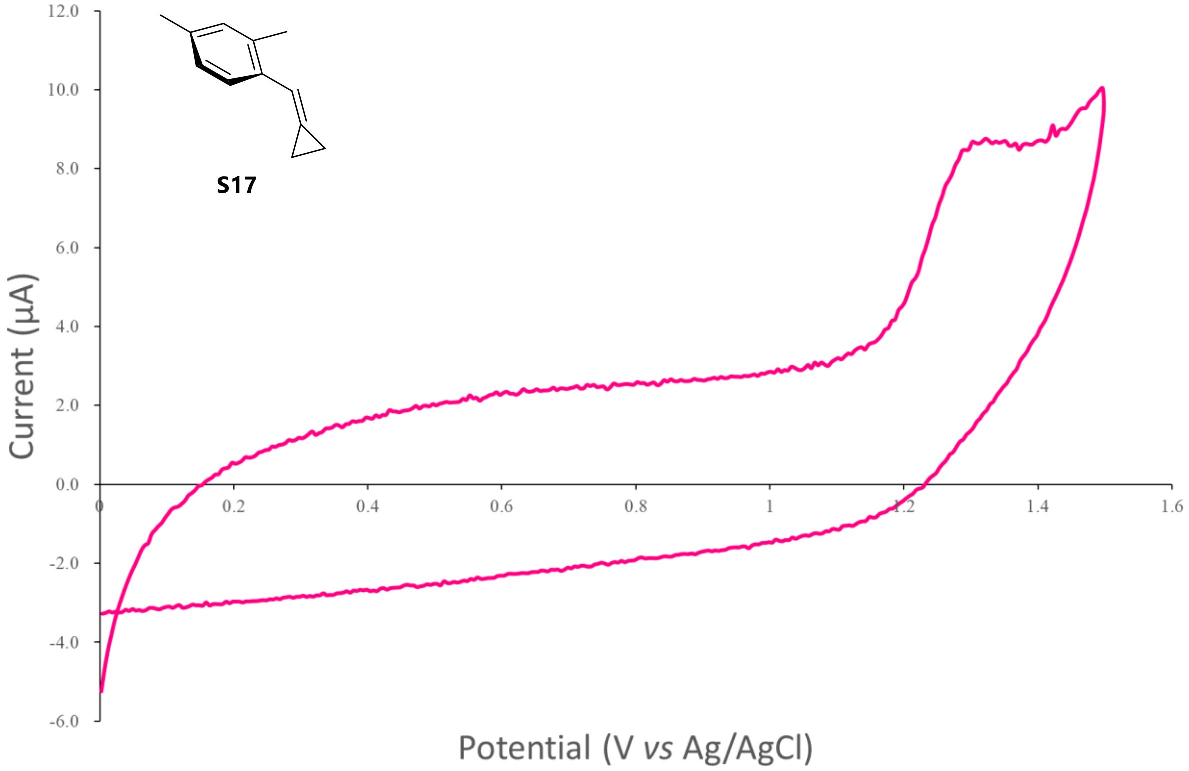

Figure S13. Cyclic voltammogram of the arylidene **S17**.

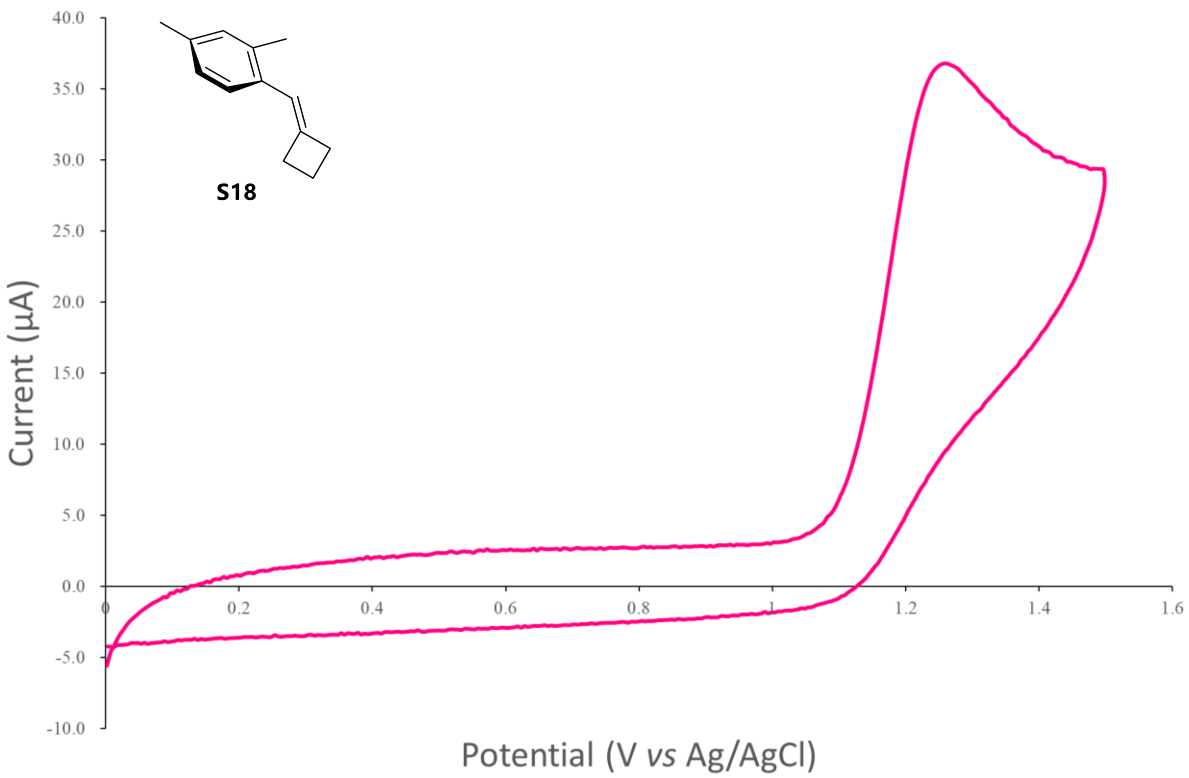

Figure S14. Cyclic voltammogram of the arylidene **S18**.

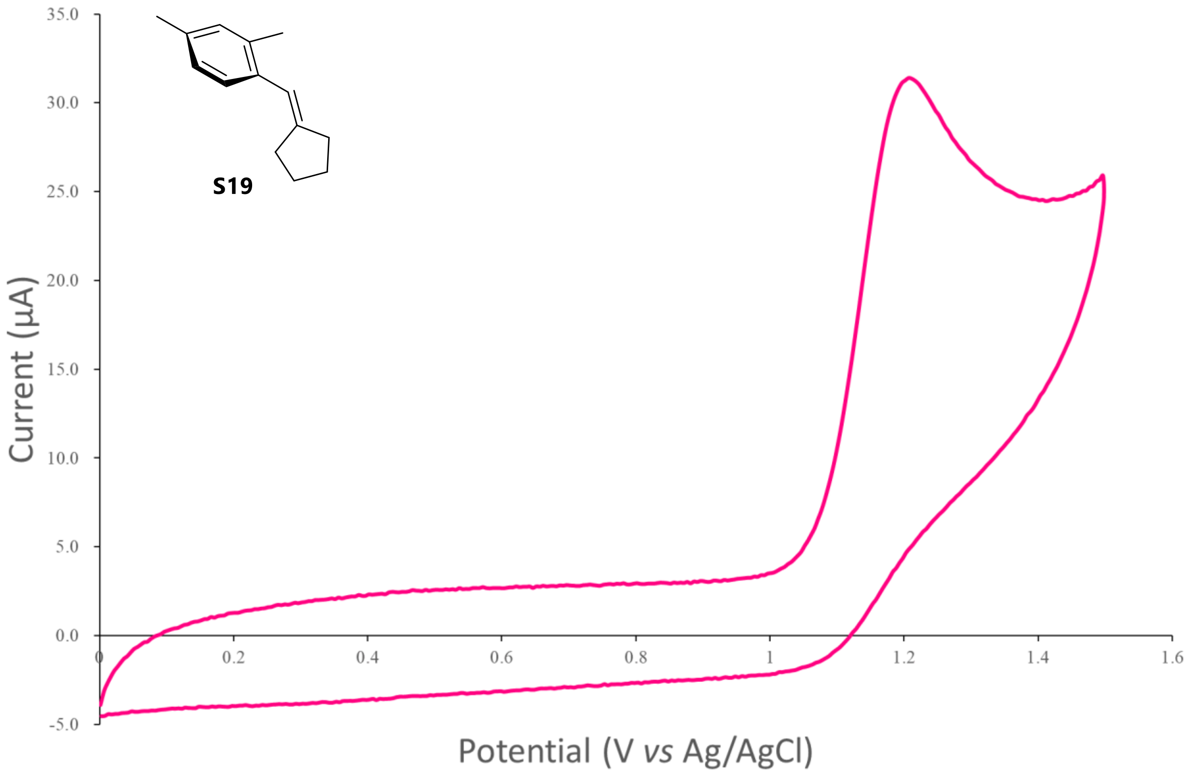

Figure S15. Cyclic voltammogram of the arylidene **S19**.

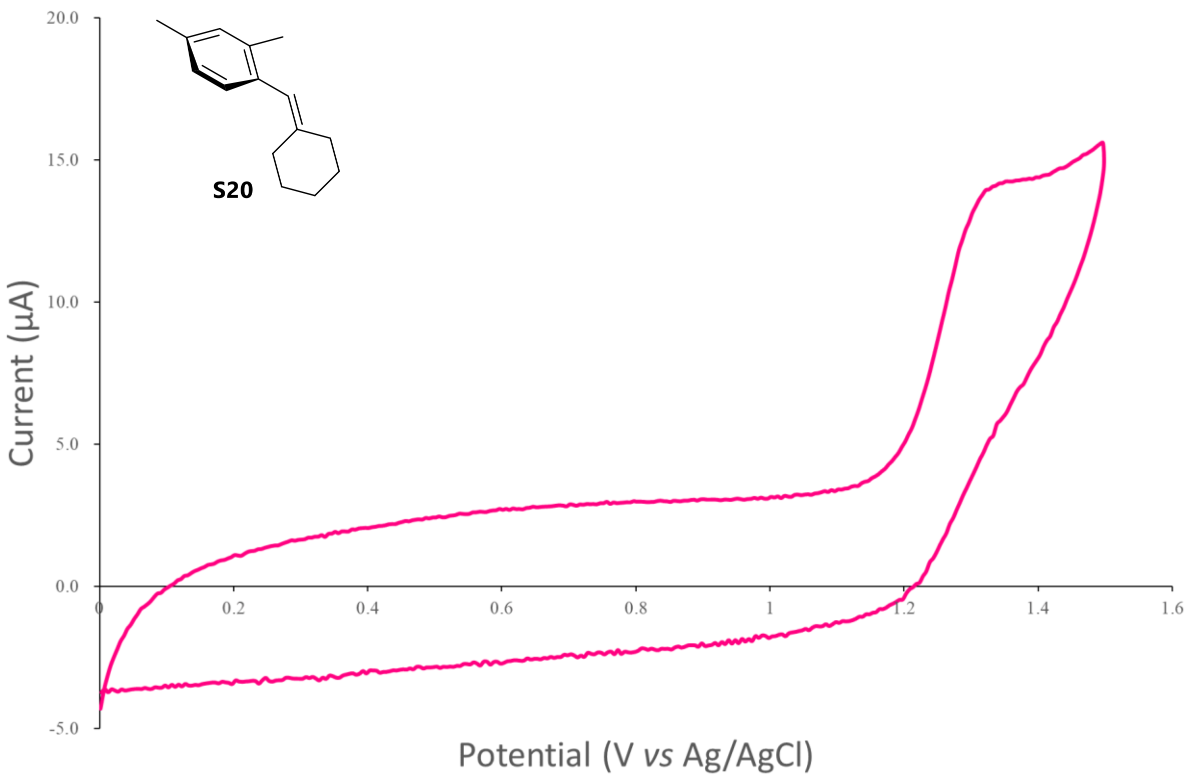

Figure S16. Cyclic voltammogram of the arylidene **S20**.

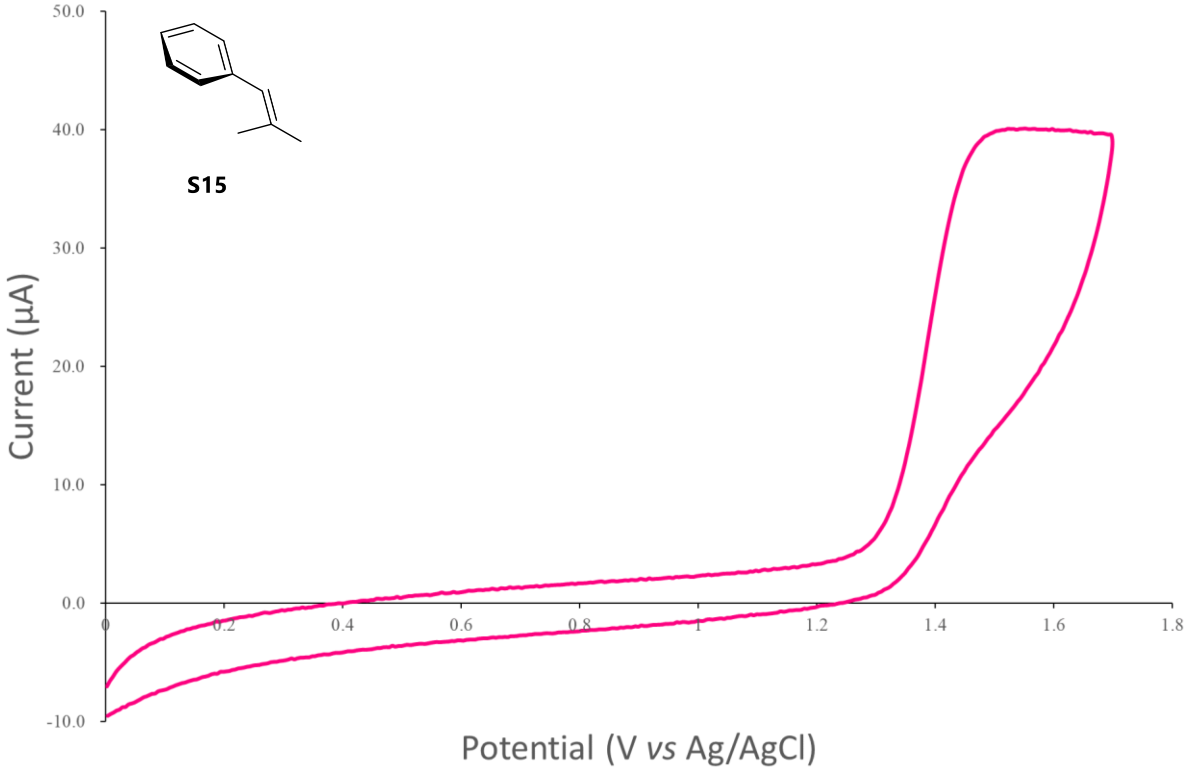

Figure S17. Cyclic voltammogram of the arylidene **S15**.

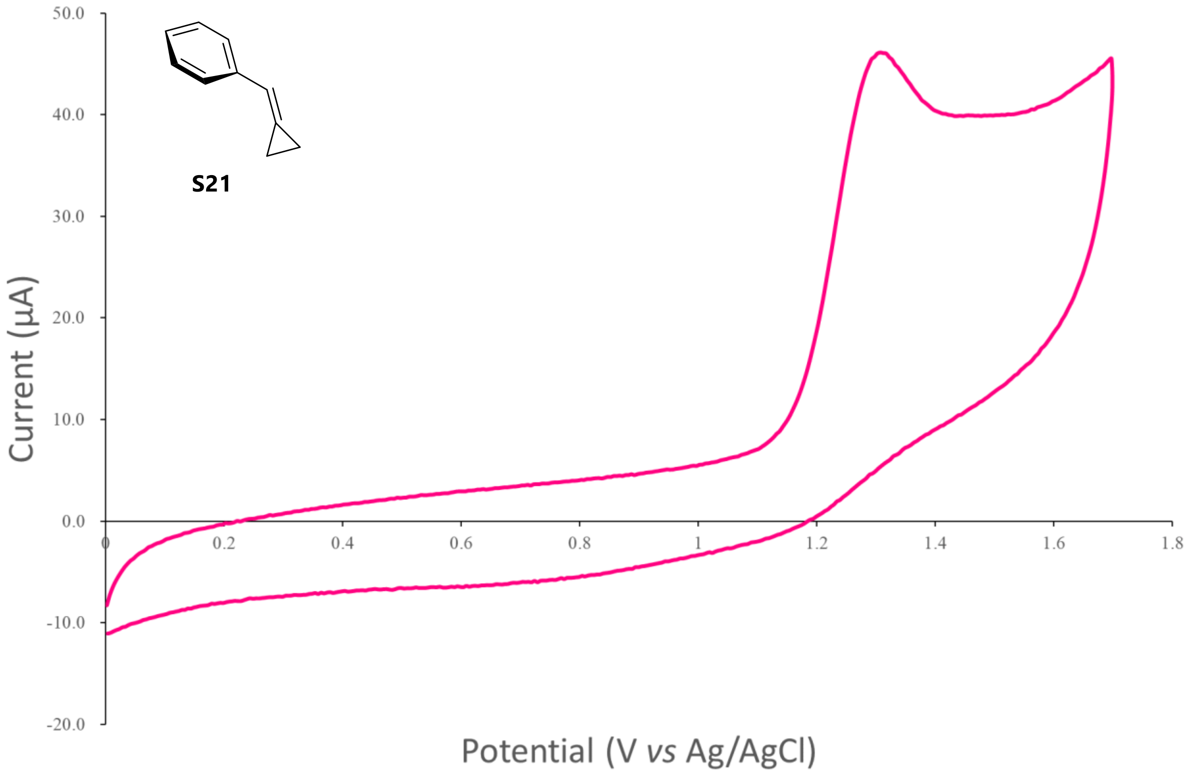

Figure S18. Cyclic voltammogram of the arylidene **S21**.

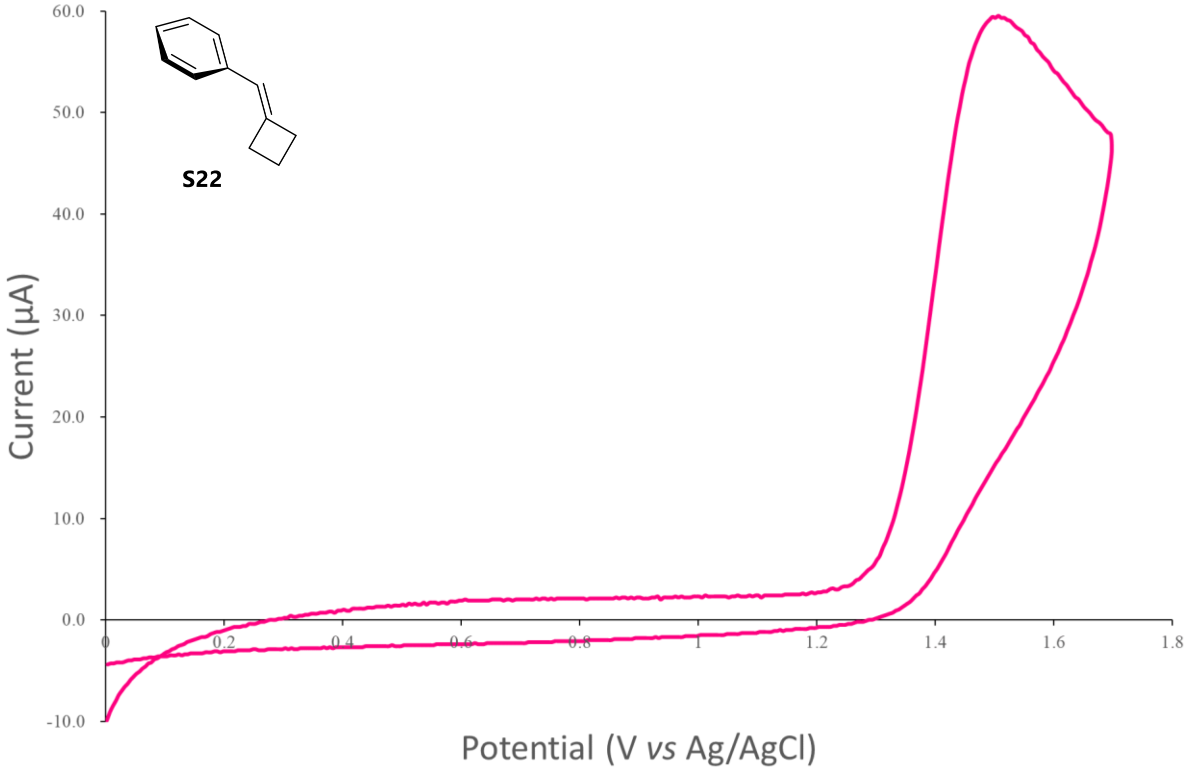

Figure S19. Cyclic voltammogram of the arylidene **S22**.

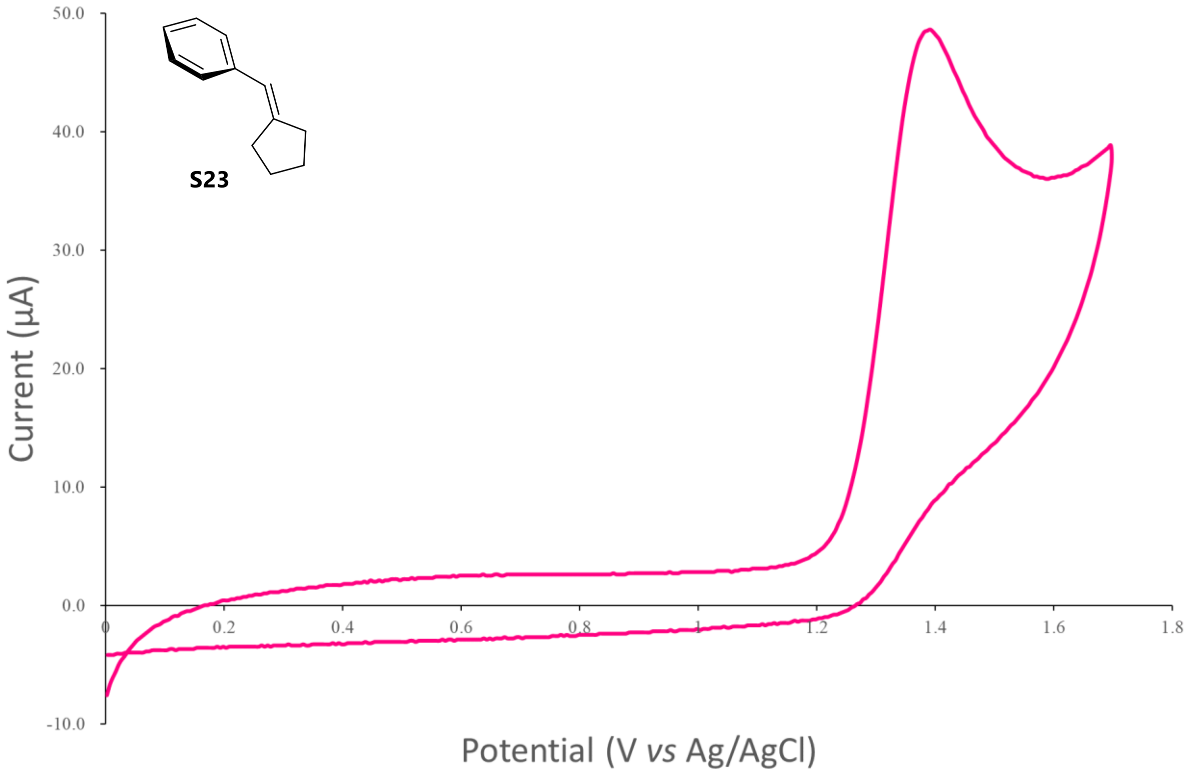

Figure S20. Cyclic voltammogram of the arylidene **S23**.

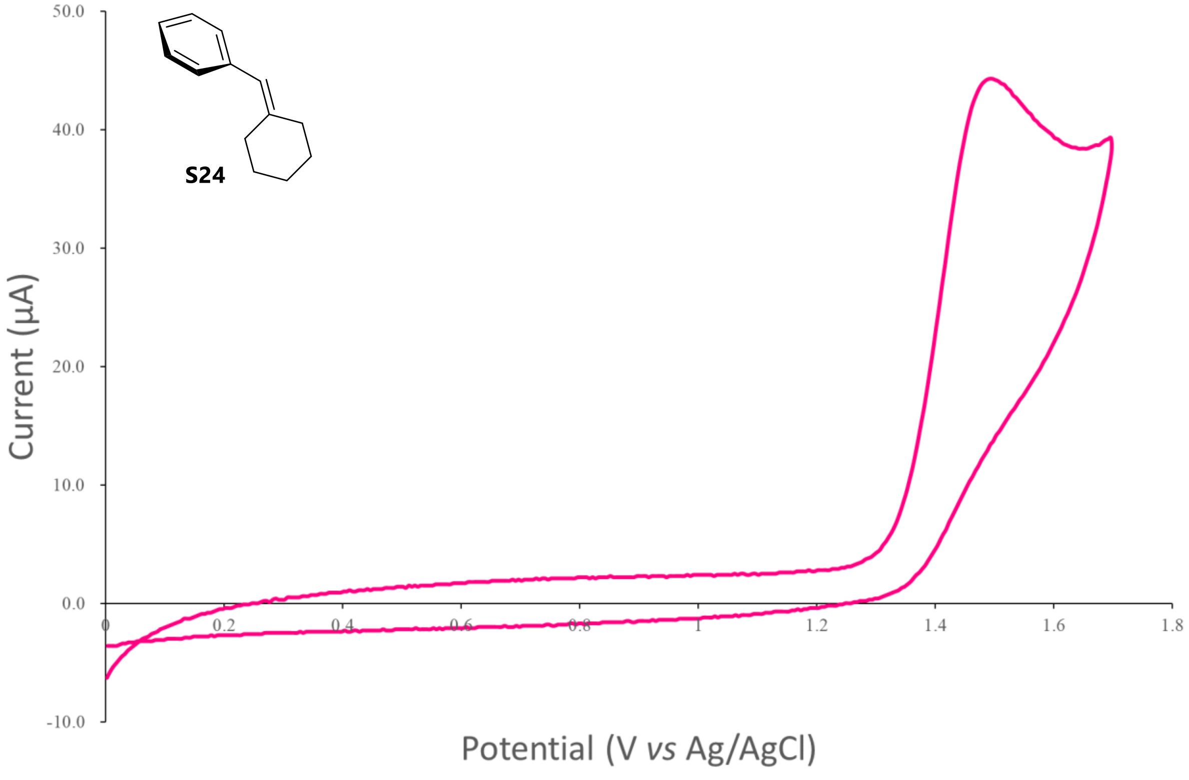

Figure S21. Cyclic voltammogram of the arylidene **S24**.

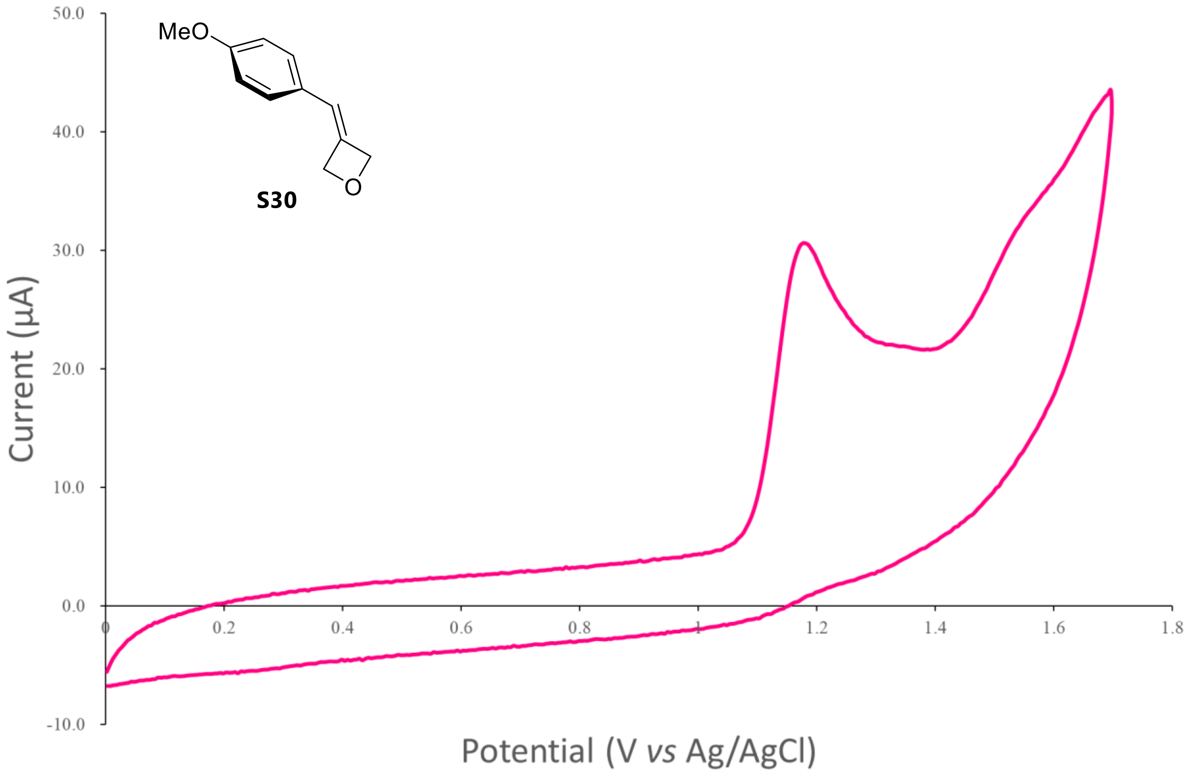

Figure S22. Cyclic voltammogram of the arylidene **S30**.

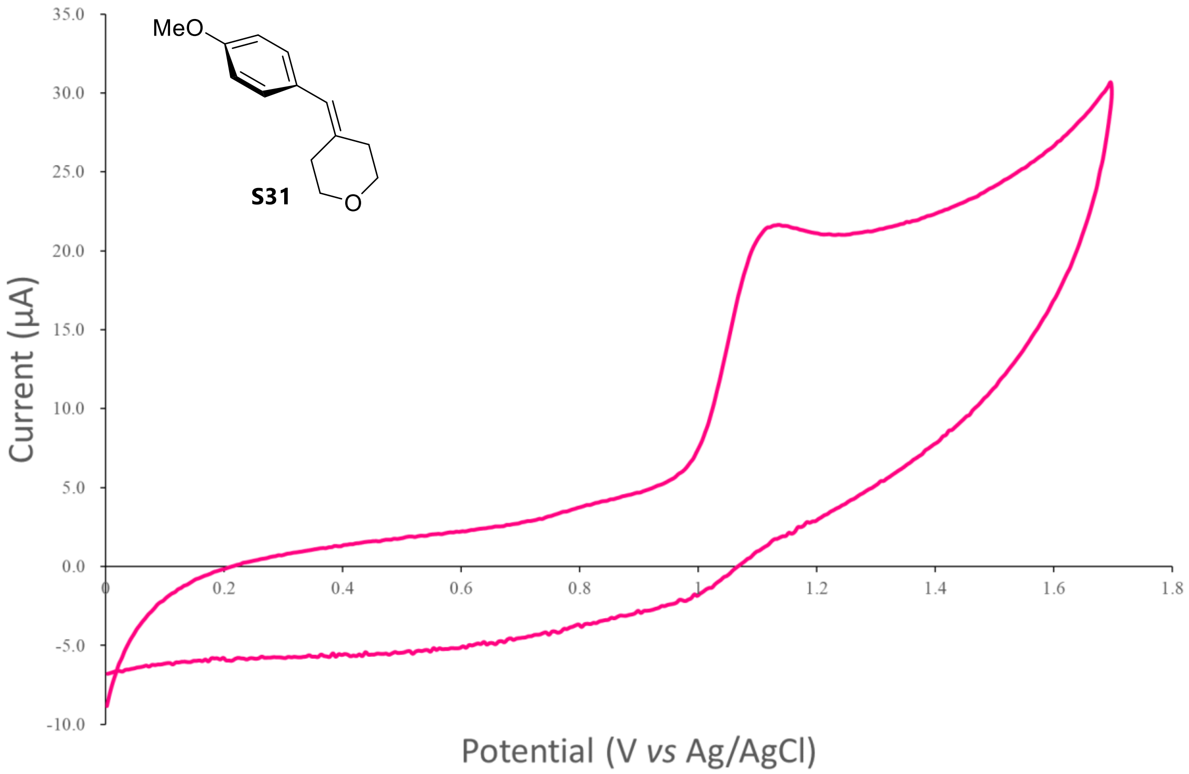

Figure S23. Cyclic voltammogram of the arylidene **S31**.

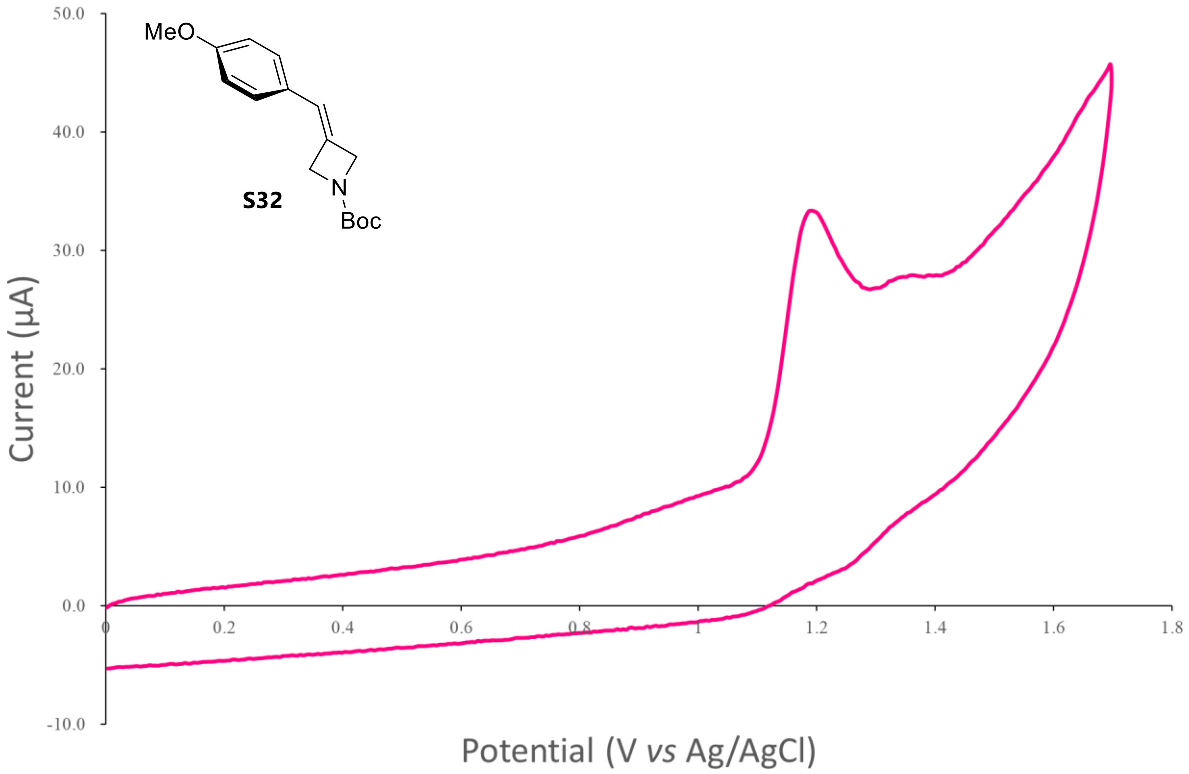

Figure S24. Cyclic voltammogram of the arylidene **S32**.

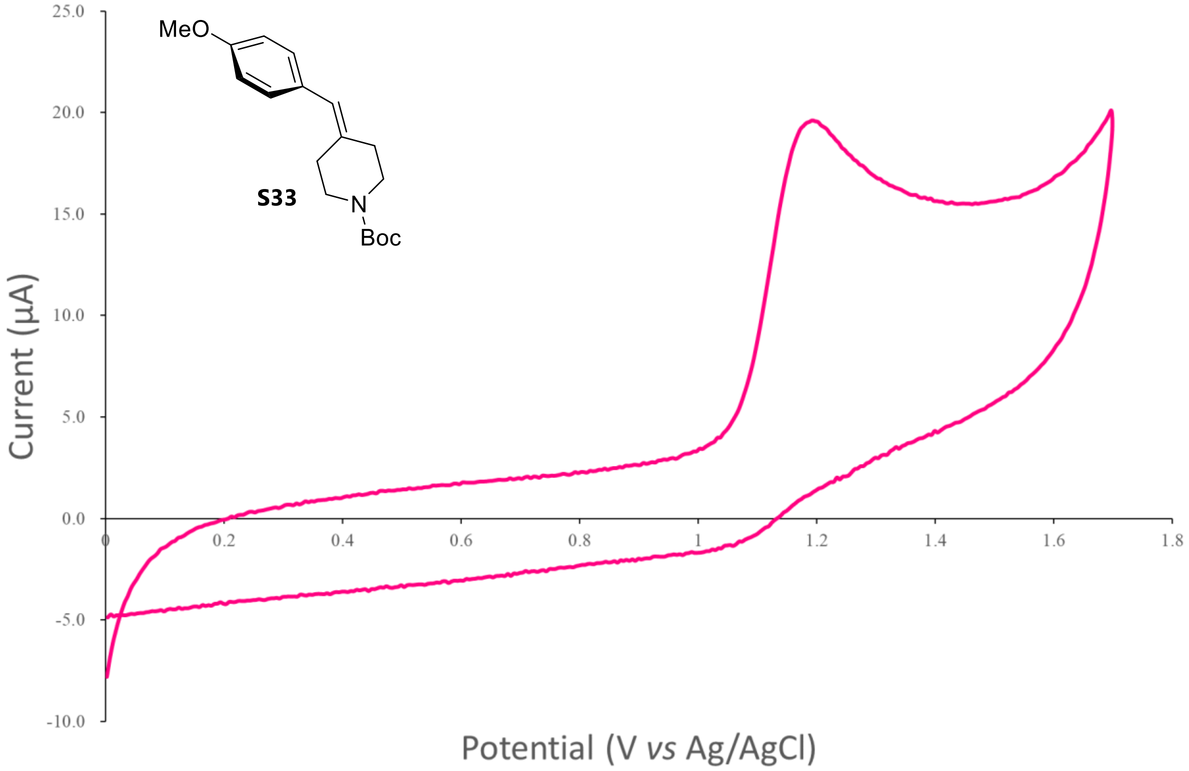

Figure S25. Cyclic voltammogram of the arylidene **S33**.

6. Characterization data, and copies of ^{1}H and ^{13}C NMR spectra

*1-Methoxy-4-(2-methylprop-1-en-1-yl)benzene (**1**).*[1] *Colorless oil.*

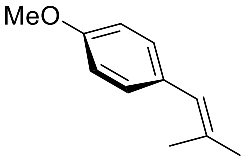

1

Product yield; 56% (458 mg, 2.8 mmol).

^{1}H NMR ($CDCl_3$, 500 MHz) δ 7.16 (2H, d, J = 8.6 Hz), 6.86 (2H, d, J = 8.6 Hz), 6.21 (1H, s), 3.80 (3H, s), 1.88 (3H, s), 1.84 (3H, s); $^{13}C\{^{1}H\}$ NMR (125 MHz, $CDCl_3$) δ 157.9, 134.0, 131.5, 130.0, 124.8, 113.7, 55.3, 27.0, 19.5; HRMS $[M + H]^+$ calculated for $C_{11}H_{15}O$ 163.1117, found 163.1132.

*1-(Cyclopropylidenemethyl)-4-methoxybenzene (**4**). Yellow oil.*

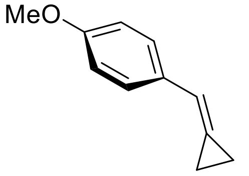

4

Product yield; 79% (633 mg, 3.9 mmol).

^{1}H NMR ($CDCl_3$, 500 MHz) δ 7.47 (2H, d, J = 8.6 Hz), 6.87 (2H, d, J = 8.6 Hz), 6.69 (1H, m), 3.82 (3H, s), 1.38 (2H, dt, J = 7.5, 1.7 Hz), 1.15 (2H, dt, J = 7.5, 1.7 Hz); $^{13}C\{^{1}H\}$ NMR (125 MHz, $CDCl_3$) δ 158.8, 131.5, 127.9, 122.0, 117.8, 114.2, 55.5, 4.3, 0.8; HRMS $[M + H]^+$ calculated for $C_{11}H_{13}O$ 161.0961, found 161.0977.

*1-(Cyclobutylidenemethyl)-4-methoxybenzene (**5**).*[2] *White solid.*

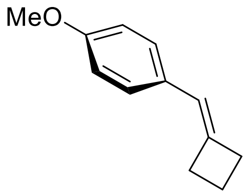

5

Product yield; 64% (560 mg, 3.2 mmol).

^{1}H NMR (CDCl$_3$, 500 MHz) δ 8.14 (2H, d, J = 8.6 Hz), 6.84 (2H, d, J = 8.6 Hz), 6.02 (1H, quint, J = 2.3 Hz), 3.80 (3H, s), 3.01 (2H, t, J = 7.5 Hz), 2.86 (2H, t, J = 7.5 Hz), 2.10 (2H, quint, J = 8.0 Hz); ^{13}C{^{1}H} NMR (125 MHz, CDCl$_3$) δ 157.9, 142.4, 131.2, 128.4, 120.4, 114.0, 55.5, 32.8, 32.7, 18.6; HRMS [M + H]$^+$ calculated for $C_{12}H_{15}O$ 175.1117, found 175.1140.

*1-(Cyclopentylidenemethyl)-4-methoxybenzene (**6**).*[1] *Colorless oil.*

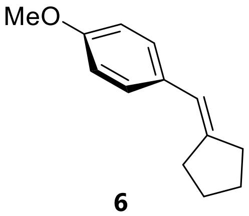

6

Product yield; 25% (181 mg, 1.0 mmol).

^{1}H NMR (CDCl$_3$, 500 MHz) δ 7.24 (2H, d, J = 8.6 Hz), 6.86 (2H, d, J = 8.6 Hz), 6.30 (1H, s), 3.81 (3H, s), 2.52 (2H, t, J = 6.9 Hz), 2.46 (2H, t, J = 6.9 Hz), 1.77 (2H, quint, J = 6.9 Hz), 1.65 (2H, quint, J = 6.9 Hz); ^{13}C{^{1}H} NMR (125 MHz, CDCl$_3$) δ 157.7, 144.9, 132.0, 129.2, 120.3, 113.8, 55.4, 36.1, 31.2, 27.5, 26.0; HRMS [M + H]$^+$ calculated for $C_{13}H_{17}O$ 189.1274, found 189.1298.

1-(Cyclohexylidenemethyl)-4-methoxybenzene (7).[1] *Colorless oil.*

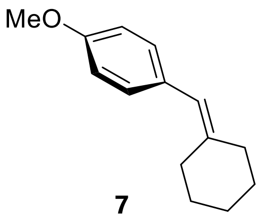

7

Product yield; 98% (989 mg, 4.9 mmol).

^{1}H NMR ($CDCl_3$, 500 MHz) δ 7.13 (2H, d, J = 8.6 Hz), 6.85 (2H, d, J = 8.6 Hz), 6.16 (1H, s), 3.80 (3H, s), 2.36 (2H, t, J = 5.7 Hz), 2.24 (2H, t, J = 5.7 Hz), 1.61 (4H, m), 1.54 (2H, quint, J = 5.2 Hz); ^{13}C{^{1}H} NMR (125 MHz, $CDCl_3$) δ 157.9, 142.4, 131.2, 130.2, 121.6, 113.7, 55.4, 37.8, 29.6, 28.9, 28.1, 27.0; HRMS [M + H]$^+$ calculated for $C_{14}H_{19}$ 203.1430, found 203.1444.

*2,4-Dimethyl-1-(2-methylprop-1-en-1-yl)benzene (**S14**). Colorless oil.*

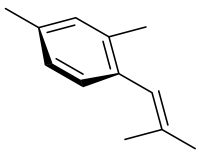

S14

Product yield; 55% (441 mg, 2.8 mmol).

^{1}H NMR ($CDCl_3$, 500 MHz) δ 7.02 (1H, d, J = 7.5 Hz), 6.99 (1H, s), 6.96 (1H, d, J = 7.5 Hz), 6.18 (1H, s), 2.30 (3H, s), 2.20 (3H, s), 1.90 (3H, s), 1.70 (3H, s); ^{13}C{^{1}H} NMR (125 MHz, $CDCl_3$) δ 136.4, 135.9, 135.2, 134.8, 130.7, 129.6, 126.2, 124.2, 26.4, 21.3, 20.1, 19.5; HRMS [M + H]$^+$ calculated for $C_{12}H_{17}$ 161.1325, found 161.1332.

*1-(Cyclopropylidenemethyl)-2,4-dimethylbenzene (**S17**). Yellow oil.*

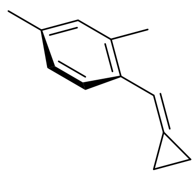

S17

Product yield; 34% (266 mg, 1.7 mmol).

^{1}H NMR ($CDCl_3$, 500 MHz) δ 7.64 (1H, d, J = 7.5 Hz), 6.98 (1H, d, J = 9.2 Hz), 6.97 (1H, s), 6.92 (1H, d, J = 1.7 Hz), 2.36 (3H, s), 2.30 (3H, s), 1.38 (2H, m), 1.16 (2H, m); ^{13}C{^{1}H} NMR (125 MHz, $CDCl_3$) δ 136.5, 135.0, 134.0, 131.3, 126.9, 126.1, 124.1, 115.6, 21.3, 19.9, 4.3, 1.1; HRMS [M + H]$^+$ calculated for $C_{12}H_{15}$ 159.1168, found 159.1153.

*1-(Cyclobutylidenemethyl)-2,4-dimethylbenzene (**S18**). Colorless oil.*

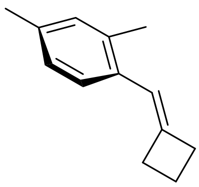

S18

Product yield; 61% (609 mg, 3.5 mmol).

^{1}H NMR ($CDCl_3$, 500 MHz) δ 7.11 (1H, d, J = 8.0 Hz), 6.96 (1H, s), 6.95 (1H, d, J = 8.0 Hz), 6.19 (1H, s), 2.95 (2H, t, J = 7.5 Hz), 2.87 (2H, t, J = 7.5 Hz), 2.29 (3H, s), 2.27 (3H, s), 2.07 (2H, quint, J = 7.5 Hz); ^{13}C{^{1}H} NMR (125 MHz, $CDCl_3$) δ 136.5, 135.0, 134.0, 131.3, 126.9, 126.1, 124.0, 115.6, 21.3, 19.9, 4.3, 1.1; HRMS [M + H]$^+$ calculated for $C_{13}H_{17}$ 173.1325, found 173.1347.

*1-(Cyclopentylidenemethyl)-2,4-dimethylbenzene (**S19**). Colorless oil.*

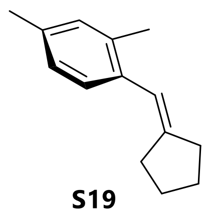

S19

Product yield; 11% (107 mg, 0.57 mmol).

^{1}H NMR ($CDCl_3$, 500 MHz) δ 7.19 (1H, d, J = 7.5 Hz), 6.97 (1H, s), 6.96 (1H, d, J = 7.5 Hz), 6.36 (1H, s), 2.47 (2H, t, J = 6.9 Hz), 2.40 (2H, t, J = 6.9 Hz), 2.30 (3H, s), 2.25 (3H, s), 1.69 (4H, m); ^{13}C{^{1}H} NMR (125 MHz, $CDCl_3$) δ 146.4, 135.9, 135.7, 135.2, 130.9, 128.2, 126.3, 118.8, 35.2, 31.0, 27.1, 25.9, 21.3, 20.2; HRMS [M + H]$^+$ calculated for $C_{14}H_{19}$ 187.1481, found 187.1489.

*1-(Cyclohexylidenemethyl)-2,4-dimethylbenzene (**S20**). Colorless oil.*

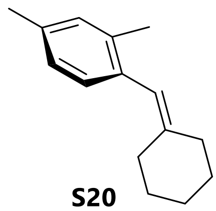

S20

Product yield; 20% (200 mg, 1 mmol).

^{1}H NMR ($CDCl_3$, 500 MHz) δ 6.99 (2H, d, J = 3.4 Hz), 6.94 (1H, d, J = 8.0 Hz), 6.11 (1H, s), 2.30 (3H, s), 2.26 (2H, t, J = 5.7 Hz), 2.20 (3H, s), 2.17 (2H, t, J = 5.7 Hz), 1.64 (2H, m), 1.58 (2H, m), 1.50 (2H, m); ^{13}C{^{1}H} NMR (125 MHz, $CDCl_3$) δ 142.9, 136.6, 136.0, 134.9, 130.7, 129.7, 126.1, 121.0, 37.6, 29.9, 29.1, 28.3, 27.1, 21.3, 20.2; HRMS [M + H]$^+$ calculated for $C_{15}H_{21}$ 201.1638, found 201.1646.

*(2-Methylprop-1-en-1-yl)benzene (**S15**).*[3] *Colorless oil.*

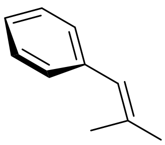

S15

Product yield; 97% (638 mg, 4.8 mmol).

^{1}H NMR ($CDCl_3$, 500 MHz) δ 7.31 (2H, t, J = 8.0 Hz), 7.22 (2H, d, J = 6.9 Hz), 7.18 (1H, t, J = 7.5 Hz), 6.27 (1H, s), 1.91 (3H, s), 1.86 (3H, s); ^{13}C{^{1}H} NMR (125 MHz, $CDCl_3$) δ 138.9, 135.7, 129.0, 128.3, 126.0, 125.4, 27.1, 19.7; HRMS [M + H]$^{+}$ calculated for $C_{10}H_{13}$ 133.1012, found 133.1036.

*(Cyclopropylidenemethyl)benzene (**S21**). Yellow oil.*

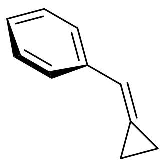

S21

Product yield; 42% (273 mg, 2.1 mmol).

^{1}H NMR ($CDCl_3$, 500 MHz) δ 7.54 (2H, d, J = 7.5 Hz), 7.33 (2H, t, J = 7.5 Hz), 7.21 (1H, t, J = 7.5 Hz), 6.76 (1H, t, J = 1.7 Hz), 1.43 (2H, dt, J = 8.0, 2.3 Hz), 1.18 (2H, dt, J = 8.0, 1.7 Hz); ^{13}C{^{1}H} NMR (125 MHz, $CDCl_3$) δ 138.5, 128.7, 126.9, 126.9, 124.5, 118.5, 4.5, 0.8; HRMS [M + H]$^{+}$ calculated for $C_{10}H_{11}$ 131.0855, found 131.0864.

*(Cyclobutylidenemethyl)benzene (**S22**).*[4] *Colorless oil.*

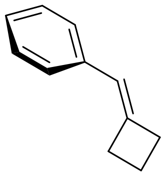

S22

Product yield; 75% (539 mg, 3.7 mmol).

^{1}H NMR (CDCl$_3$, 500 MHz) δ 7.29 (2H, t, J = 7.5 Hz), 7.20 (2H, d, J = 7.5 Hz), 7.14 (1H, t, J = 7.5 Hz),6.07 (1H, t, J = 2.3 Hz), 3.05 (2H, t, J = 8.0 Hz), 2.88 (2H, t, J = 8.0 Hz), 2.11 (2H, quint, J = 8.0 Hz); ^{13}C{^{1}H} NMR (125 MHz, CDCl$_3$) δ 145.0, 138.3, 128.6, 127.3, 126.0, 121.1, 33.0, 32.9, 18.6; HRMS [M + H]$^+$ calculated for $C_{11}H_{13}$ 145.1012, found 145.1013.

*(Cyclopentylidenemethyl)benzene (**S23**).*[4] *Colorless oil.*

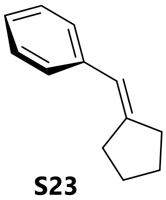

S23

Product yield; 9% (74 mg, 0.47 mmol).

^{1}H NMR (CDCl$_3$, 500 MHz) δ 7.31 (2H, d, J = 4.0 Hz), 7.30 (2H, s), 7.15 (1H, m), 6.36 (1H, t, J = 2.3 Hz), 2.55 (2H, t, J = 6.9 Hz), 2.49 (2H, t, J = 6.9 Hz), 1.78 (2H, quint, J = 6.9 Hz), 1.66 (2H, quint, J = 6.9 Hz); ^{13}C{^{1}H} NMR (125 MHz, CDCl$_3$) δ 147.4, 139.1, 128.4, 128.2, 125.9, 121.1, 36.2, 31.4, 27.5, 25.9; HRMS [M + H]$^+$ calculated for $C_{12}H_{15}$ 159.1168, found 159.1138.

*(Cyclohexylidenemethyl)benzene (**S24**).*[4] *Colorless oil.*

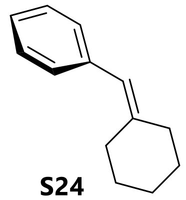

S24

Product yield; 36% (312 mg, 1.8 mmol).

^{1}H NMR ($CDCl_3$, 500 MHz) δ 7.30 (2H, t, J = 7.5 Hz), 7.20 (2H, d, J = 7.5 Hz), 7.18 (1H, t, J = 7.5 Hz), 6.23 (1H, s), 2.37 (2H, t, J = 5.7 Hz), 2.26 (2H, t, J = 5.7 Hz), 1.63 (4H, m), 1.55 (2H, m); ^{13}C{^{1}H} NMR (125 MHz, $CDCl_3$) δ 143.7, 138.7, 129.2, 128.3, 126.0, 122.2, 37.9, 29.7, 28.9, 28.1, 27.0; HRMS [M + H]$^{+}$ calculated for $C_{13}H_{17}$ 173.1325, found 173.1334.

*3-(4-Methoxybenzylidene)oxetane (**S30**).*[2] *Yellow solid.*

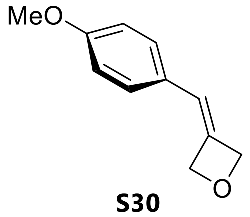

S30

Product yield; 25% (225 mg, 1.3 mmol).

^{1}H NMR ($CDCl_3$, 300 MHz) δ 6.94 (2H, d, J = 8.9 Hz), 6.87 (2H, d, J = 8.9 Hz), 6.04 (1H, t, J = 2.4 Hz), 5.55 (2H, q, J = 2.8 Hz), 5.38 (2H, q, J = 2.8 Hz), 3.81 (3H, s); ^{13}C{^{1}H} NMR (125 MHz, $CDCl_3$) δ 158.7, 134.5, 129.1, 128.5, 119.2, 114.4, 80.6, 80.4, 55.5; HRMS [M + H]$^{+}$ calculated for $C_{11}H_{13}O_2$ 177.0910, found 177.0920.

*4-(4-Methoxybenzylidene)tetrahydro-2H-pyran (**S31**).*[1] *Yellow solid.*

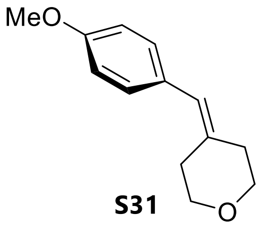

S31

Product yield; 18% (184 mg, 0.90 mmol).

^{1}H NMR (CDCl$_3$, 300 MHz) δ 7.14 (2H, d, J = 8.6 Hz), 6.86 (2H, d, J = 8.6 Hz), 6.27 (1H, s), 3.81 (3H, s), 3.78 (2H, t, J = 5.9 Hz), 3.66 (2H, t, J = 5.9 Hz), 2.53 (2H, dt, J = 5.9, 1.4 Hz), 2.38 (2H, dt, J = 5.9, 1.4 Hz); ^{13}C{^{1}H} NMR (125 MHz, CDCl$_3$) δ 158.2, 136.5, 130.2, 130.1, 123.5, 113.8, 69.7, 68.7, 55.4, 37.4, 30.8; HRMS [M + H]$^+$ calculated for $C_{13}H_{17}O_2$ 205.1223, found 205.1230.

*tert-Butyl 3-(4-methoxybenzylidene)azetidine-1-carboxylate (**S32**).*[2] *Yellow solid.*

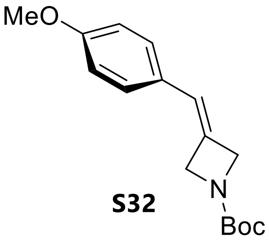

S32

Product yield; 43% (597 mg, 2.2 mmol).

^{1}H NMR (CDCl$_3$, 300 MHz) δ 7.05 (2H, d, J = 8.9 Hz), 6.88 (2H, d, J = 8.9 Hz), 6.20 (1H, t, J = 2.1 Hz), 4.81 (2H, q, J = 2.8 Hz), 4.62 (2H, q, J = 2.8 Hz), 3.81 (3H, s), 1.48 (9H, s); ^{13}C{^{1}H} NMR (125 MHz, CDCl$_3$) δ 158.8, 156.6, 129.2, 128.6, 128.1, 121.9, 114.4, 80.0, 55.5, 28.7; HRMS [M + H]$^+$ calculated for $C_{16}H_{22}NO_3$ 276.1594, found 276.1611.

*tert-Butyl 4-(4-methoxybenzylidene)piperidine-1-carboxylate (**S33**).*[5] *White solid.*

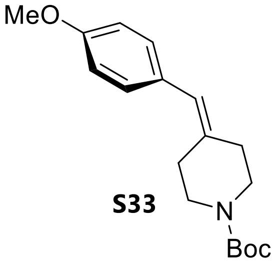

S33

Product yield; 26% (402 mg, 1.3 mmol).

^{1}H NMR ($CDCl_3$, 500 MHz) δ 7.12 (2H, d, J = 8.6 Hz), 6.86 (2H, d, J = 8.6 Hz), 6.30 (1H, s), 3.80 (3H, s), 3.50 (2H, t, J = 5.2 Hz), 3.40 (2H, t, J = 5.2 Hz), 2.45 (2H, t, J = 5.2 Hz), 2.31 (2H, t, J = 5.2 Hz), 1.48 (9H, s); ^{13}C{^{1}H} NMR (125 MHz, $CDCl_3$) δ 158.3, 155.1, 137.3, 130.3, 124.2, 113.9, 79.8, 55.5, 36.4, 29.4, 28.7; HRMS [M + H]$^+$ calculated for $C_{18}H_{26}NO_3$ 304.1907, found 304.1887.

*4'-Methoxy-2,2,4,5-tetramethyl-1,2,3,6-tetrahydro-1,1'-biphenyl (**3**). Colorless oil.*

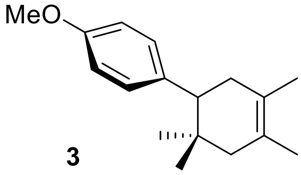

3

^{1}H NMR ($CDCl_3$, 500 MHz) δ 7.11 (2H, d, J = 8.6 Hz), 6.81 (2H, d, J = 8.6 Hz), 3.79 (3H, s), 2.58 (1H, dd, J = 9.7, 5.7 Hz), 2.31 (1H, dd, J = 17.2, 10.3 Hz), 2.12 (1H, dd, J = 17.2, 3.4 Hz), 1.94 (1H, d, J = 17.2 Hz), 1.72 (1H, d, J = 17.8 Hz), 1.65 (3H, s), 1.64 (3H, s), 0.78 (3H, s), 0.77 (3H, s); ^{13}C{^{1}H} NMR (125 MHz, $CDCl_3$) δ 158.1, 136.2, 130.1, 124.8, 124.6, 113.3, 55.5, 49.5, 47.6, 36.6, 33.4, 29.8, 22.6, 19.4, 18.9; HRMS [M + H]$^+$ calculated for $C_{17}H_{25}O$ 245.1900, found 245.1928.

*9-(4-Methoxyphenyl)-6,7-dimethylspiro[3.5]non-6-ene (**9**). Yellow oil.*

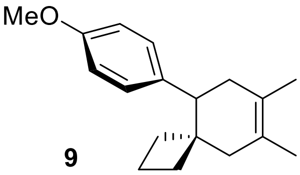

9

^{1}H NMR (CDCl$_3$, 500 MHz) δ 7.11 (2H, d, J = 8.6 Hz), 6.81 (2H, d, J = 8.6 Hz), 3.79 (3H, s), 2.78 (1H, t, J = 5.7 Hz), 2.33 (1H, dd, J = 18.3, 2.9 Hz), 2.12 (1H, dd, J = 17.8, 2.3 Hz), 2.01 (3H, m), 1.81 (2H, m), 1.66 (6H, m), 1.62 (2H, m), 1.41 (1H, m); ^{13}C{^{1}H} NMR (125 MHz, CDCl$_3$) δ 158.1, 136.4, 129.5, 125.2, 124.7, 113.4, 55.4, 47.7, 42.8, 42.2, 35.0, 32.8, 29.8, 19.6, 19.0, 15.3; HRMS [M + H]$^+$ calculated for $C_{18}H_{25}O$ 257.1900, found 257.1899.

*5-(4-Methoxyphenyl)-2,3-dimethylspiro[5.5]undec-2-ene (**11**). Colorless oil.*

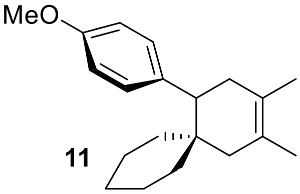

11

^{1}H NMR (CDCl$_3$, 500 MHz) δ 7.09 (2H, d, J = 8.0 Hz), 6.79 (2H, d, J = 8.0 Hz), 3.79 (3H, s), 2.63 (1H, t, J = 5.7 Hz), 2.27 (1H, dd, J = 17.8, 3.4 Hz), 2.15 (1H, dd, J = 17.2, 4.6 Hz), 1.98 (1H, d, J = 17.8 Hz), 1.78 (1H, d, J = 17.2 Hz), 1.68 (3H, s), 1.65 (3H, s), 1.46 (5H, m), 1.31 (1H, m), 1.13 (4H, m); ^{13}C{^{1}H} NMR (125 MHz, CDCl$_3$) δ 158.0, 136.7, 130.2, 130.1, 124.6, 113.2, 55.4, 39.4, 36.9, 36.0, 35.5, 32.4, 30.0, 26.8, 22.2, 22.1, 19.8, 18.9; HRMS [M + H]$^+$ calculated for $C_{20}H_{29}O$ 285.2213, found 285.2220.

*2,2,2',4,4',5-Hexamethyl-1,2,3,6-tetrahydro-1,1'-biphenyl (**12**). Colorless oil.*

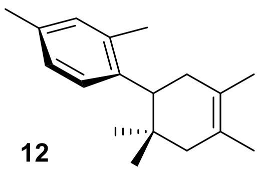

12

^{1}H NMR ($CDCl_3$, 500 MHz) δ 7.16 (1H, d, J = 8.6 Hz), 6.96 (1H, s), 6.95 (1H, d, J = 6.9 Hz), 3.01 (1H, dd, J = 9.7, 5.7 Hz), 2.31 (3H, s), 2.28 (3H, s), 2.26 (1H, m), 2.05 (1H, dd, J = 19.5, 4.6 Hz), 2.01 (1H, d, J = 17.2 Hz), 1.77 (1H, d, J = 16.6 Hz), 1.65 (3H, s), 1.64 (3H, s), 0.86 (3H, s), 0.80 (3H, s); ^{13}C{^{1}H} NMR (125 MHz, $CDCl_3$) δ 139.9, 136.9, 135.2, 131.2, 127.5, 126.5, 125.1, 124.8, 48.5, 43.1, 37.5, 34.3, 28.9, 22.6, 21.2, 21.2, 19.5, 18.9; HRMS [M + H]$^+$ calculated for $C_{18}H_{27}$ 243.2107, found 243.2104.

*9-(2,4-Dimethylphenyl)-6,7-dimethylspiro[3.5]non-6-ene (**14**). Colorless oil.*

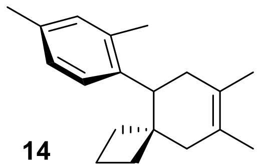

14

^{1}H NMR ($CDCl_3$, 500 MHz) δ 7.17 (1H, d, J = 7.5 Hz), 7.00 (1H, d, J = 8.6 Hz), 6.99 (1H, s), 3.07 (1H, dd, J = 9.7, 5.2 Hz), 2.32 (2H, m), 2.30 (3H, s), 2.23 (3H, m), 2.21 (1H, m), 1.95 (2H, m), 1.80 (1H, m), 1.68 (3H, s), 1.64 (3H, s), 1.62 (1H, m), 1.53 (1H, m), 1.46 (2H, m); ^{13}C{^{1}H} NMR (125 MHz, $CDCl_3$) δ 139.2, 137.3, 135.3, 131.2, 127.0, 126.8, 126.3, 124.5, 46.1, 42.9, 41.8, 35.2, 31.2, 26.9, 21.2, 20.9, 19.4, 19.1, 15.9; HRMS [M + H]$^+$ calculated for $C_{19}H_{27}$ 255.2107, found 255.2114.

*10-(2,4-Dimethylphenyl)-7,8-dimethylspiro[4.5]dec-7-ene (**15**). Colorless oil.*

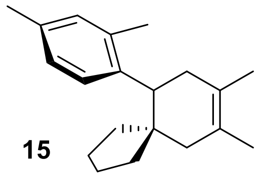

15

^{1}H NMR ($CDCl_3$, 500 MHz) δ 7.17 (1H, d, J = 8.0 Hz), 6.96 (1H, s), 6.95 (1H, d, J = 8.0 Hz), 3.10 (1H, dd, J = 8.6, 4.3 Hz), 2.29 (3H, s), 2.28 (3H, s), 2.27 (1H, m), 2.12 (1H, dd, J = 17.2, 4.0 Hz), 1.97 (1H, d, J = 16.0 Hz), 1.89 (1H, d, J = 17.8 Hz), 1.65 (6H, s), 1.55 (3H, m), 1.40 (3H, m), 1.24 (2H, m); $^{13}C\{^{1}H\}$ NMR (125 MHz, $CDCl_3$) δ 140.1, 137.1, 135.2, 131.1, 127.3, 126.7, 126.1, 125.0, 46.4, 45.0, 41.8, 37.9, 37.7, 31.9, 24.5, 23.9, 21.1, 19.6, 19.0; HRMS $[M + H]^+$ calculated for $C_{20}H_{29}$ 269.2264, found 269.2247.

*5-(2,4-Dimethylphenyl)-2,3-dimethylspiro[5.5]undec-2-ene (**16**). Colorless oil.*

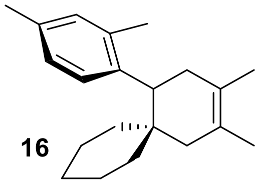

16

^{1}H NMR ($CDCl_3$, 500 MHz) δ 7.15 (1H, d, J = 8.6 Hz), 6.96 (1H, s), 6.95 (1H, d, J = 6.9 Hz), 2.99 (1H, dd, J = 8.6, 6.3 Hz), 2.31 (3H, s), 2.28 (3H, s), 2.22 (1H, m), 2.20 (1H, d, J = 16.6 Hz), 2.08 (1H, dd, J = 17.8, 4.6 Hz), 1.90 (1H, d, J = 17.8 Hz), 1.69 (3H, s), 1.63 (3H, s), 1.55 (1H, m), 1.45 (2H, m), 1.40 (3H, m), 1.31 (1H, tt, J = 13.2, 3.4 Hz), 1.26 (1H, m), 0.98 (2H, m); $^{13}C\{^{1}H\}$ NMR (125 MHz, $CDCl_3$) δ 139.8, 137.2, 135.1, 131.1, 128.0, 126.5, 125.3, 124.4, 44.6, 39.6, 36.8, 36.6, 30.0, 29.4, 26.6, 22.1, 22.1, 21.5, 21.2, 19.7, 18.9; HRMS $[M + H]^+$ calculated for $C_{21}H_{31}$ 283.2420, found 283.2420.

*2,2,4,5-Tetramethyl-1,2,3,6-tetrahydro-1,1'-biphenyl (**17**). Colorless oil.*

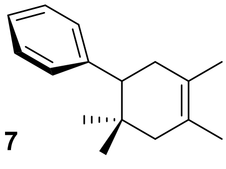

17

^{1}H NMR ($CDCl_3$, 500 MHz) δ 7.25 (2H, d, J = 7.5 Hz), 7.19 (3H, m), 2.63 (1H, dd, J = 9.7, 5.7 Hz), 2.36 (1H, m), 2.14 (1H, dd, J = 16.6, 4.0 Hz), 1.96 (1H, d, J = 17.2 Hz), 1.74 (1H, d, J = 16.6 Hz), 1.66 (3H, s), 1.65 (3H, s), 0.80 (3H, s), 0.79 (3H, s); $^{13}C\{^1H\}$ NMR (125 MHz, $CDCl_3$) δ 144.2, 129.3, 127.9, 126.3, 124.8, 124.6, 50.4, 47.6, 36.4, 33.3, 29.8, 22.6, 19.4, 18.9; HRMS $[M + H]^+$ calculated for $C_{16}H_{23}$ 215.1794, found 215.1783.

*6,7-Dimethyl-9-phenylspiro[3.5]non-6-ene (**19**). Colorless oil.*

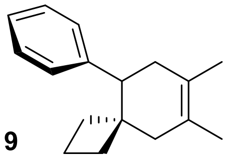

19

^{1}H NMR ($CDCl_3$, 500 MHz) δ 7.26 (2H, m), 7.19 (3H, m), 2.84 (1H, t, J = 5.7 Hz), 2.35 (1H, d, J = 16.6 Hz), 2.16 (1H, d, J = 15.5 Hz), 2.04 (3H, m), 1.83 (2H, m), 1.67 (3H, s), 1.66 (1H, m), 1.66 (3H, s), 1.62 (1H, m), 1.41 (1H, m); $^{13}C\{^1H\}$ NMR (125 MHz, $CDCl_3$) δ 144.3, 128.7, 128.1, 126.2, 125.2, 124.8, 48.5, 42.7, 42.1, 34.8, 33.0, 29.9, 19.6, 19.0, 15.3; HRMS $[M + H]^+$ calculated for $C_{17}H_{23}$ 227.1794, found 227.1780.

*7,8-Dimethyl-10-phenylspiro[4.5]dec-7-ene (**20**). Colorless oil.*

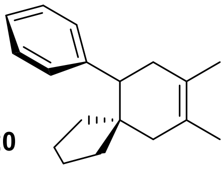

20

^{1}H NMR ($CDCl_3$, 500 MHz) δ 6.30 (2H, t, J = 6.3 Hz), 7.19 (1H, s), 7.18 (2H, d, J = 7.5 Hz), 2.70 (1H, t, J = 5.7 Hz), 2.35 (1H, m), 2.22 (1H, m), 1.81 (2H, q, J = 16.6 Hz), 1.68 (3H, s), 1.66 (3H, s), 1.53 (4H, m), 1.41 (3H, m), 1.07 (1H, m); ^{13}C{^{1}H} NMR (125 MHz, $CDCl_3$) δ 145.4, 129.0, 128.0, 126.1, 125.3, 125.2, 48.8, 45.7, 42.6, 38.3, 37.0, 35.1, 24.4, 24.1, 19.7, 19.0; HRMS [M + H]$^+$ calculated for $C_{18}H_{25}$ 241.1951, found 241.1964.

*2,3-Dimethyl-5-phenylspiro[5.5]undec-2-ene (**21**). Colorless oil.*

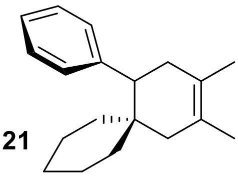

21

^{1}H NMR ($CDCl_3$, 500 MHz) δ 7.24 (2H, m), 7.18 (3H, m), 2.68 (1H, t, J = 5.7 Hz), 2.30 (1H, dd, J = 17.8, 4.0 Hz), 2.20 (1H, dd, J = 17.8, 5.2 Hz), 2.00 (1H, d, J = 17.2 Hz), 1.80 (1H, d, J = 17.8 Hz), 1.69 (3H, s), 1.66 (3H, s), 1.46 (5H, m), 1.33 (1H, m), 1.13 (4H, m); ^{13}C{^{1}H} NMR (125 MHz, $CDCl_3$) δ 144.7, 129.4, 127.9, 126.2, 124.6, 124.6, 49.9, 39.3, 37.0, 35.8, 35.5, 32.5, 26.8, 22.2, 22.1, 19.8, 18.9; HRMS [M + H]$^+$ calculated for $C_{19}H_{27}$ 255.2107, found 255.2116.

*9-(4-Methoxyphenyl)-6,7-dimethyl-2-oxaspiro[3.5]non-6-ene (**22**). Colorless oil.*

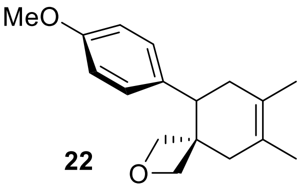

22

^{1}H NMR ($CDCl_3$, 500 MHz) δ 7.11 (2H, d, J = 8.6 Hz), 6.82 (2H, d, J = 8.6 Hz), 4.69 (1H, d, J = 5.7 Hz), 4.47 (1H, d, J = 5.7 Hz), 4.31 (1H, d, J = 5.7 Hz), 4.11 (1H, d, J = 5.7 Hz), 3.78 (3H, s), 3.23 (1H, dt, J = 3.2 2.9 Hz), 2.42 (1H, m), 2.33 (1H, d, J = 17.2 Hz), 2.18 (1H, d, J = 19.5 Hz), 2.14 (1H, d, J = 21.2 Hz), 1.69 (3H, s), 1.67 (3H, s); $^{13}C\{^{1}H\}$ NMR (125 MHz, $CDCl_3$) δ 158.5, 135.1, 129.2, 125.8, 124.1, 113.9, 83.2, 80.2, 55.4, 44.6, 42.8, 39.9, 35.0, 19.5, 19.0; HRMS $[M + H]^+$ calculated for $C_{17}H_{23}O_2$ 259.1693, found 259.1670.

*11-(4-Methoxyphenyl)-8,9-dimethyl-3-oxaspiro[5.5]undec-8-ene (**23**). Colorless oil.*

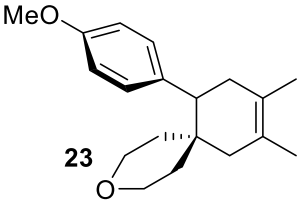

23

^{1}H NMR ($CDCl_3$, 500 MHz) δ 7.07 (2H, d, J = 8.6 Hz), 6.80 (2H, d, J = 8.6 Hz), 3.78 (3H, s), 3.72 (2H, m), 3.61 (1H, dt, J = 11.5, 2.9 Hz), 3.52 (1H, dt, J = 11.5, 2.9 Hz), 2.64 (1H, t, J = 5.7 Hz), 2.36 (1H, dd, J = 17.8, 5.2 Hz), 2.15 (1H, m), 1.99 (2H, m), 1.70 (3H, s), 1.67 (3H, s), 1.49 (2H, m), 1.43 (1H, ddt, J = 14.3, 2.9, 2.3 Hz), 1.04 (1H, ddt, J = 13.2, 2.9, 2.3 Hz); $^{13}C\{^{1}H\}$ NMR (125 MHz, $CDCl_3$) δ 158.3, 135.8, 130.1, 125.0, 124.1, 113.5, 64.1, 55.5, 48.9, 37.7, 36.5, 35.4, 33.9, 33.5, 30.0, 19.8, 18.9; HRMS $[M + H]^+$ calculated for $C_{19}H_{27}O_2$ 287.2006, found 287.2034.

*tert-Butyl 9-(4-methoxyphenyl)-6,7-dimethyl-2-azaspiro[3.5]non-6-ene-2-carboxylate (**24**). Yellow oil.*

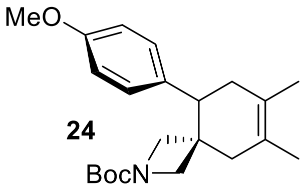

24

^{1}H NMR ($CDCl_3$, 500 MHz) δ 7.11 (2H, d, J = 8.6 Hz), 6.82 (2H, d, J = 8.6 Hz), 3.88 (1H, d, J = 8.6 Hz), 3.78 (3H, s), 3.66 (1H, d, J = 8.0 Hz), 3.52 (1H, d, J = 8.0 Hz), 3.28 (1H, d, J = 8.6 Hz), 2.99 (1H, dt, J = 4.0, 2.3 Hz), 2.43 (1H, dd, J = 16.6, 3.4 Hz), 2.15 (3H, m), 1.67 (6H, s), 1.41 (9H, s); ^{13}C{^{1}H} NMR (125 MHz, $CDCl_3$) δ 158.5, 156.9, 134.9, 129.4, 125.9, 124.1, 113.9, 79.4, 55.5, 45.0, 40.7, 37.5, 35.3, 28.7, 19.5, 19.0; HRMS [M + H]$^+$ calculated for $C_{22}H_{32}NO_3$ 358.2377, found 385.2348.

*tert-Butyl 11-(4-methoxyphenyl)-8,9-dimethyl-3-azaspiro[5.5]undec-8-ene-3-carboxylate (**25**). White solid.*

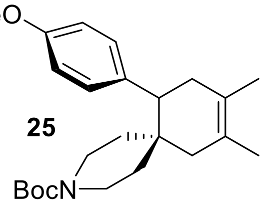

25

^{1}H NMR ($CDCl_3$, 500 MHz) δ 7.06 (2H, d, J = 8.6 Hz), 6.80 (2H, d, J = 8.6 Hz), 3.79 (3H, s), 3.72 (2H, m), 3.01 (1H, dt, J = 13.8, 2.3 Hz), 2.91 (1H, t, J = 9.7 Hz), 2.63 (1H, t, J = 5.7 Hz), 2.33 (1H, d, J = 16.0 Hz), 2.16 (1H, d, J = 16.6 Hz), 1.98 (1H, d, J = 16.6 Hz), 1.87 (1H, d, J = 17.2 Hz), 1.69 (3H, s), 1.67 (3H, s), 1.42 (9H, s), 1.32 (3H, m), 1.13 (1H, m); ^{13}C{^{1}H} NMR (125 MHz, $CDCl_3$) δ 158.3, 155.3, 130.1, 125.0, 124.0, 113.5, 79.4, 55.5, 48.5, 37.6, 35.7, 34.2, 30.0, 28.7, 19.8, 18.9; HRMS [M + H]$^+$ calculated for $C_{24}H_{36}NO_3$ 386.2690, found 386.2668.

(1) D. Toryn; S. Grebies; D. Mowpriya; N. Maximilian; S. L. Malte; G. Christian; R. J. Bart; G. Frank, *Angew. Chem. Int., Ed.* **2021**, *60*, 8537–8541.

(2) P. R. D. Murray; W. M. M. Bussink; G. H. M. Davies, F. W. Mei; A. H. Antropow; J. T. Edwards; L. A. D'Agostino; J. M. Ellis; L. G. Hamann; F. R.-Michailidis; R. R. Knowles, *J. Am. Chem. Soc.* **2021**, *143*, 4055−4063.

(3) K. A. Bahou; D. C. Braddock; A. G. Meyer; G. P. Savage; Z. Shi; T. He, *J. Org. Chem.* **2020**, *85*, 4906–4917.

(4) J. Li; H. Tian; M. Jiang; H. Yang; Y. Zhaoa; H. Fu, *Chem. Commun.* **2016**, *52*, 8862–8864.

(5) G. S. Lee; D. Kim; S. H. Hong, *Nat. Commun.* **2021**, *12*, 991.

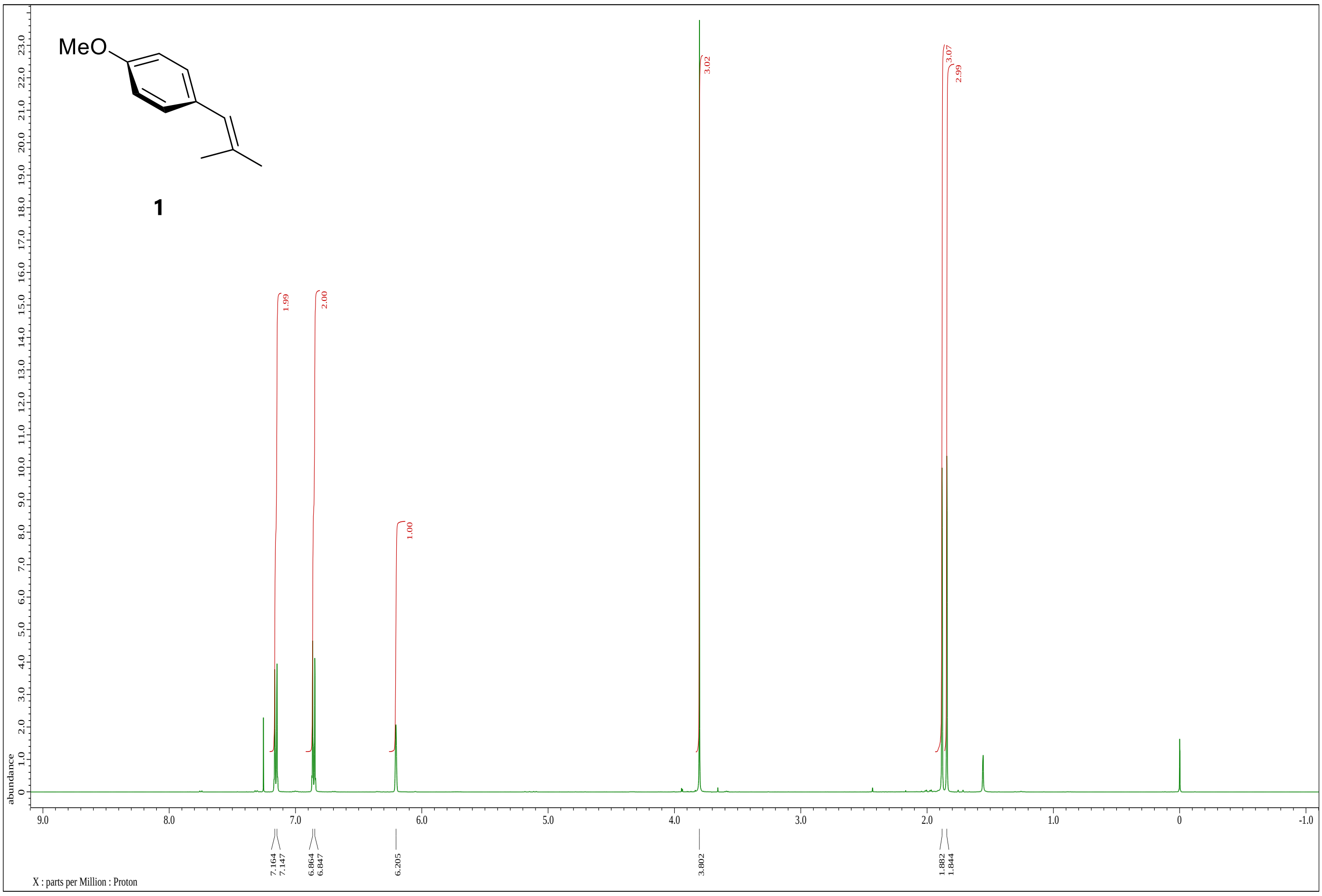
MeO
1
abundance
3.02
3.07
2.99
1.99
2.00
1.00
9.0
8.0
7.0
6.0
5.0
4.0
3.0
2.0
1.0
0
-1.0
7.164
7.147
6.864
6.847
6.205
3.802
1.882
1.844
X : parts per Million : Proton

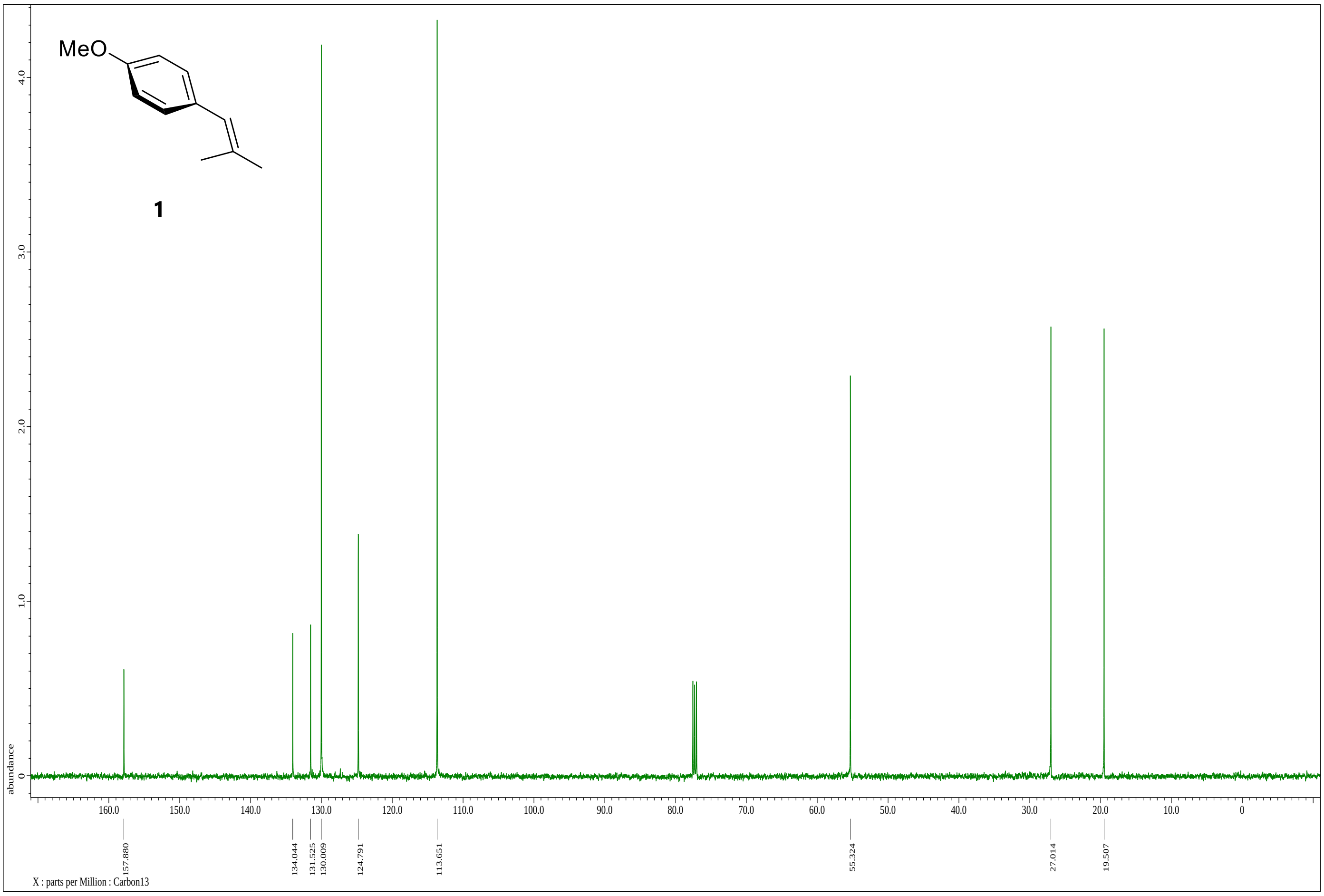
MeO
1
abundance
160.0
150.0
140.0
130.0
120.0
110.0
100.0
90.0
80.0
70.0
60.0
50.0
40.0
30.0
20.0
10.0
0
157.880
134.044
131.525
130.009
124.791
113.651
55.324
27.014
19.507
X : parts per Million : Carbon13

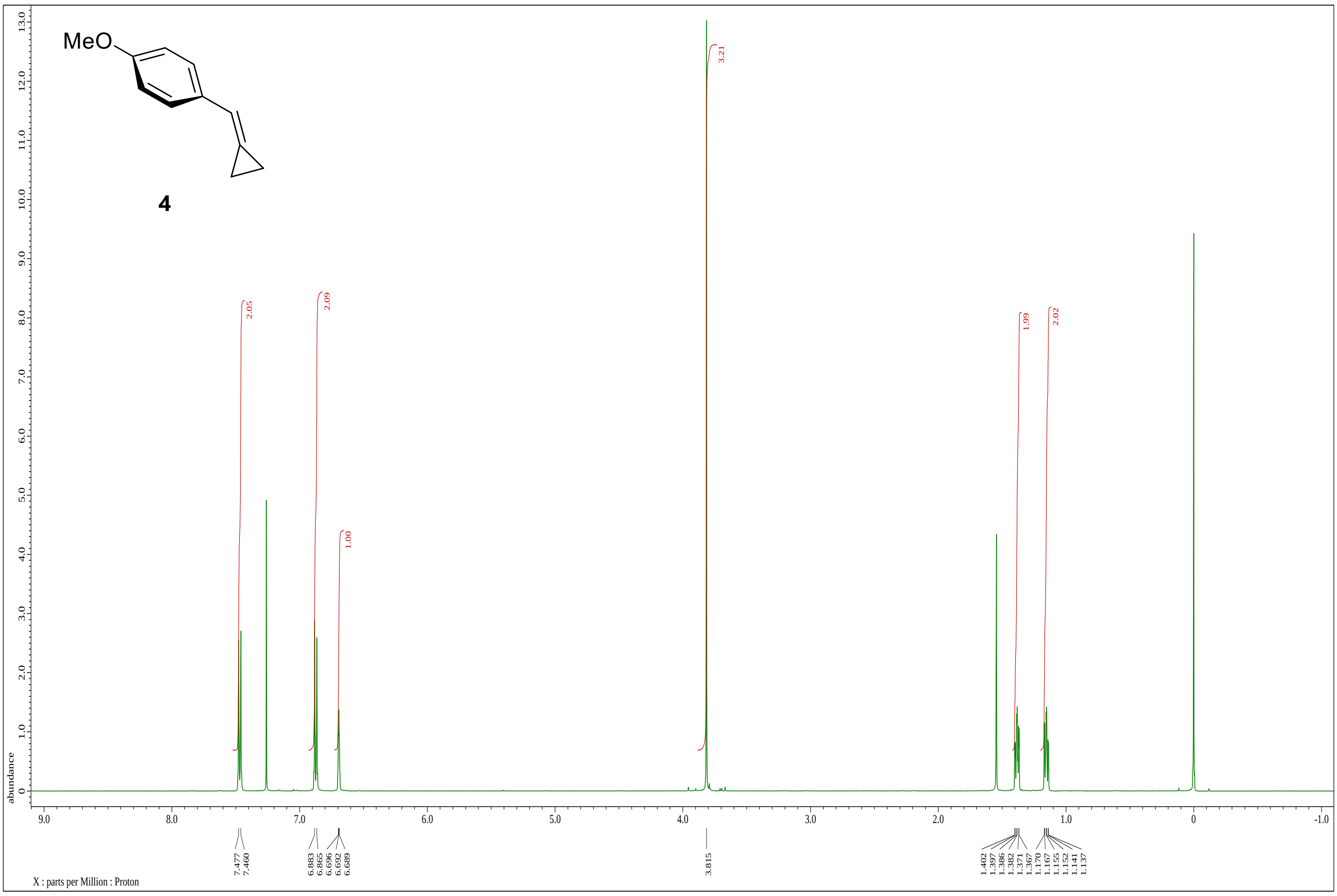
MeO
4
3.21
2.05
2.09
1.00
1.99
2.02
abundance
7.477
7.460
6.883
6.865
6.696
6.692
6.689
3.815
1.402
1.397
1.386
1.382
1.371
1.367
1.170
1.167
1.155
1.152
1.141
1.137
X : parts per Million : Proton

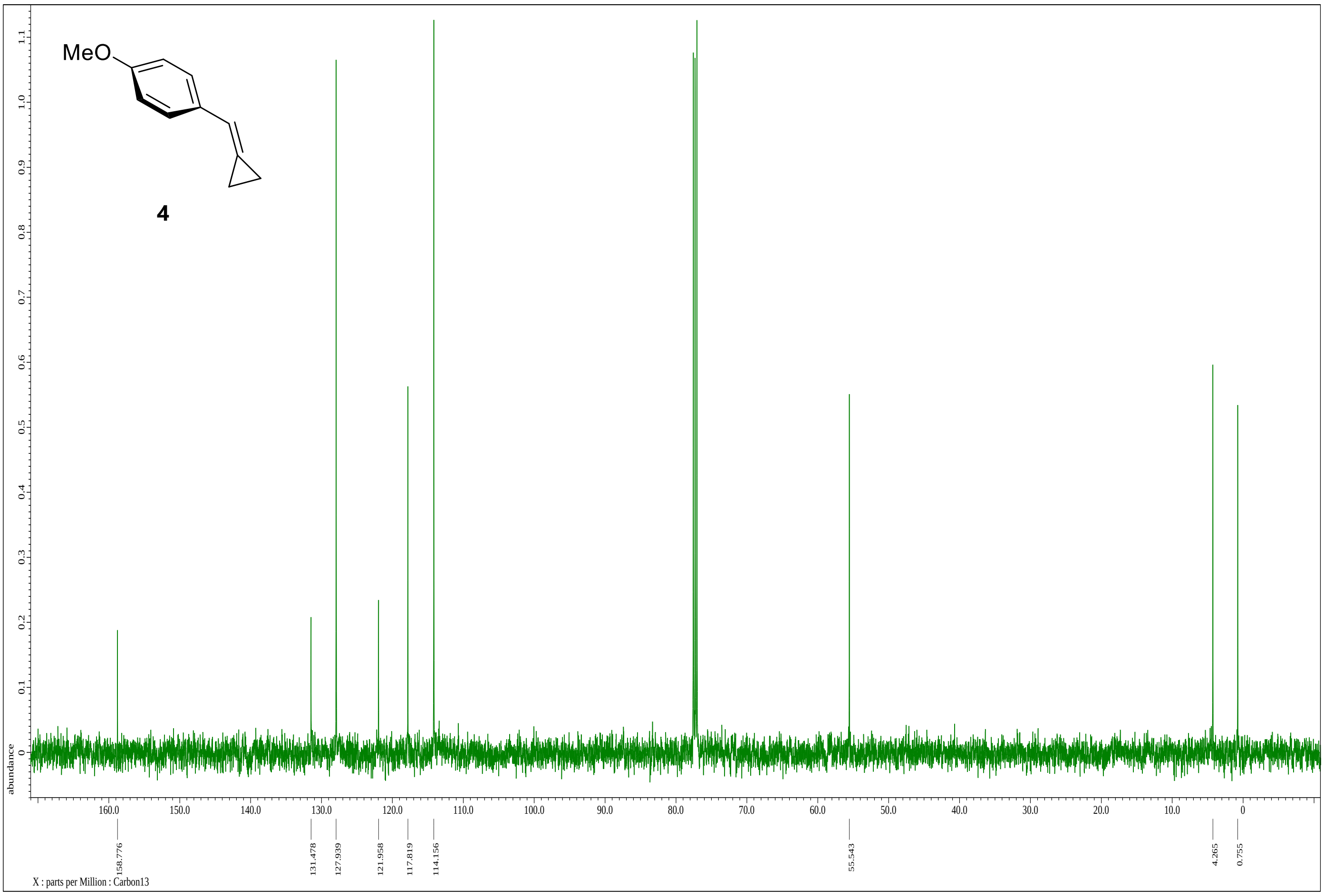
MeO
4
abundance
158.776
131.478
127.939
121.958
117.819
114.156
55.543
4.265
0.755
X : parts per Million : Carbon13

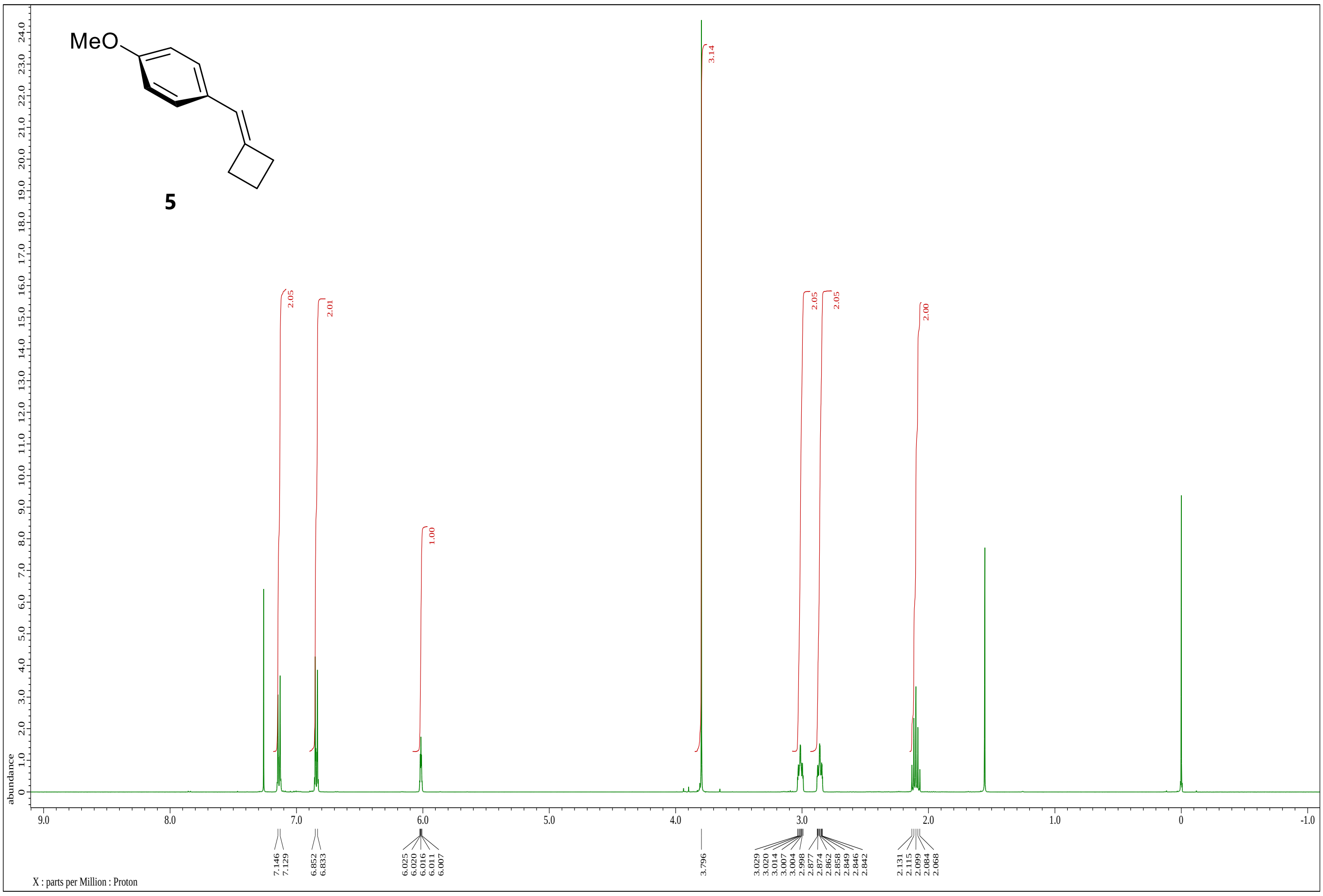
MeO
5
X : parts per Million : Proton

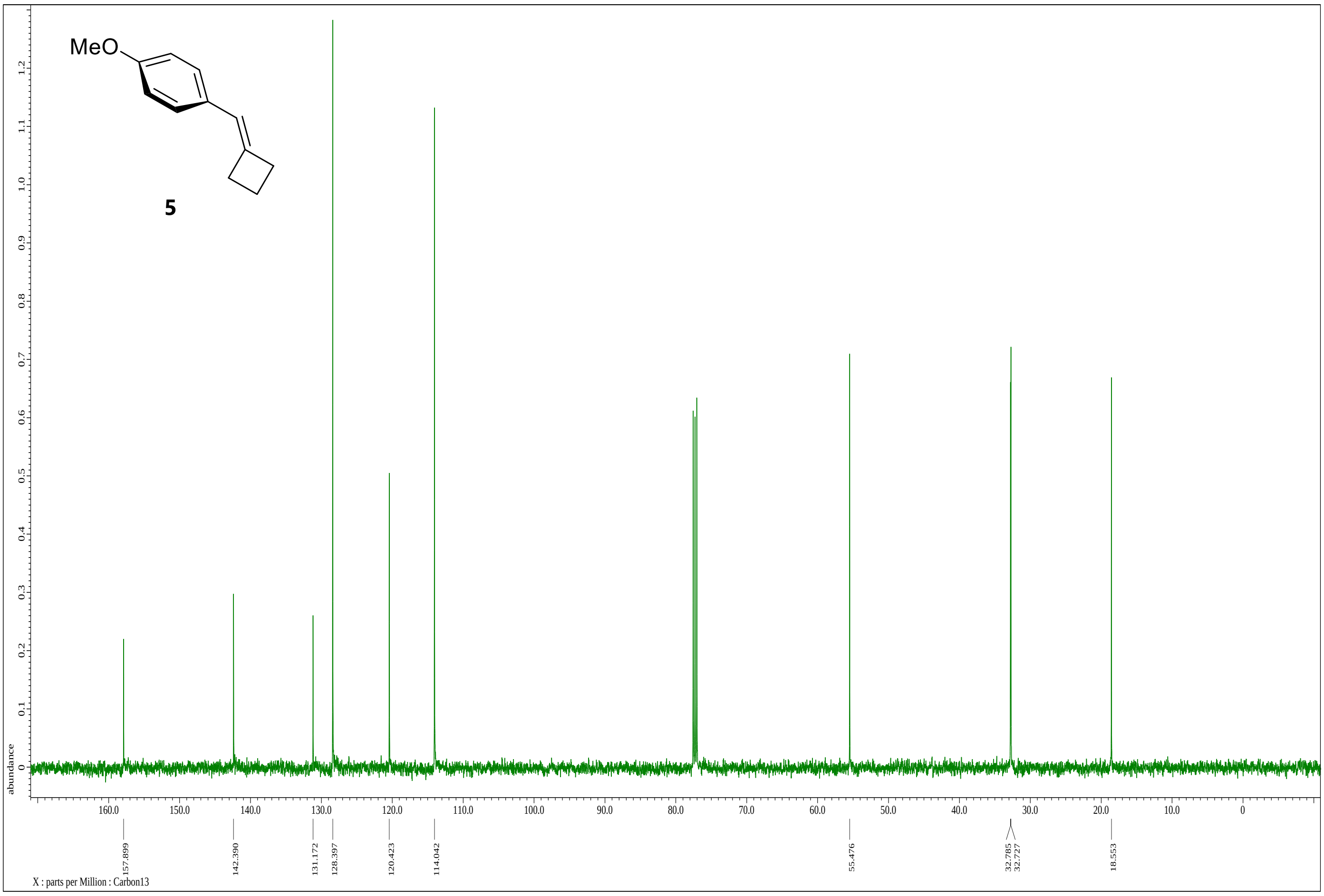
MeO
5
X : parts per Million : Carbon13

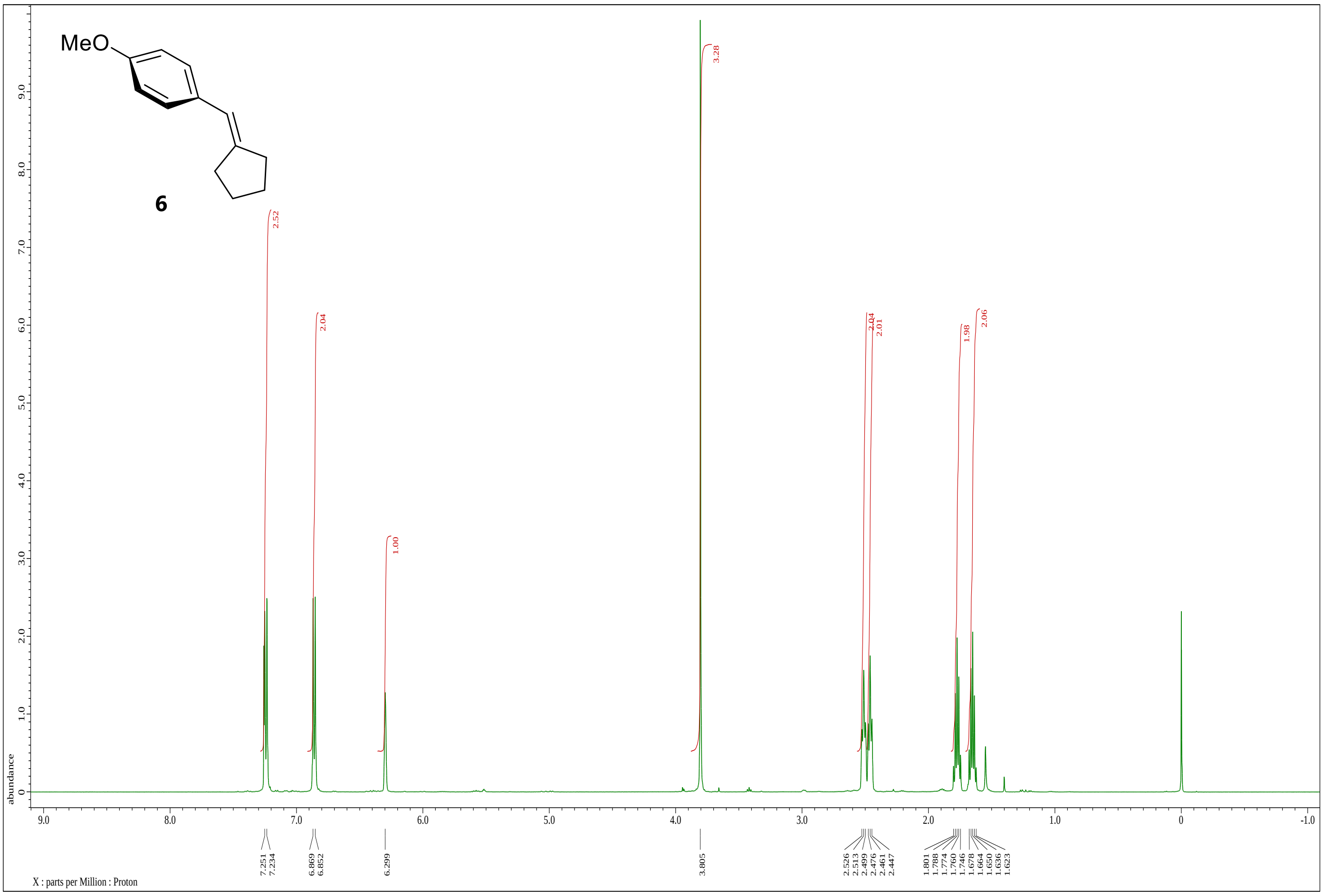
MeO
6
2.52
2.04
1.00
3.28
2.04
2.01
1.98
2.06
7.251
7.234
6.869
6.852
6.299
3.805
2.526
2.513
2.499
2.476
2.461
2.447
1.801
1.788
1.774
1.760
1.746
1.678
1.664
1.650
1.636
1.623
abundance
X : parts per Million : Proton

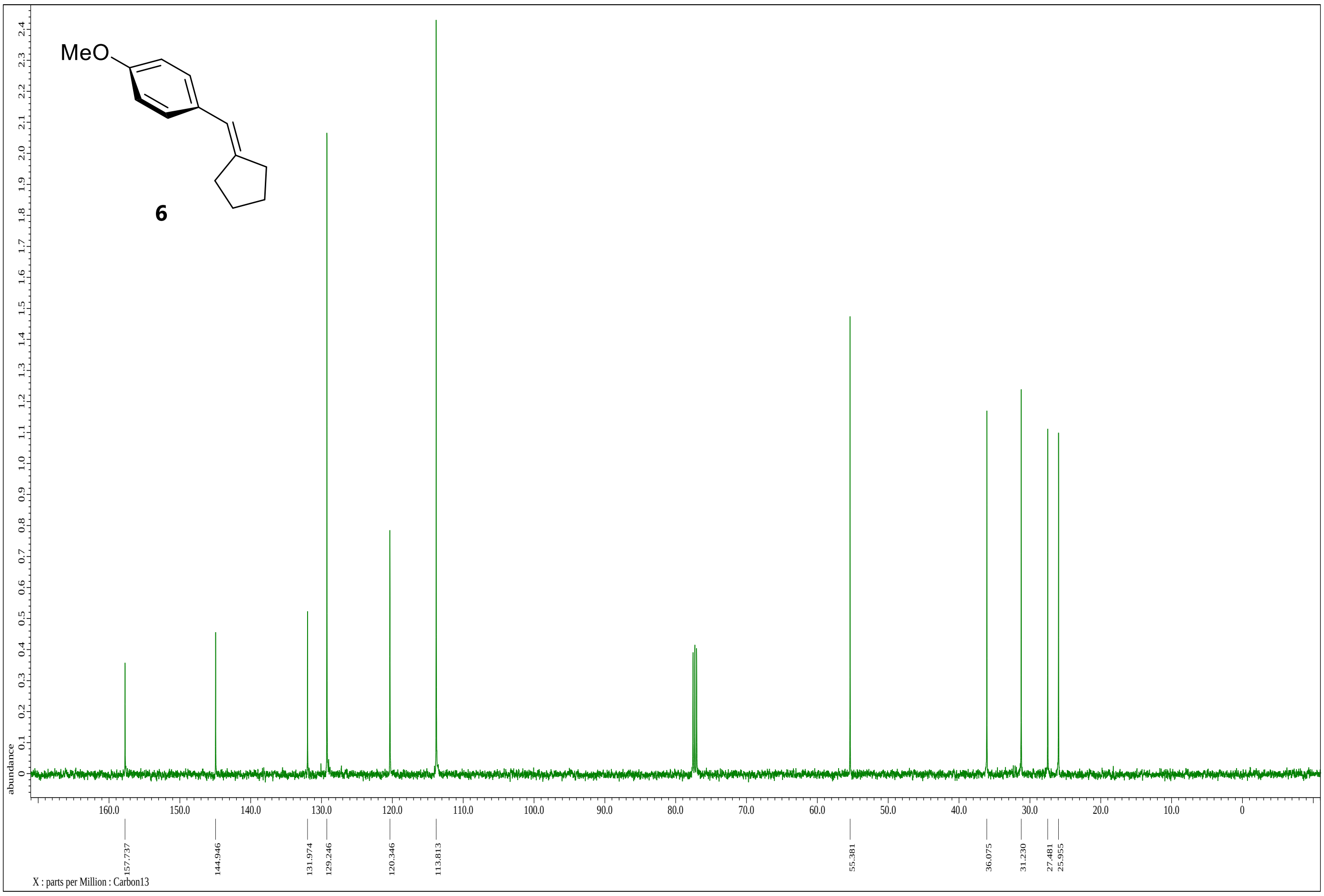
MeO
6
157.737
144.946
131.974
129.246
120.346
113.813
55.381
36.075
31.230
27.481
25.955
abundance
X : parts per Million : Carbon13

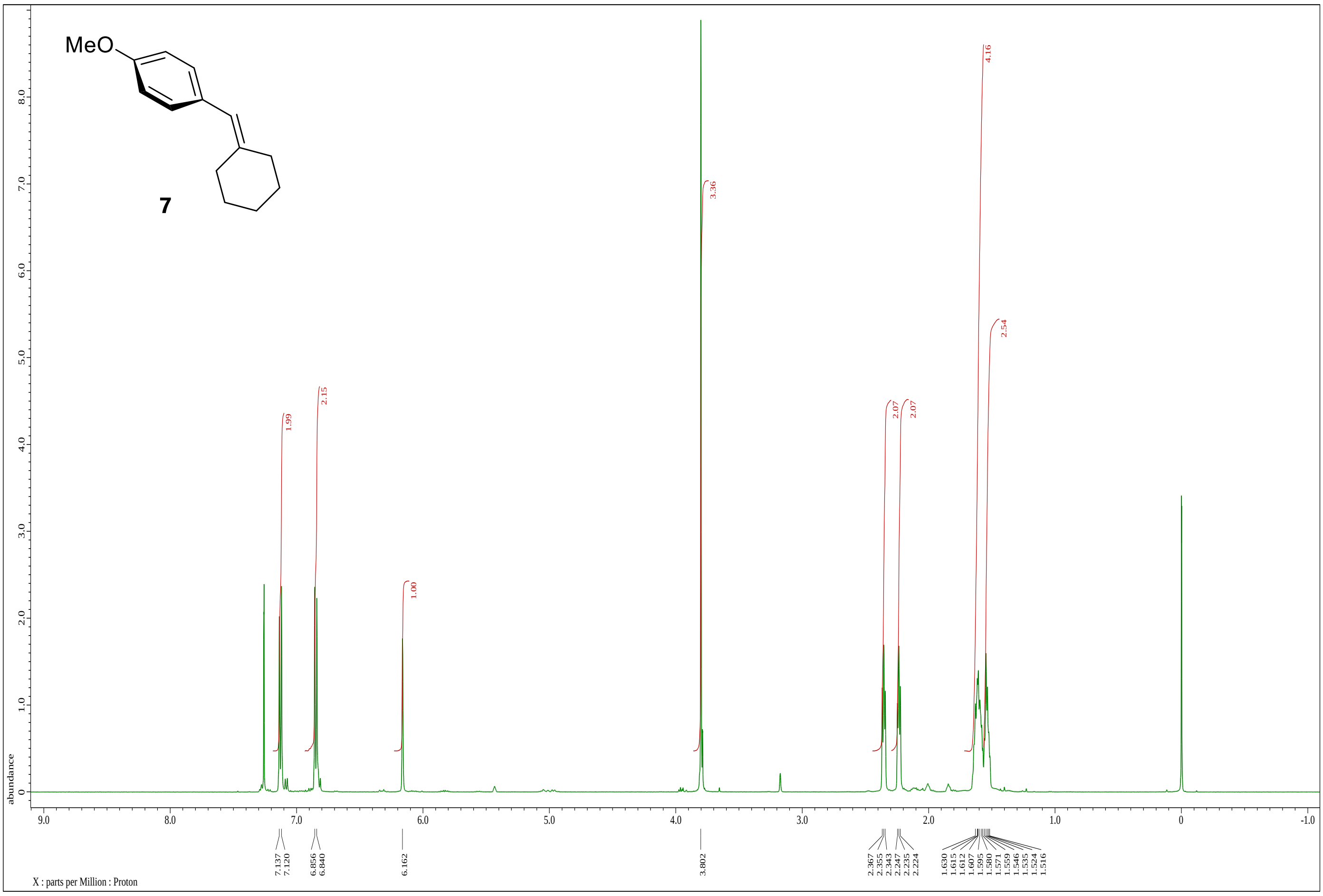
MeO
7
3.36
4.16
2.54
2.15
1.99
2.07
2.07
1.00
7.137
7.120
6.856
6.840
6.162
3.802
2.367
2.355
2.343
2.247
2.235
2.224
1.630
1.615
1.612
1.607
1.595
1.580
1.571
1.559
1.546
1.535
1.524
1.516
abundance
X : parts per Million : Proton

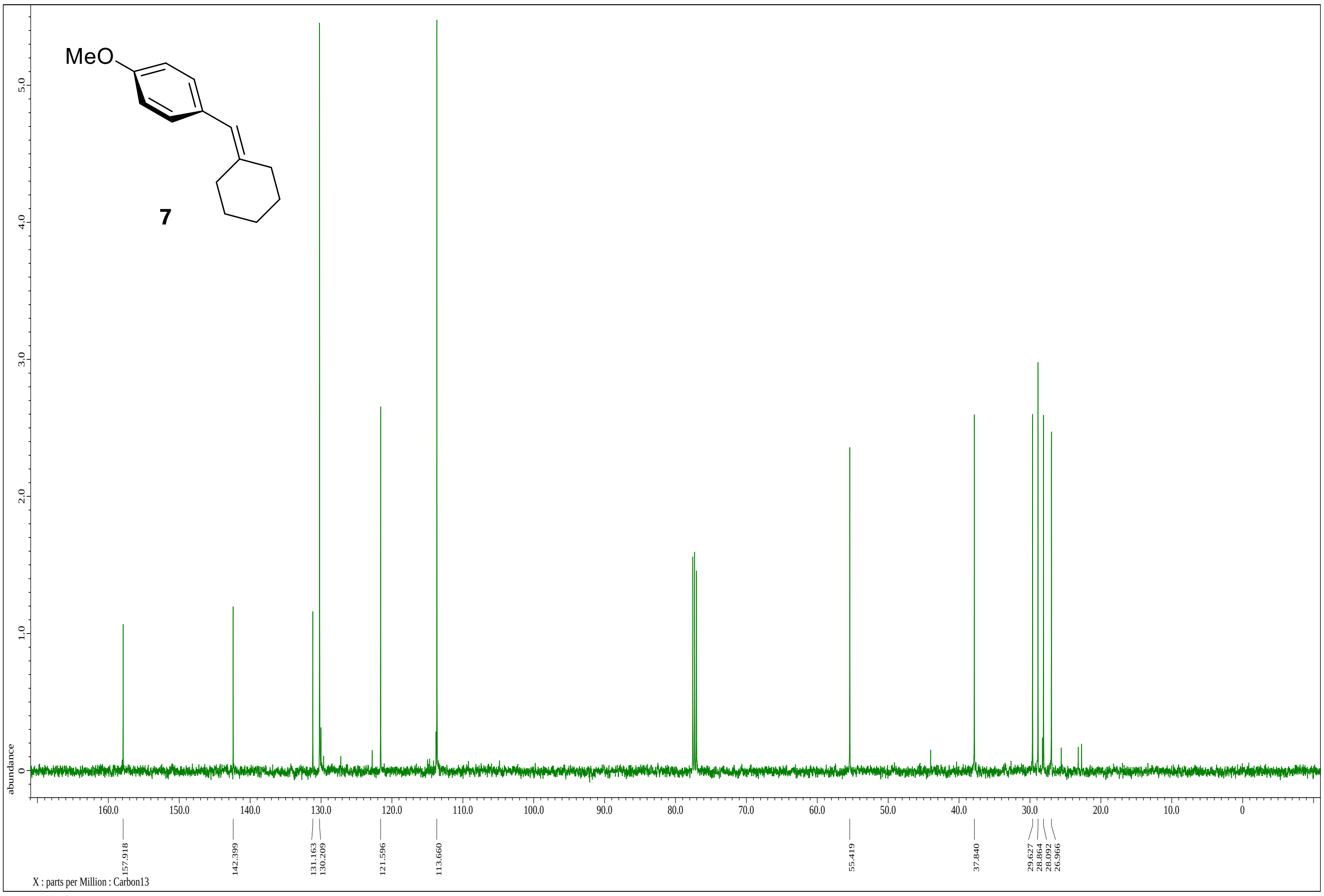
MeO
7
157.918
142.399
131.163
130.209
121.596
113.660
55.419
37.840
29.627
28.864
28.092
26.966
abundance
X : parts per Million : Carbon13

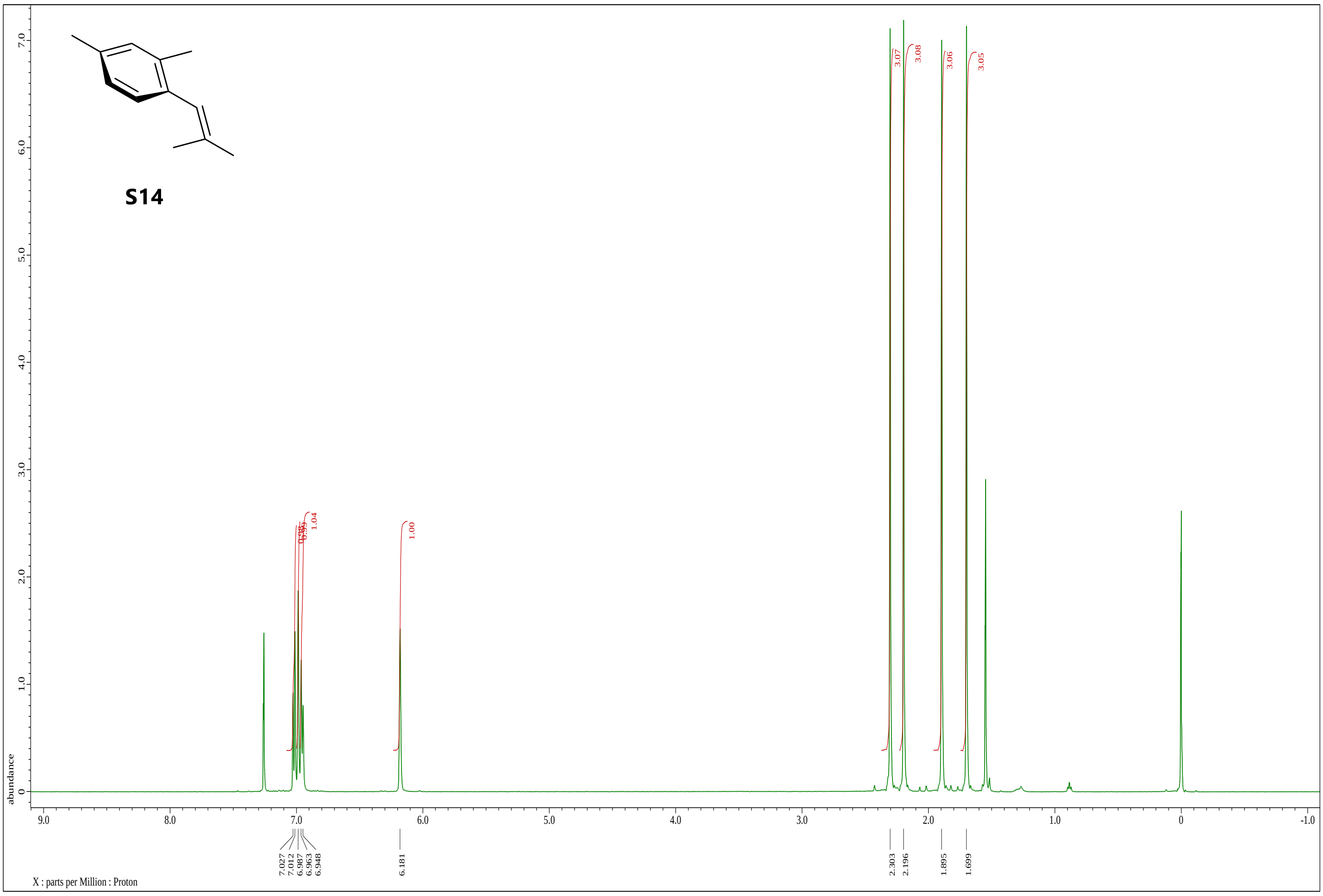
S14
X : parts per Million : Proton
abundance
7.027
7.012
6.987
6.963
6.948
6.181
2.303
2.196
1.895
1.699
1.04
1.00
3.07
3.08
3.06
3.05

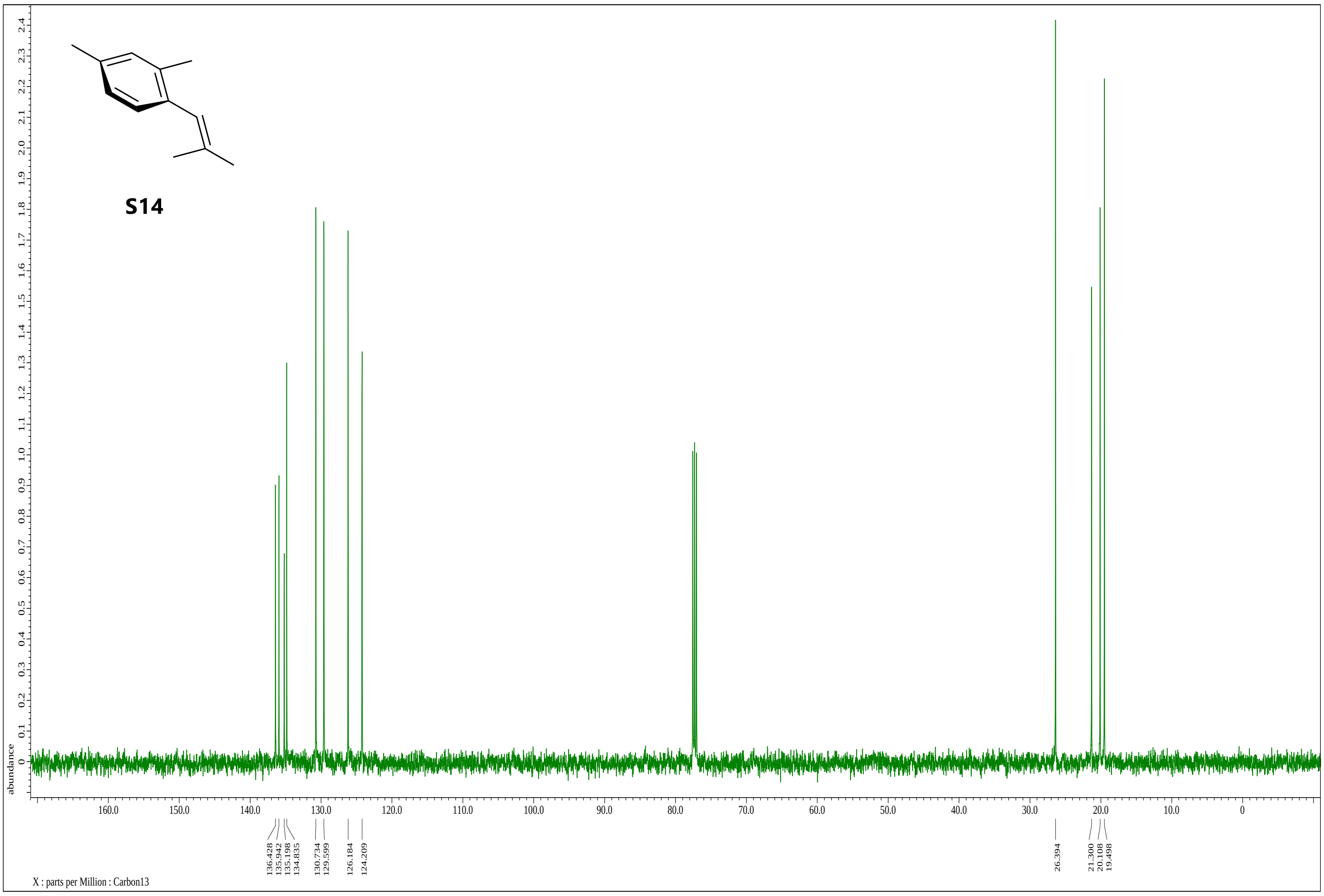
S14
X : parts per Million : Carbon13
abundance
136.428
135.942
135.198
134.835
130.734
129.599
126.184
124.209
26.394
21.300
20.108
19.498

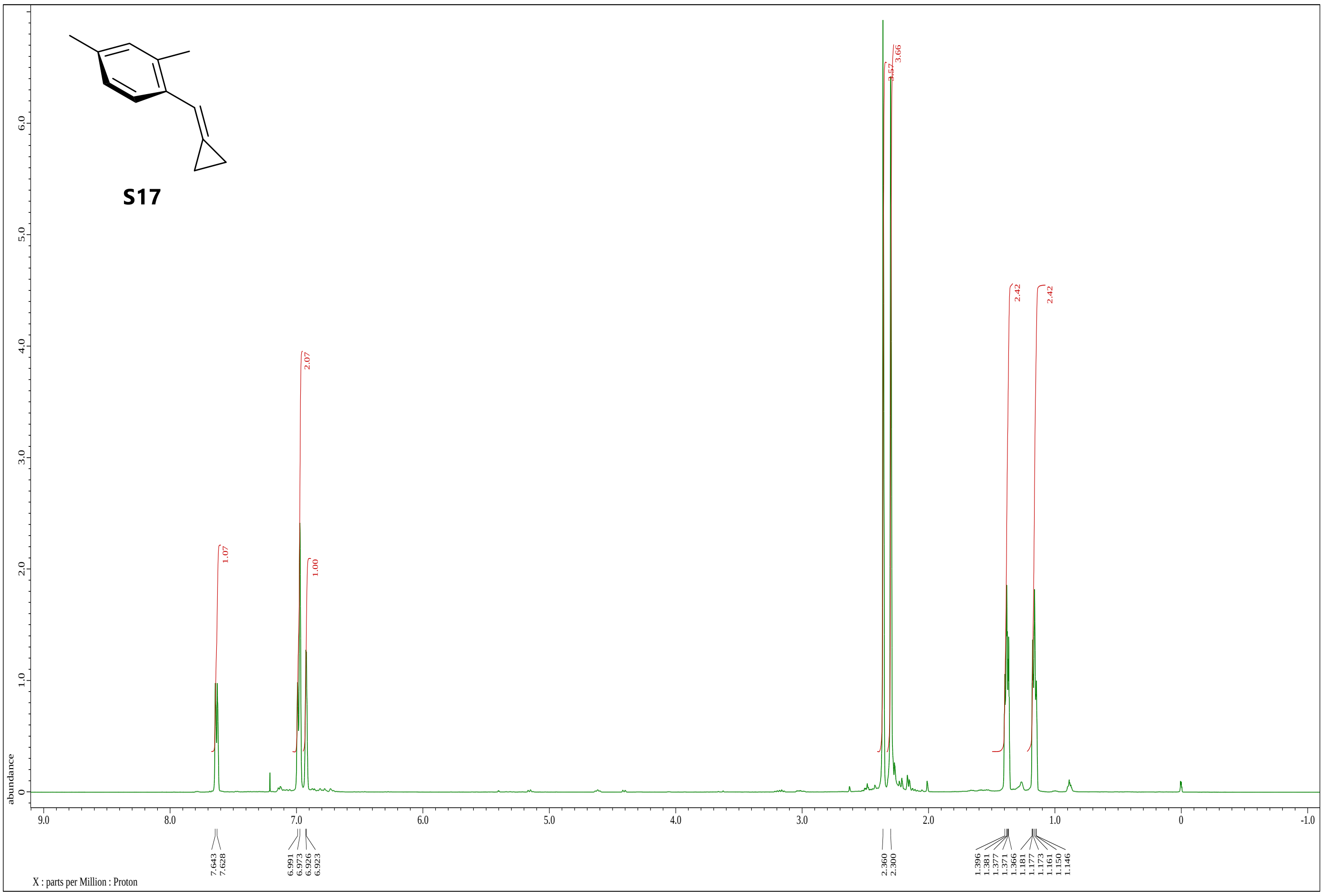
S17
abundance
X : parts per Million : Proton
1.07
2.07
1.00
3.57
3.66
2.42
2.42
7.643
7.628
6.991
6.973
6.926
6.923
2.360
2.300
1.396
1.381
1.377
1.371
1.366
1.181
1.177
1.173
1.161
1.150
1.146

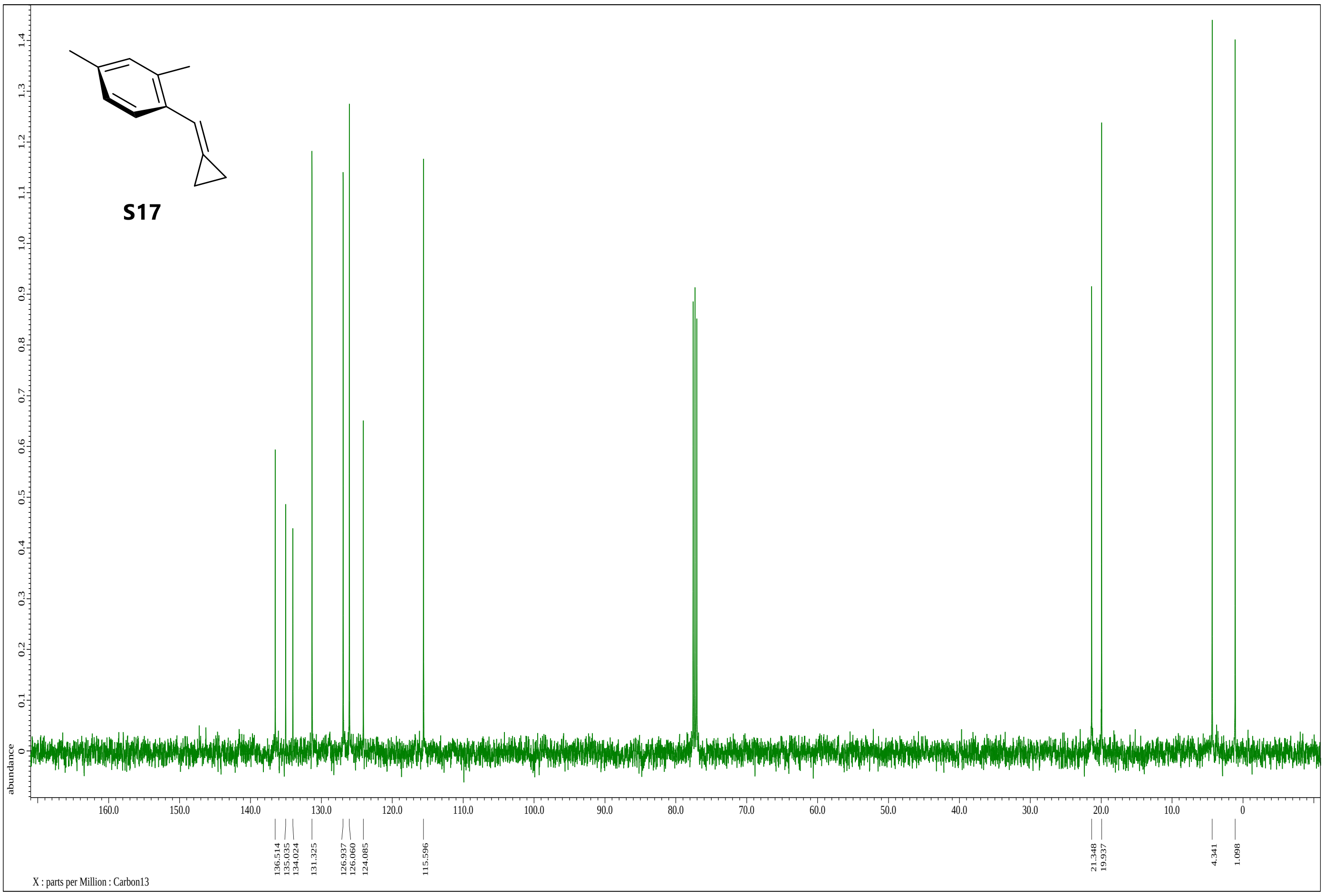
S17
abundance
X : parts per Million : Carbon13
136.514
135.035
134.024
131.325
126.937
126.060
124.085
115.596
21.348
19.937
4.341
1.098

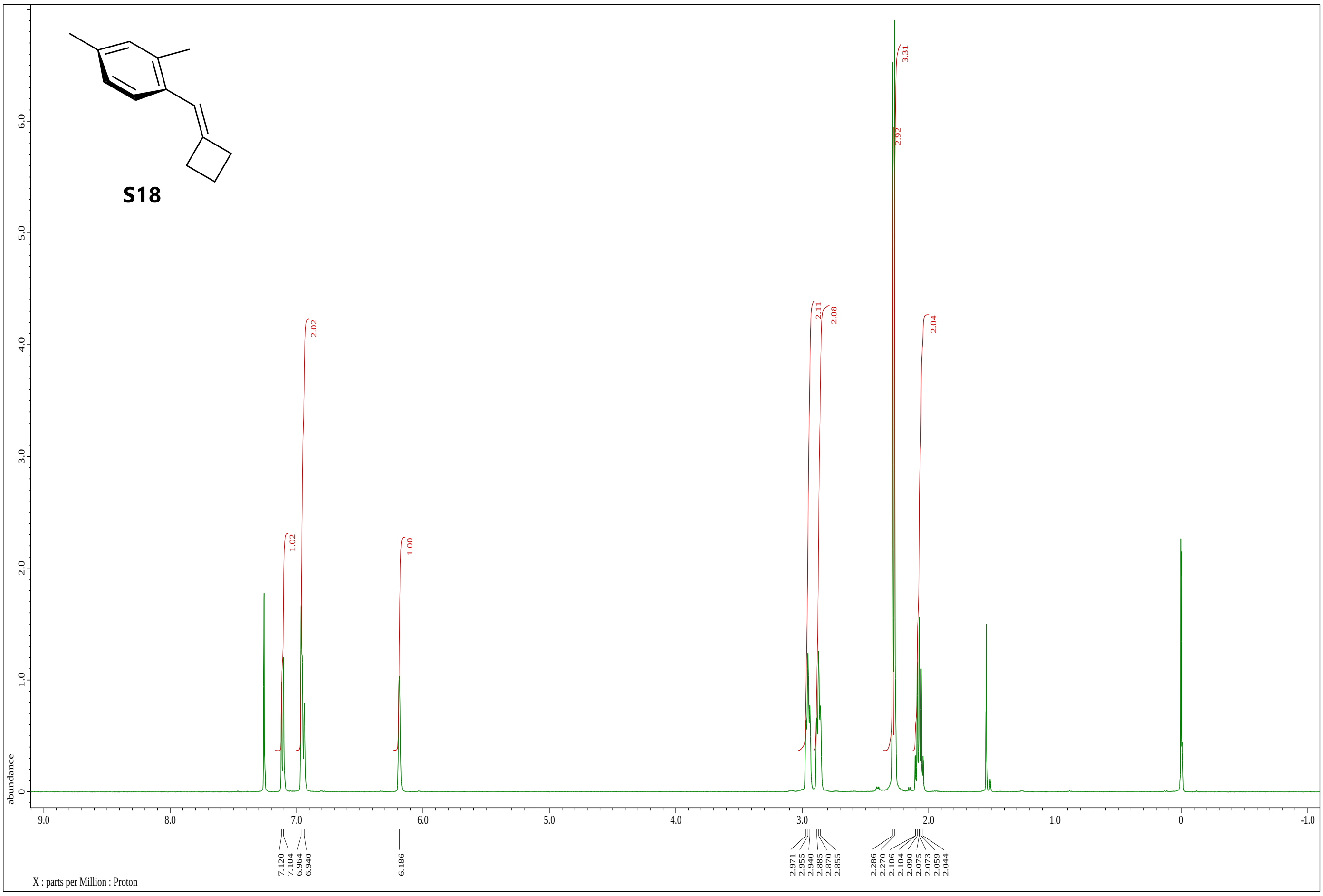
S18
abundance
X : parts per Million : Proton
2.02
1.02
1.00
2.11
2.08
2.04
3.31
2.92
7.120
7.104
6.964
6.940
6.186
2.971
2.955
2.940
2.885
2.870
2.855
2.286
2.270
2.106
2.104
2.090
2.075
2.073
2.059
2.044

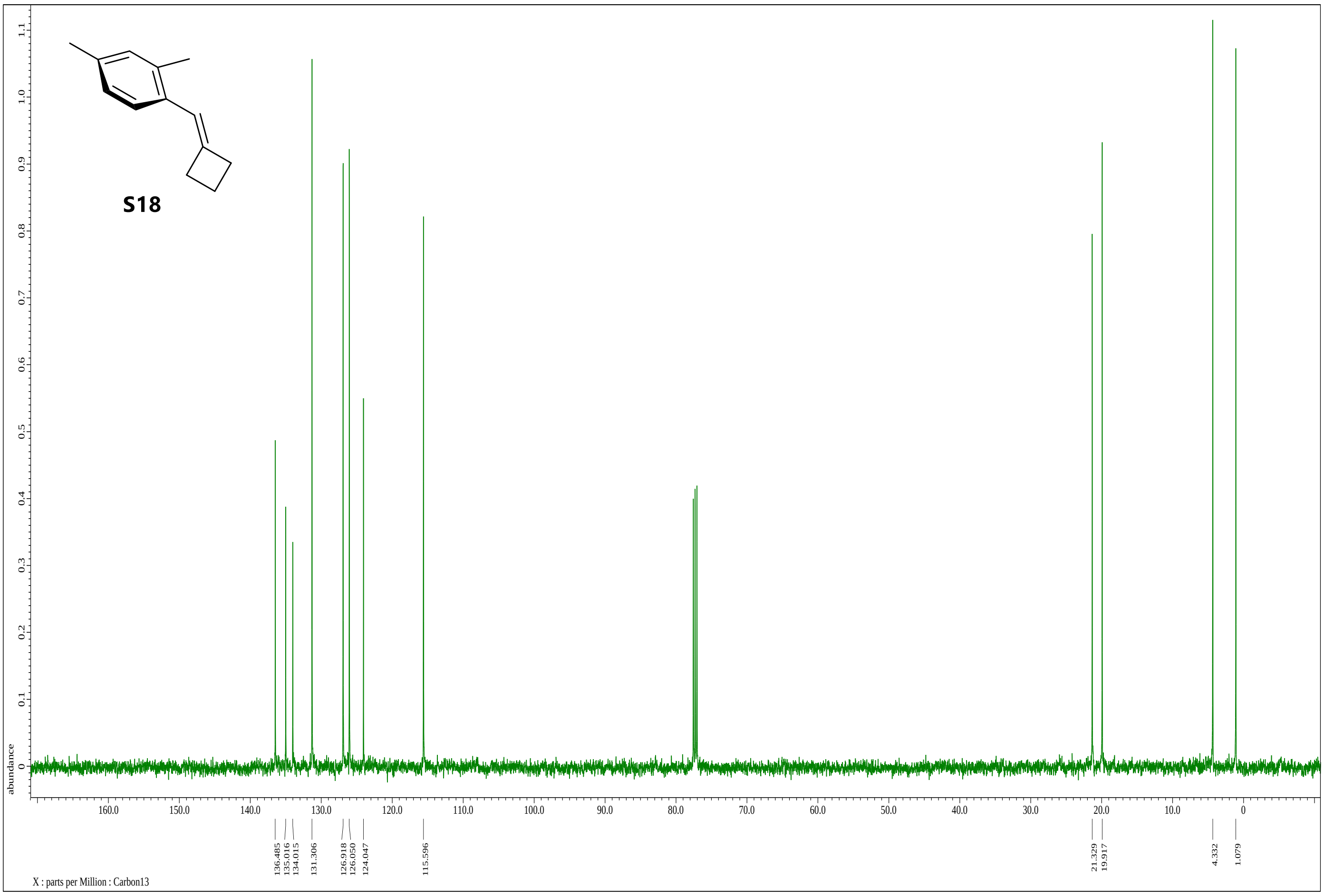
S18
abundance
X : parts per Million : Carbon13
136.485
135.016
134.015
131.306
126.918
126.050
124.047
115.596
21.329
19.917
4.332
1.079

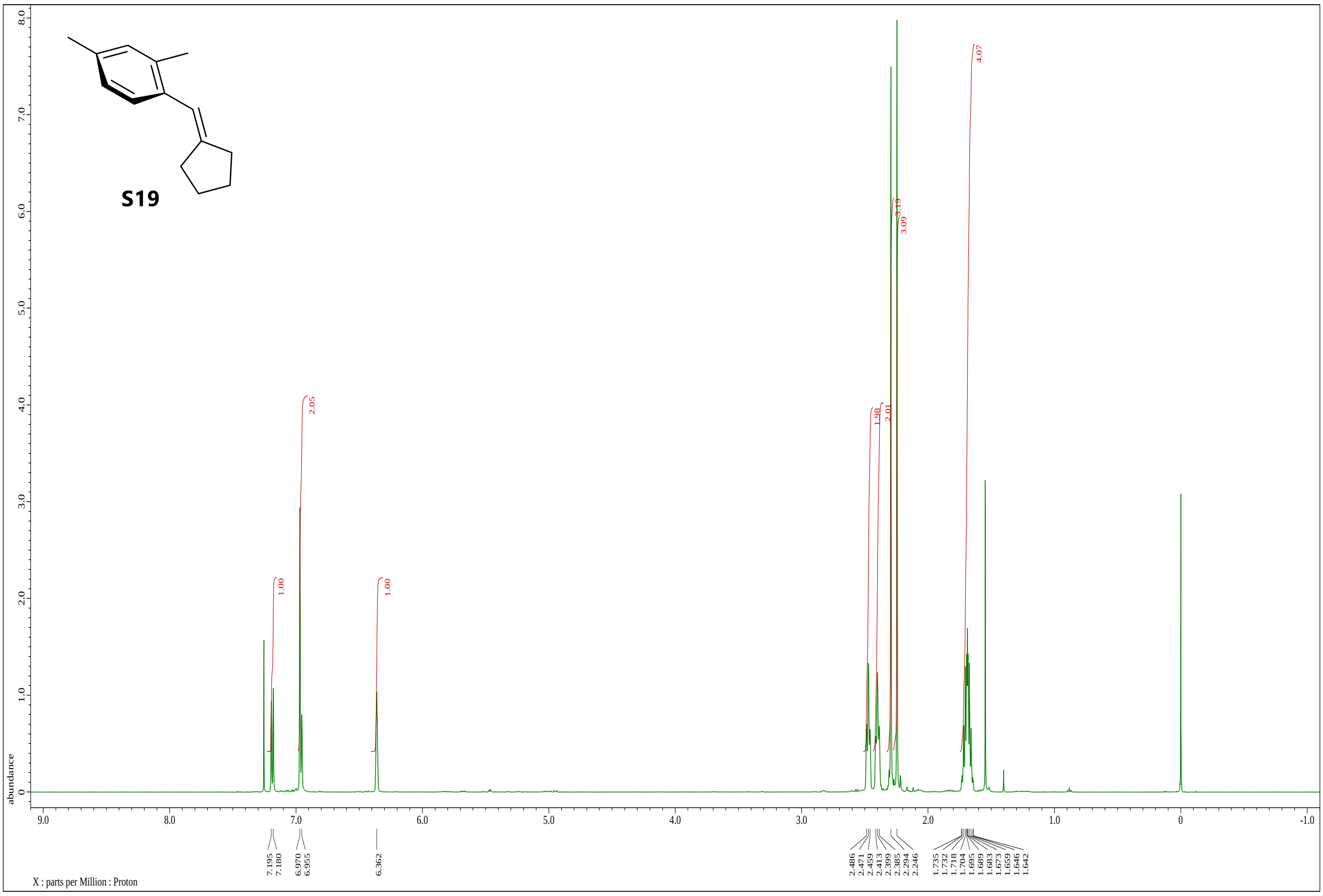
S19
abundance
X : parts per Million : Proton
7.195
7.180
6.970
6.955
6.362
2.486
2.471
2.459
2.413
2.399
2.385
2.294
2.246
1.735
1.732
1.718
1.704
1.695
1.689
1.683
1.673
1.659
1.646
1.642
1.00
2.05
1.00
1.98
2.01
3.13
3.09
4.07

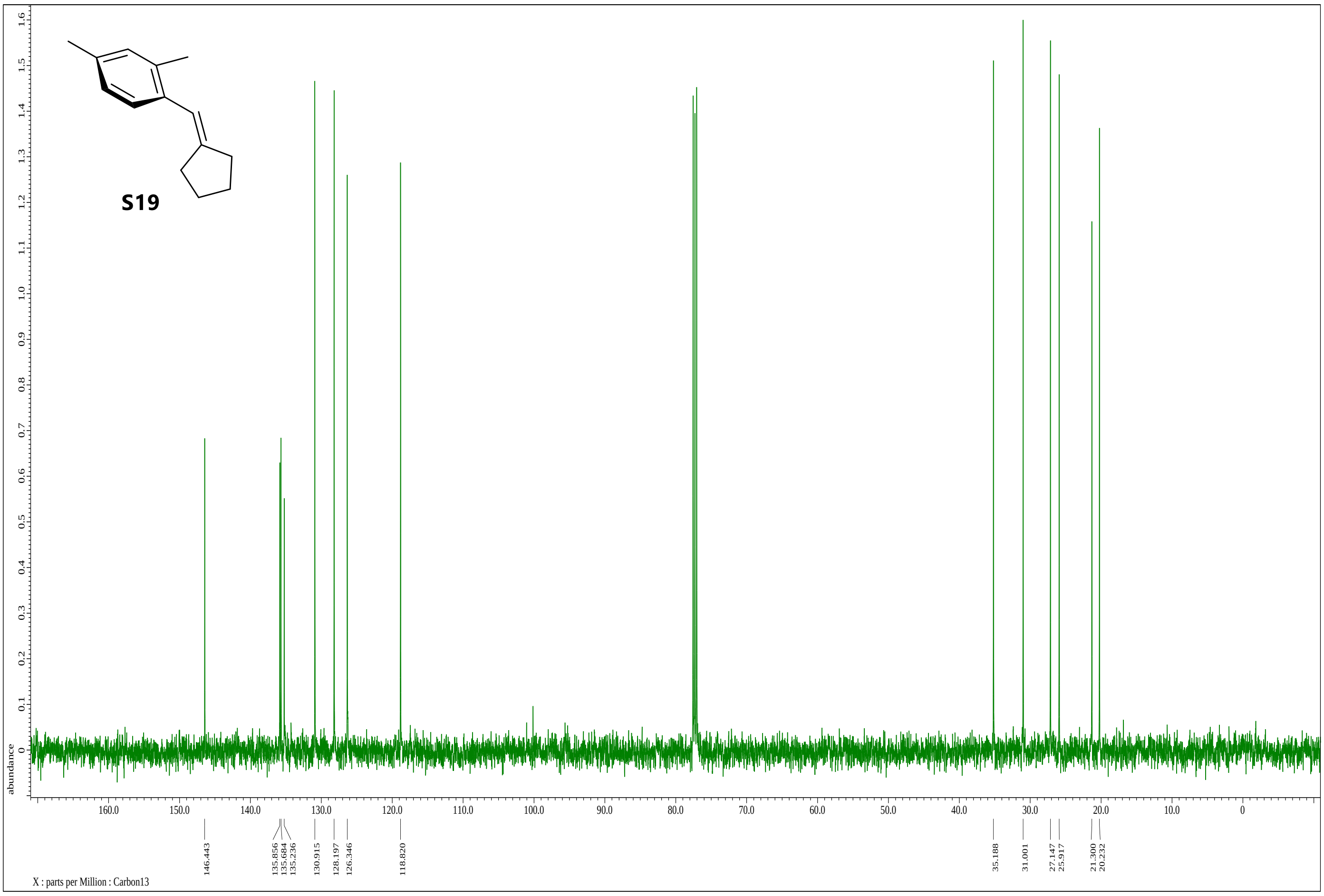
S19
abundance
X : parts per Million : Carbon13
146.443
135.856
135.684
135.236
130.915
128.197
126.346
118.820
35.188
31.001
27.147
25.917
21.300
20.232

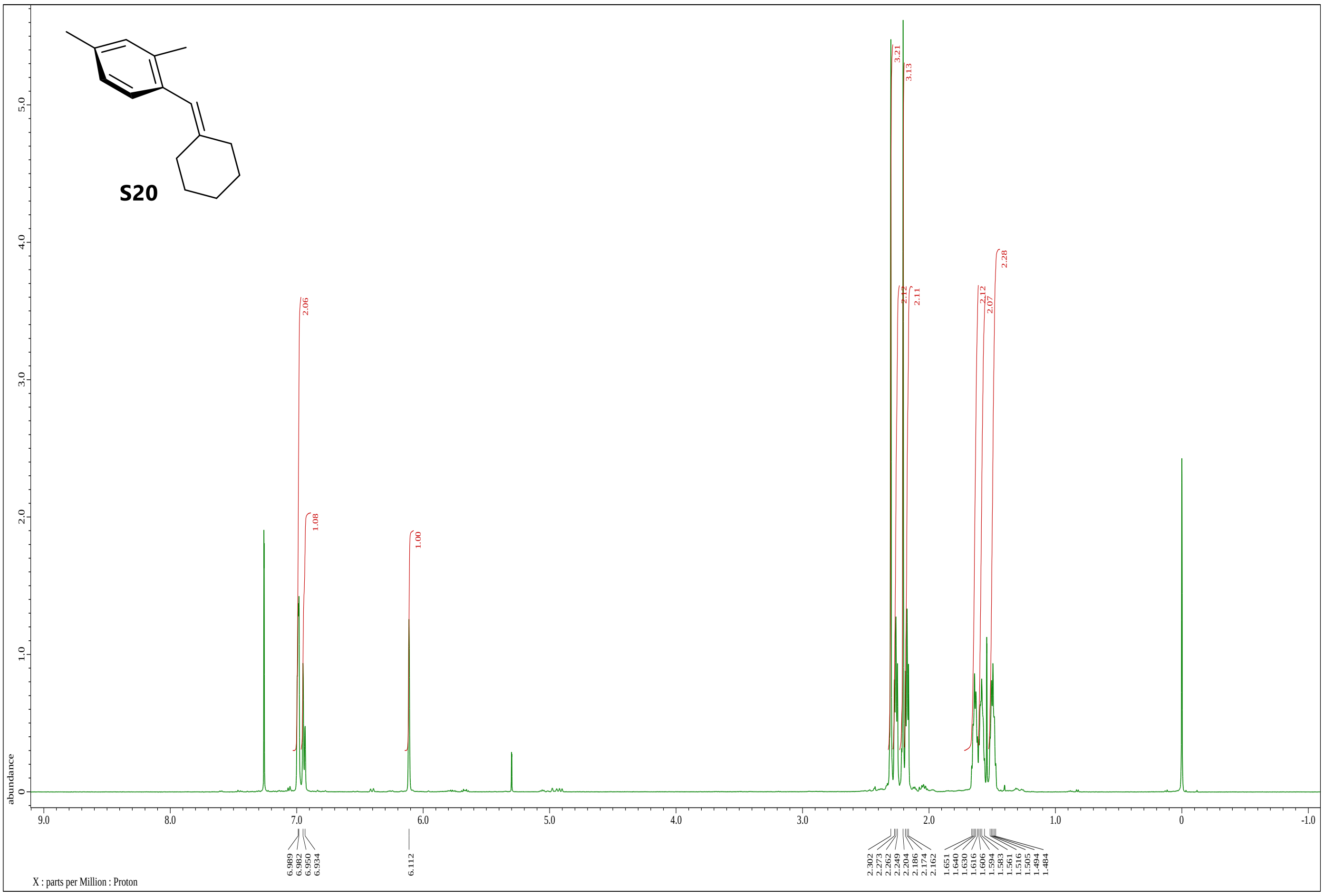
S20
abundance
2.06
1.08
1.00
3.21
3.13
2.12
2.11
2.12
2.07
2.28
6.989
6.982
6.950
6.934
6.112
2.302
2.273
2.262
2.249
2.204
2.186
2.174
2.162
1.651
1.640
1.630
1.616
1.606
1.594
1.583
1.561
1.516
1.505
1.494
1.484
X : parts per Million : Proton

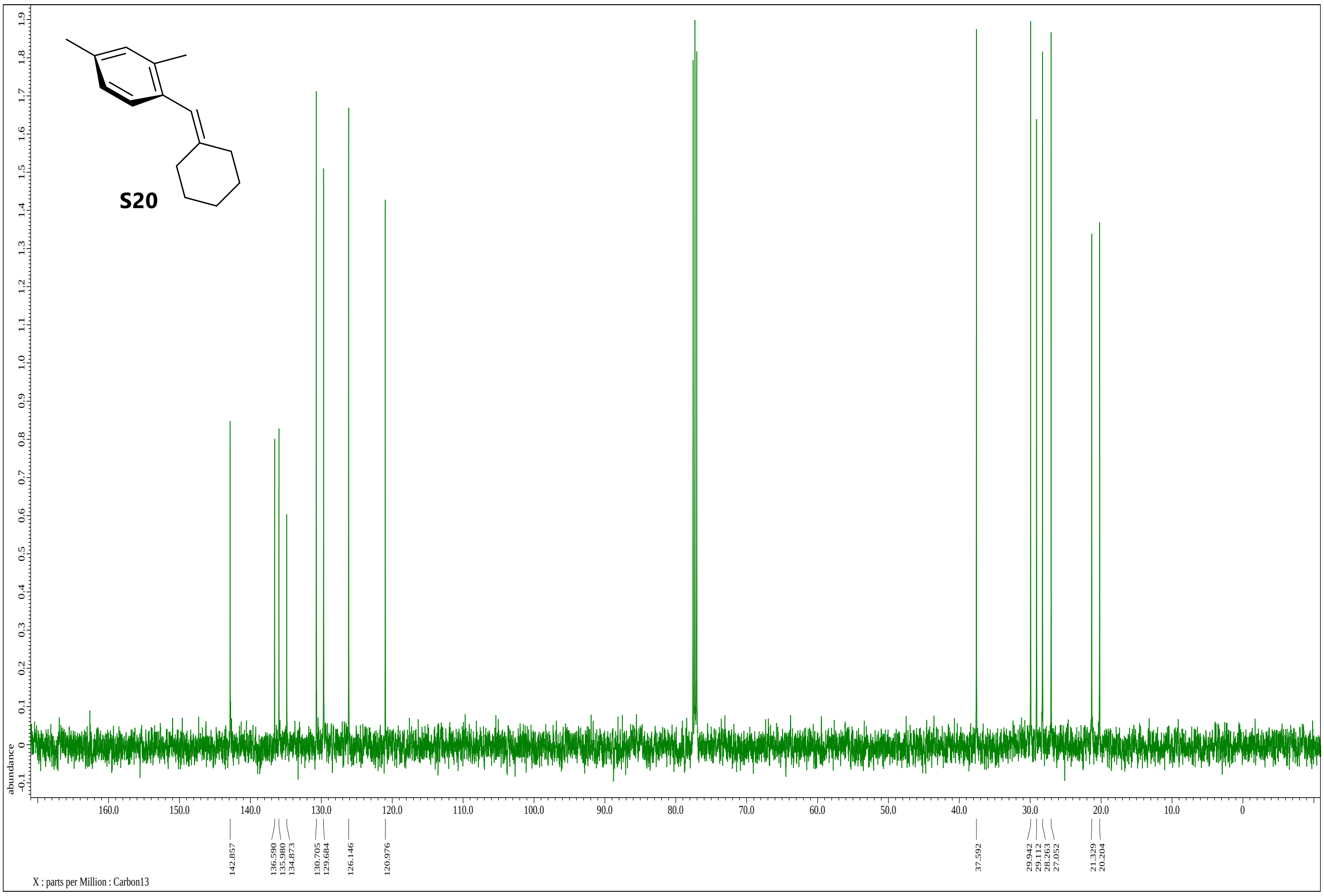
S20
abundance
142.857
136.590
135.980
134.873
130.705
129.684
126.146
120.976
37.592
29.942
29.112
28.263
27.052
21.329
20.204
X : parts per Million : Carbon13

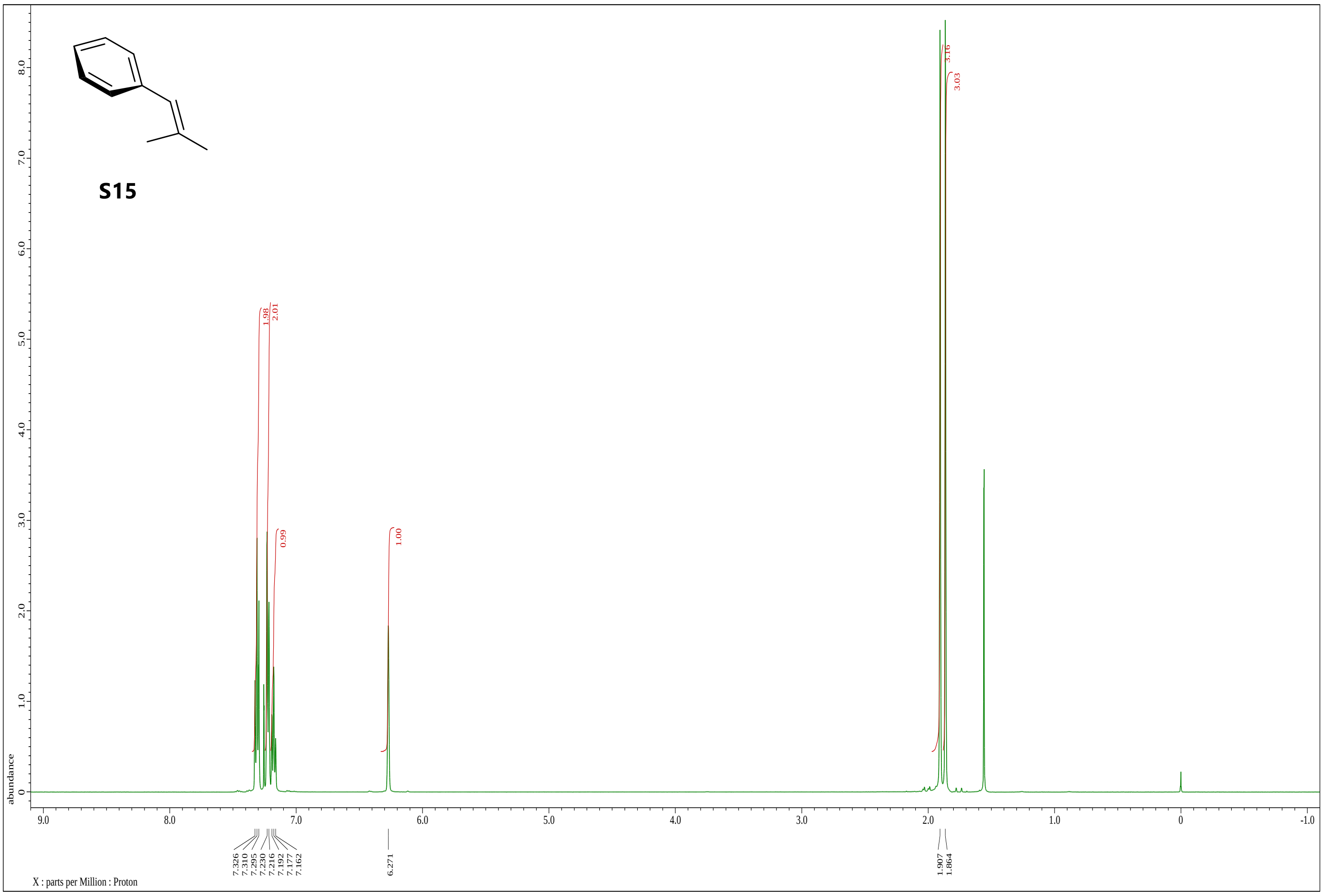

S15
abundance
X : parts per Million : Proton
7.326
7.310
7.295
7.230
7.216
7.192
7.177
7.162
6.271
1.907
1.864
1.98
2.01
0.99
1.00
3.16
3.03

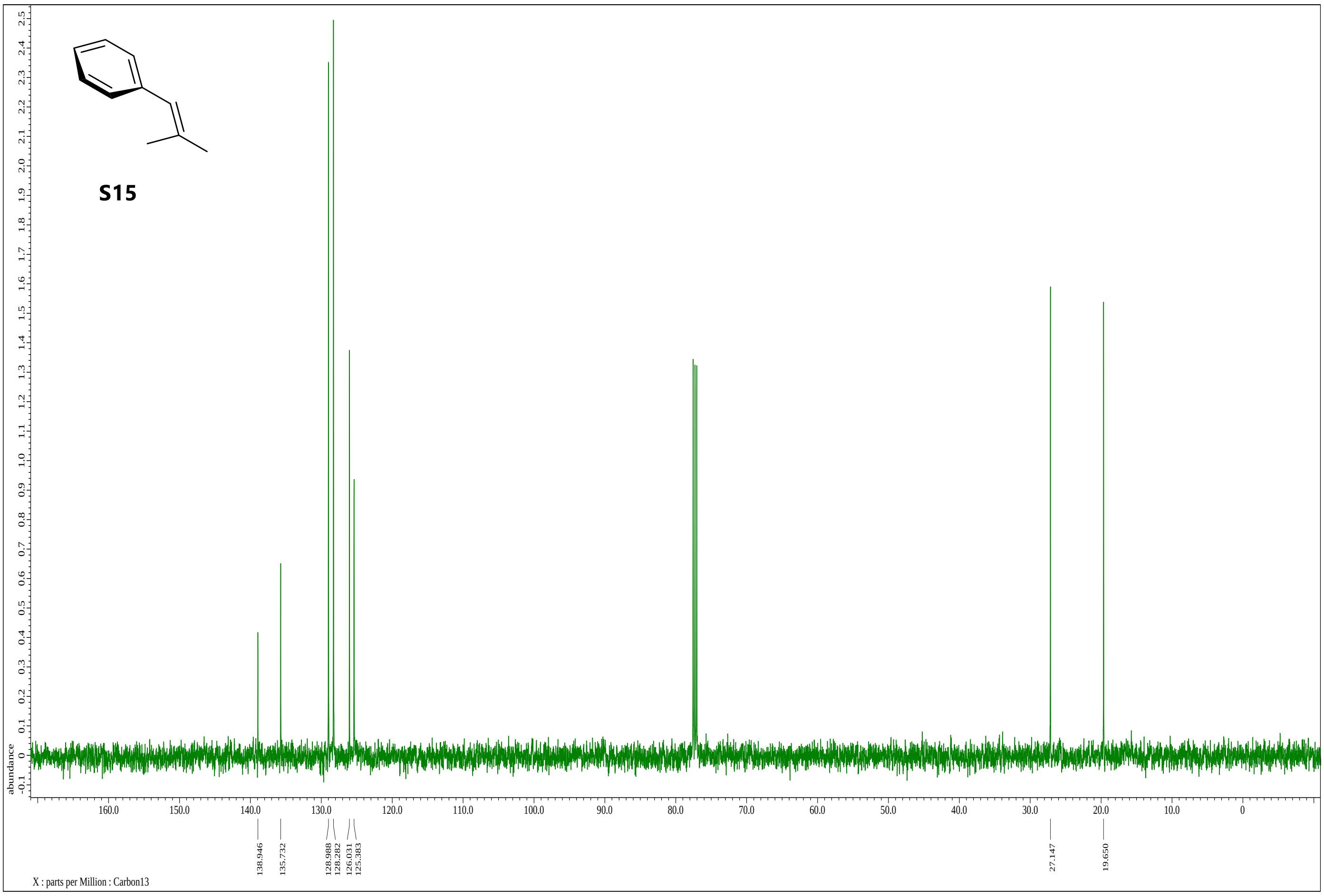

S15
abundance
X : parts per Million : Carbon13
138.946
135.732
128.988
128.282
126.031
125.383
27.147
19.650

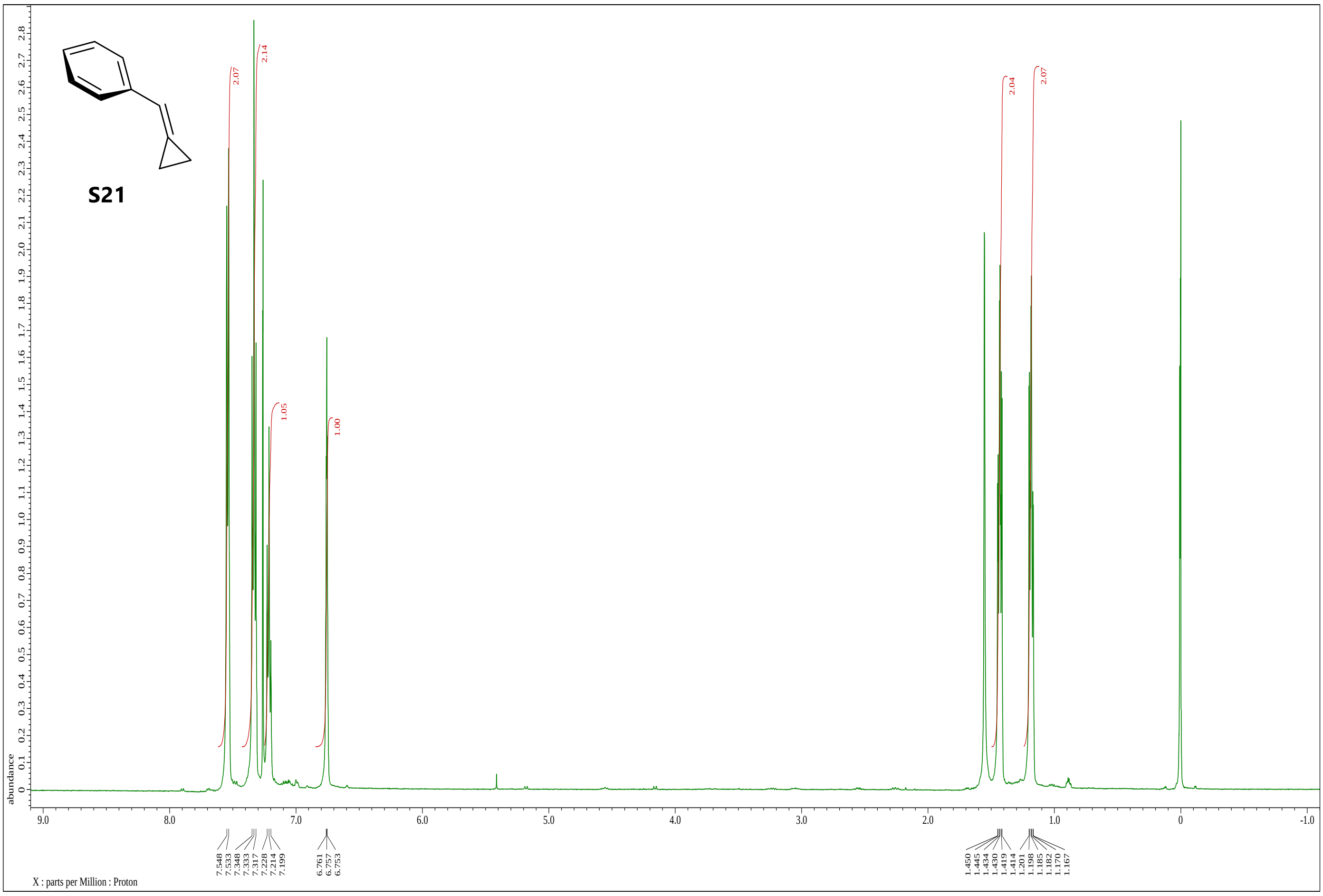
S21
abundance
X : parts per Million : Proton
2.07
2.14
1.05
1.00
2.04
2.07
7.548
7.533
7.348
7.333
7.317
7.228
7.214
7.199
6.761
6.757
6.753
1.450
1.445
1.434
1.430
1.419
1.414
1.201
1.198
1.185
1.182
1.170
1.167

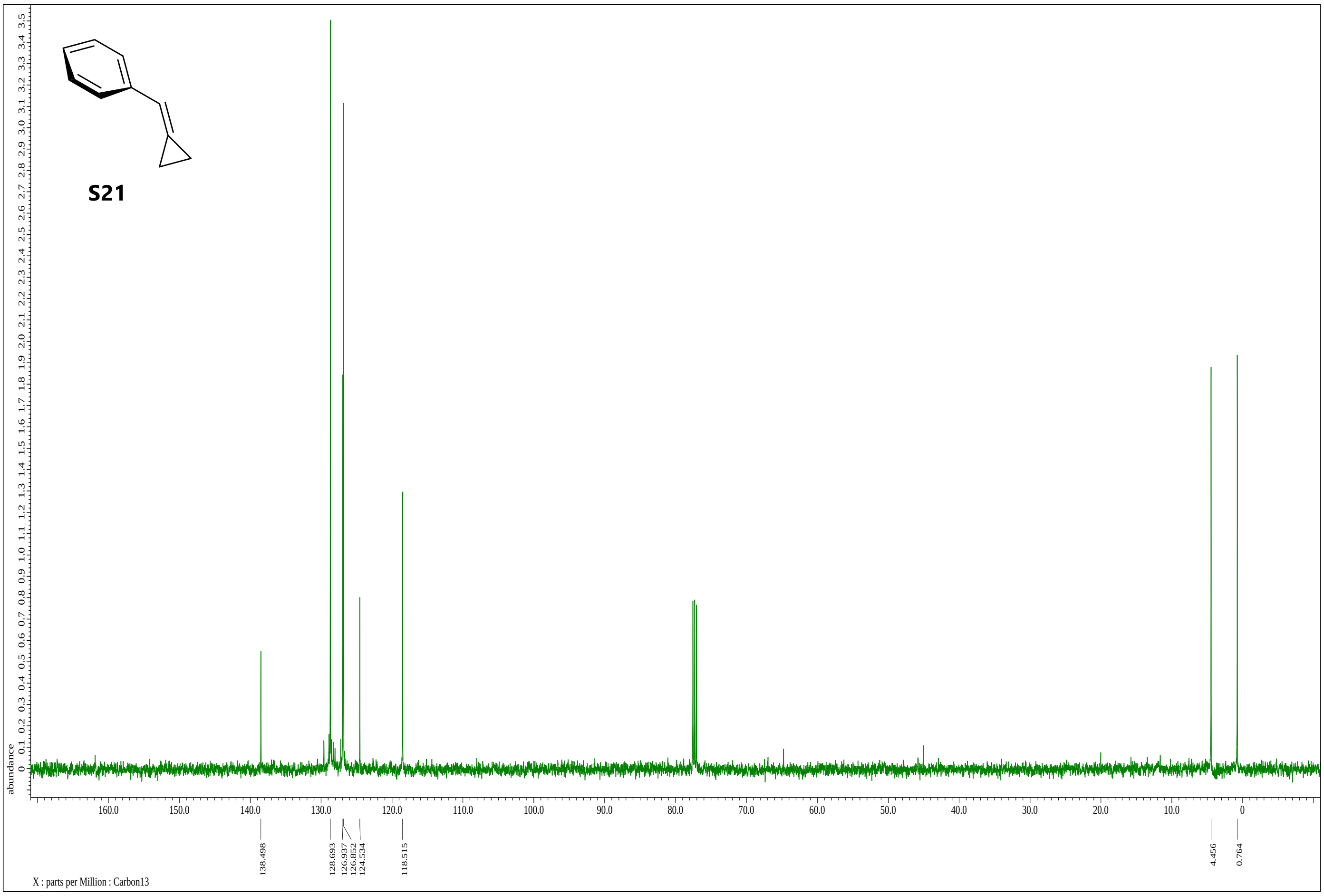
S21
abundance
X : parts per Million : Carbon13
138.498
128.693
126.937
126.852
124.534
118.515
4.456
0.764

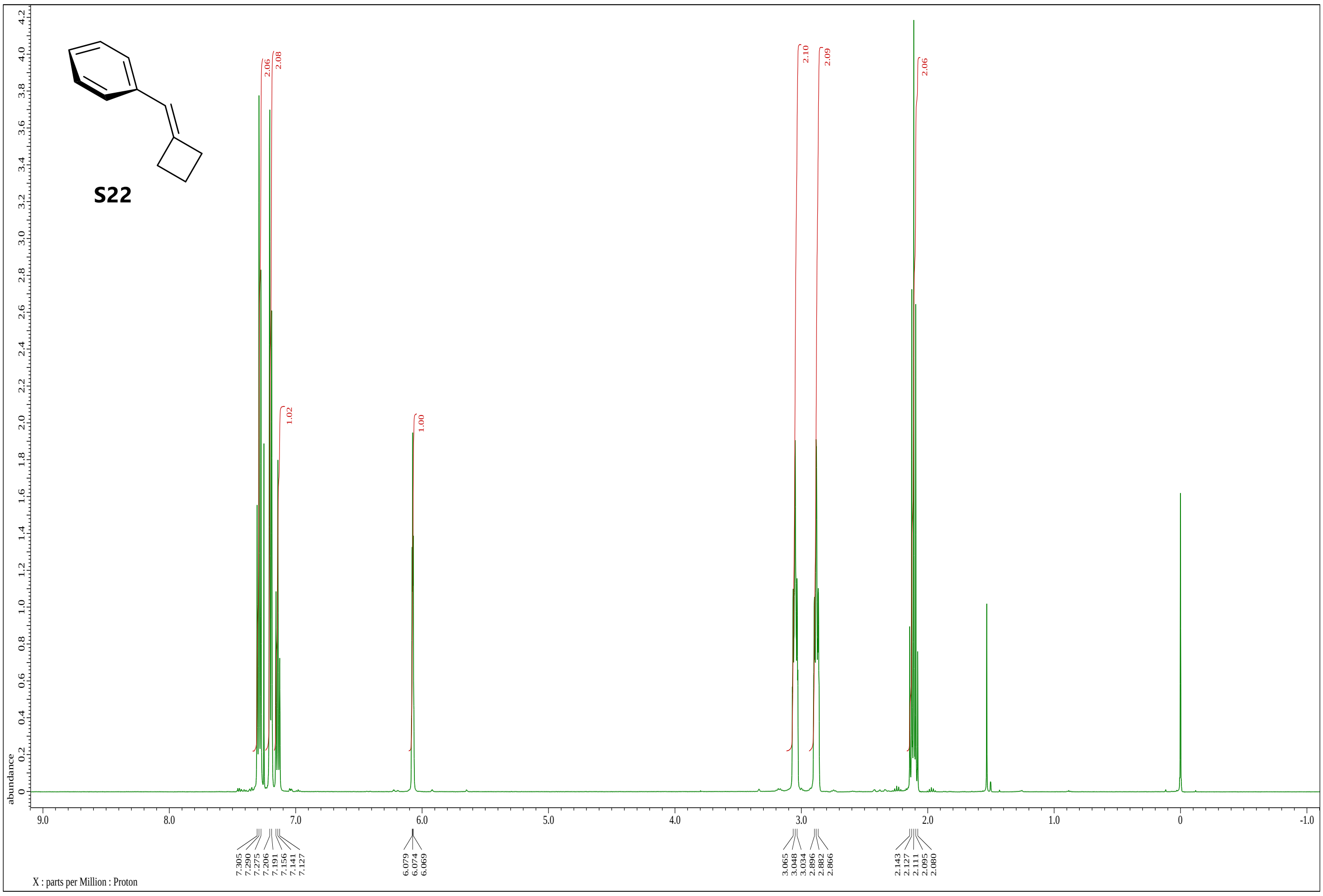
S22
2.06
2.08
1.02
1.00
2.10
2.09
2.06
abundance
7.305 7.290 7.275 7.206 7.191 7.156 7.141 7.127
6.079 6.074 6.069
3.065 3.048 3.034 2.896 2.882 2.866
2.143 2.127 2.111 2.095 2.080
X : parts per Million : Proton

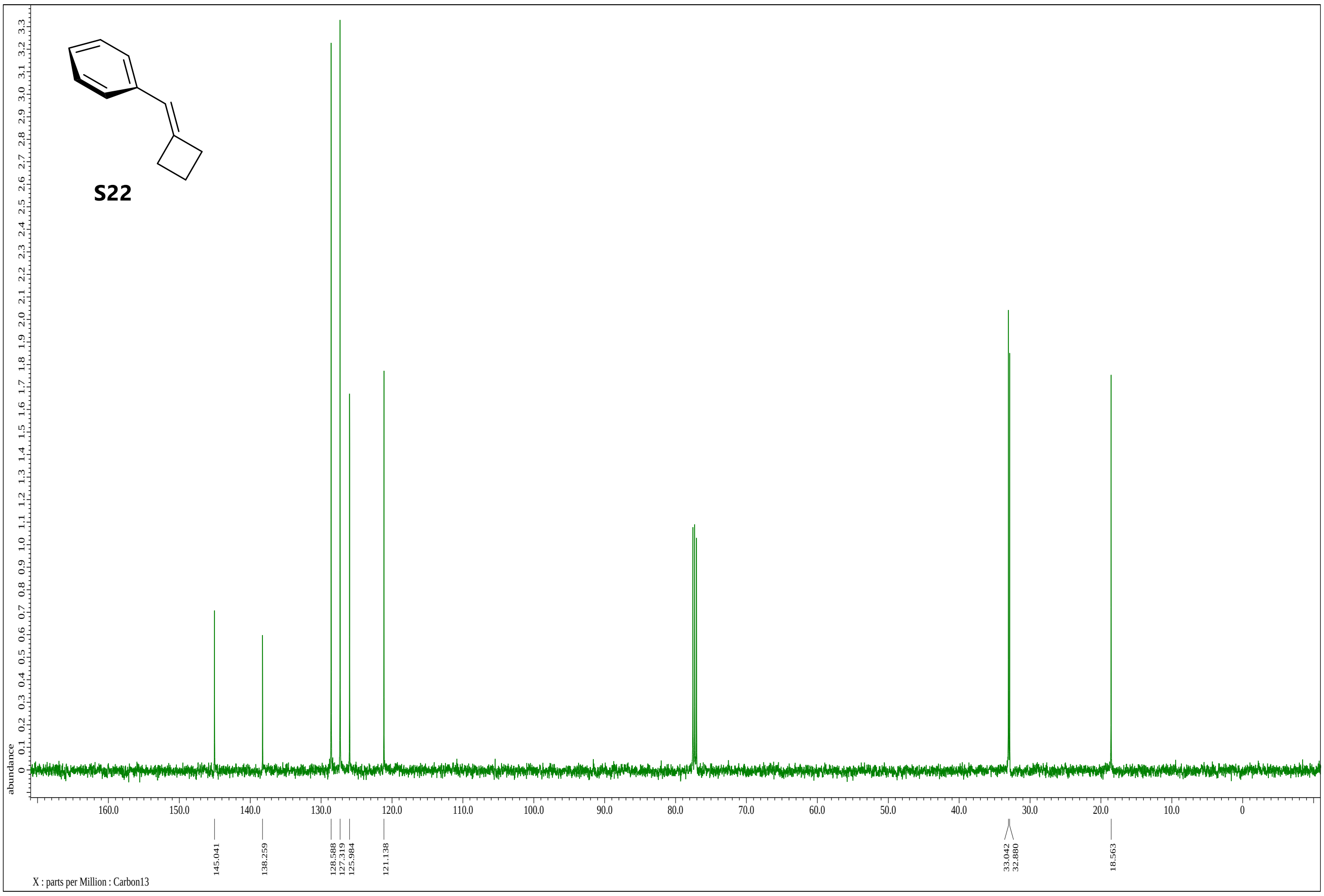
S22
abundance
145.041
138.259
128.588 127.319 125.984
121.138
33.042 32.880
18.563
X : parts per Million : Carbon13

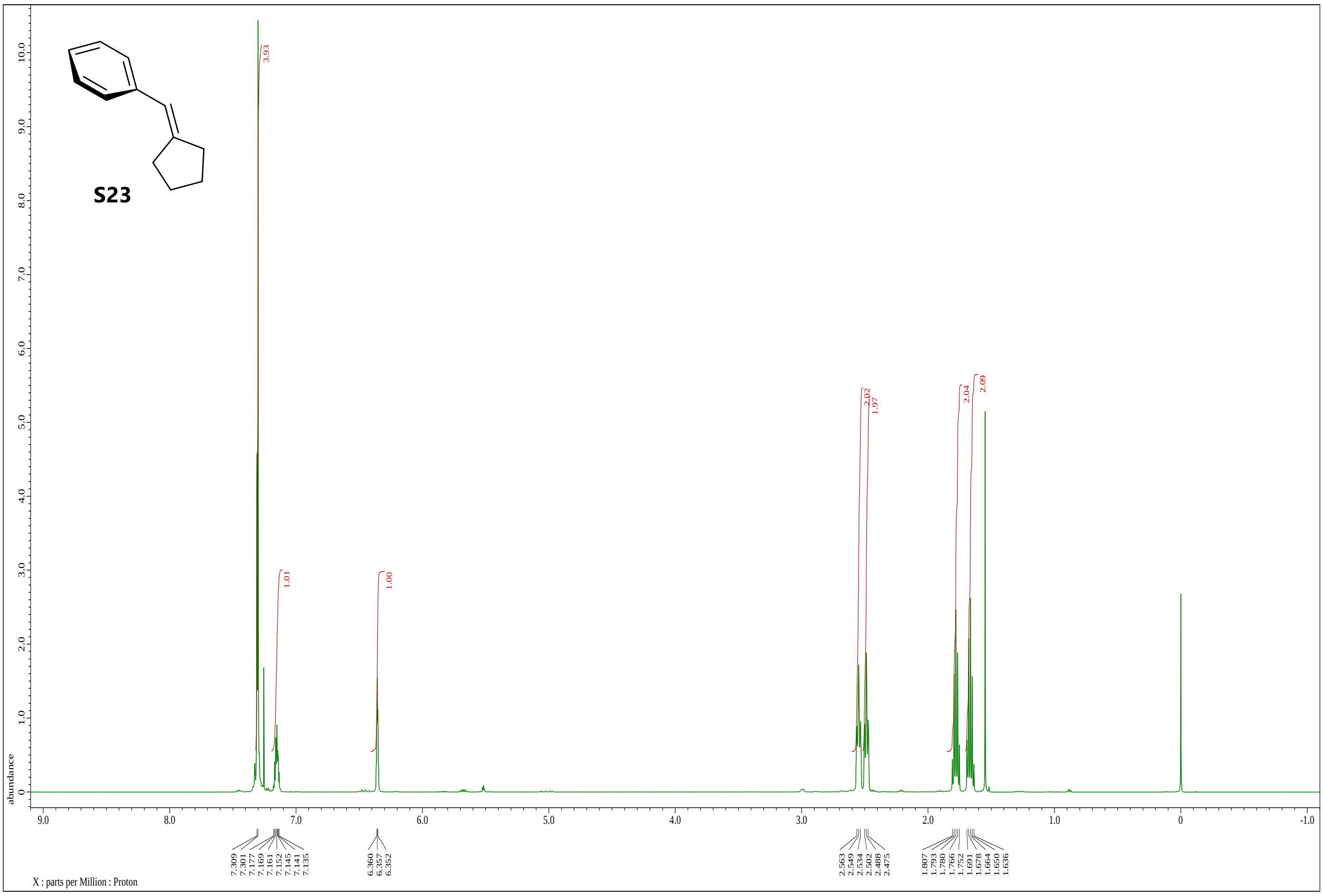
S23
3.93
1.01
1.00
2.02
1.97
2.04
2.09
abundance
7.309 7.301 7.177 7.169 7.161 7.152 7.145 7.141 7.135
6.360 6.357 6.352
2.563 2.549 2.534 2.502 2.488 2.475
1.807 1.793 1.780 1.766 1.752 1.691 1.678 1.664 1.650 1.636
X : parts per Million : Proton

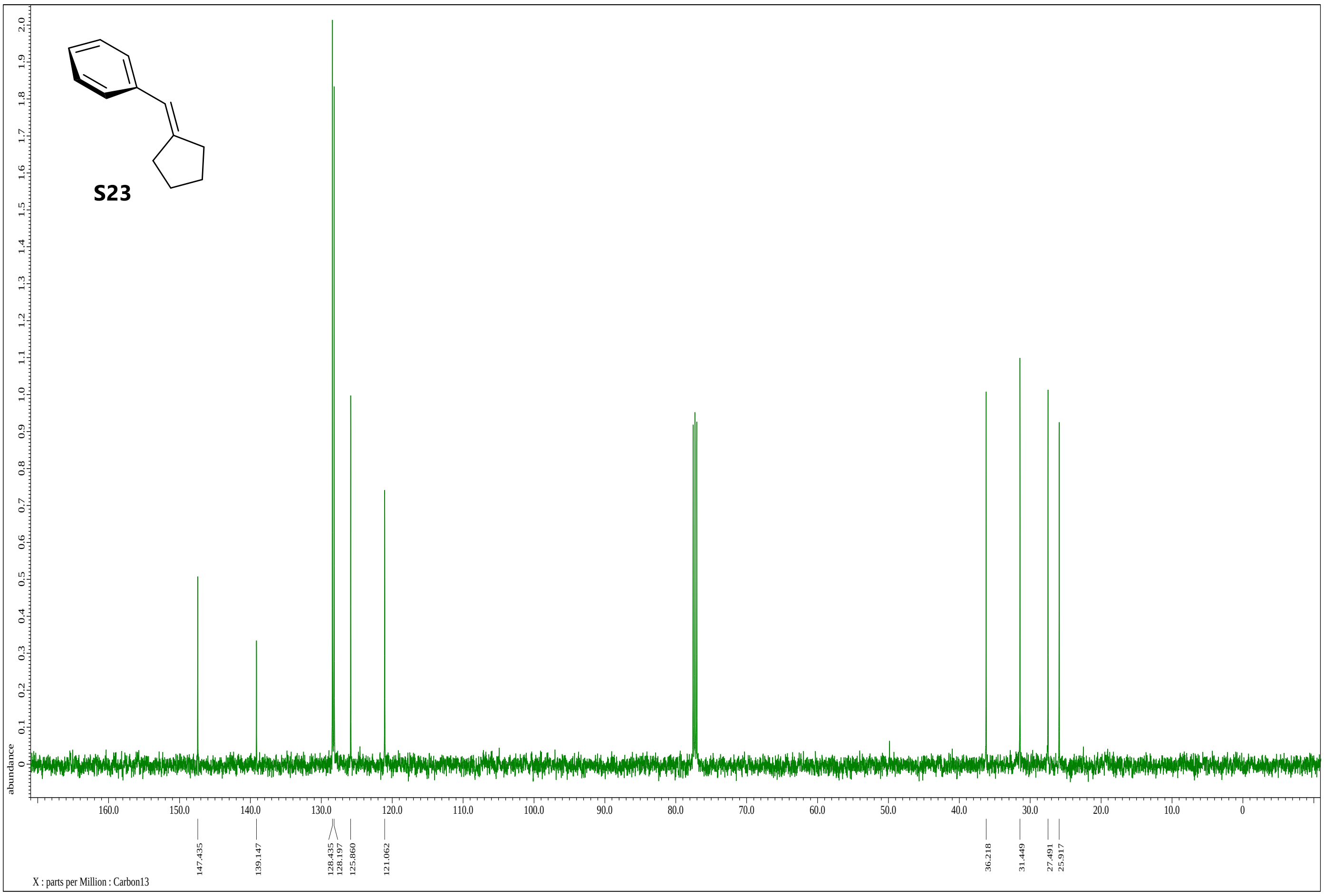
S23
abundance
147.435
139.147
128.435 128.197 125.860
121.062
36.218
31.449
27.491 25.917
X : parts per Million : Carbon13

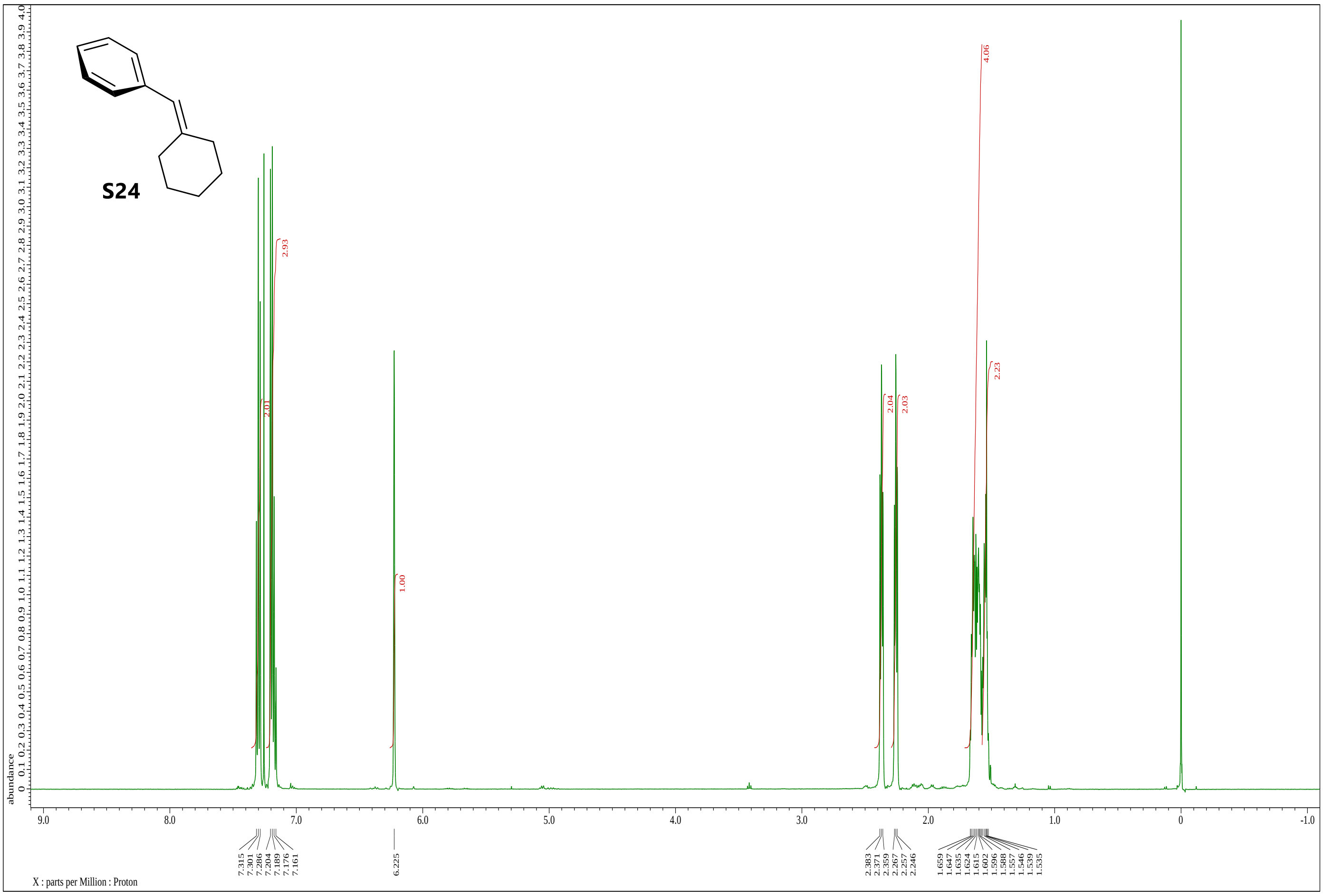
S24
2.01
2.93
1.00
2.04
2.03
4.06
2.23
7.315
7.301
7.286
7.204
7.189
7.176
7.161
6.225
2.383
2.371
2.359
2.267
2.257
2.246
1.659
1.647
1.635
1.624
1.615
1.602
1.596
1.588
1.557
1.546
1.539
1.535
abundance
X : parts per Million : Proton

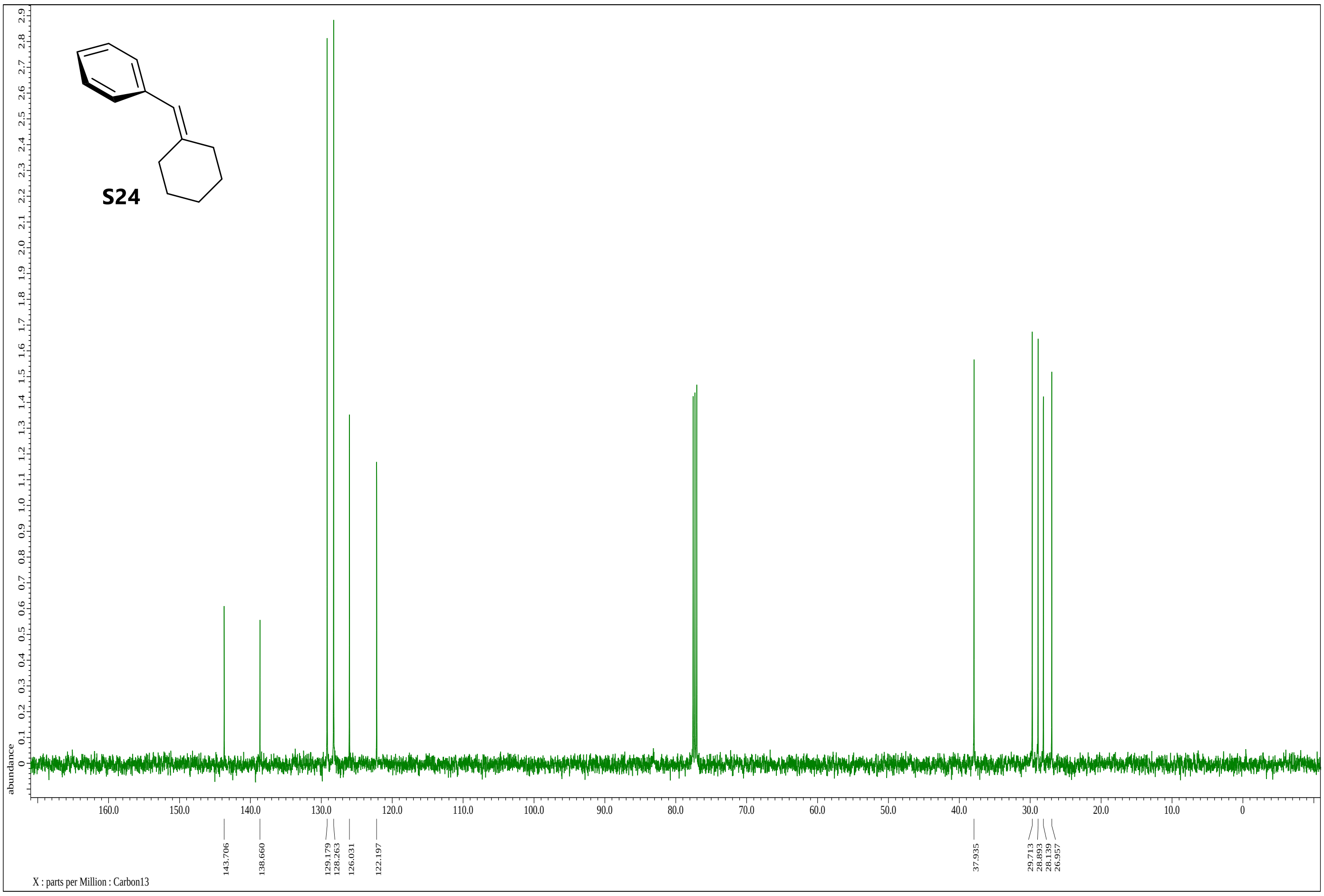
S24
143.706
138.660
129.179
128.263
126.031
122.197
37.935
29.713
28.893
28.139
26.957
abundance
X : parts per Million : Carbon13

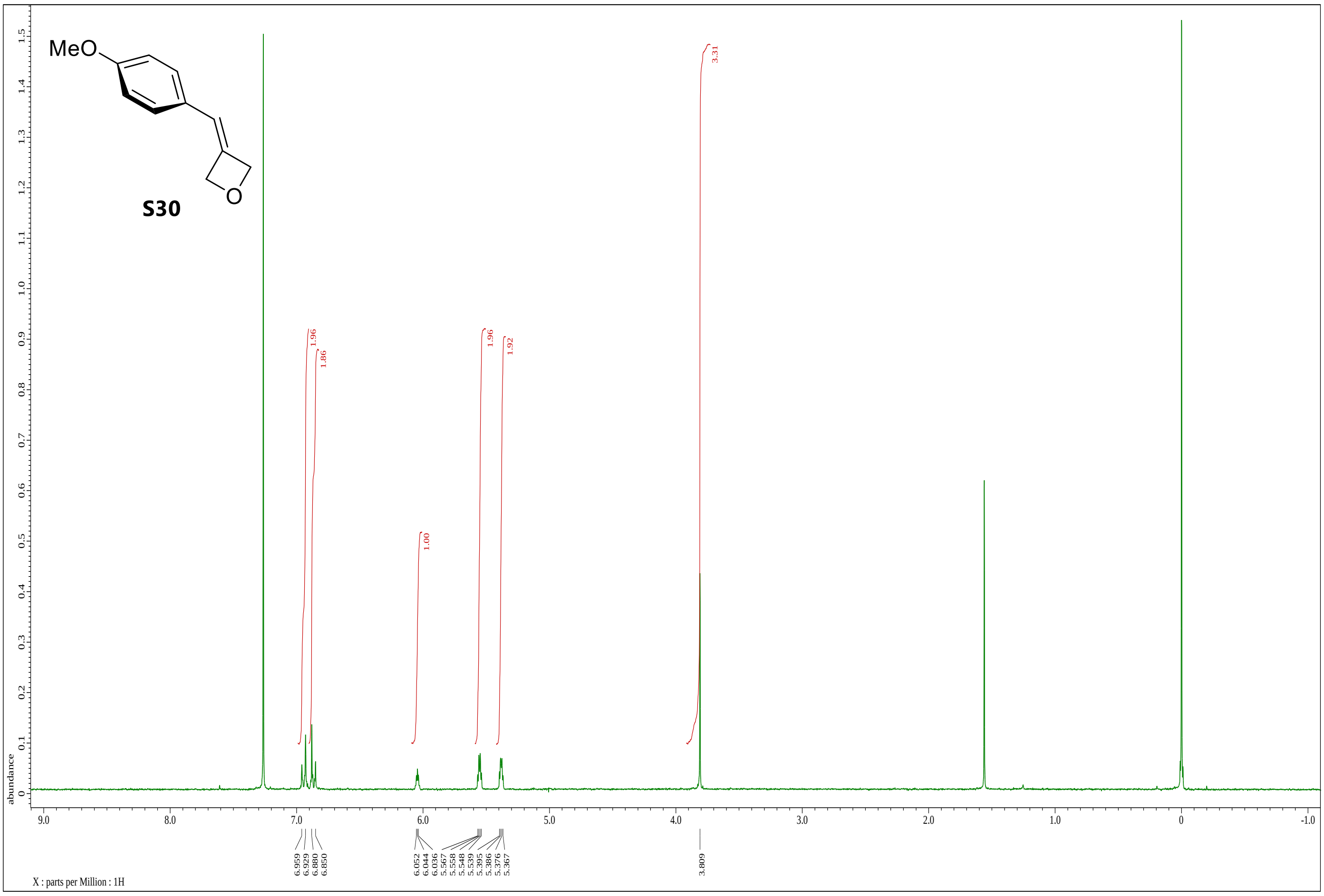
MeO
S30
X : parts per Million : 1H
abundance
6.959
6.929
6.880
6.850
6.052
6.044
6.036
5.567
5.558
5.548
5.539
5.395
5.386
5.376
5.367
3.809
1.96
1.86
1.00
1.96
1.92
3.31

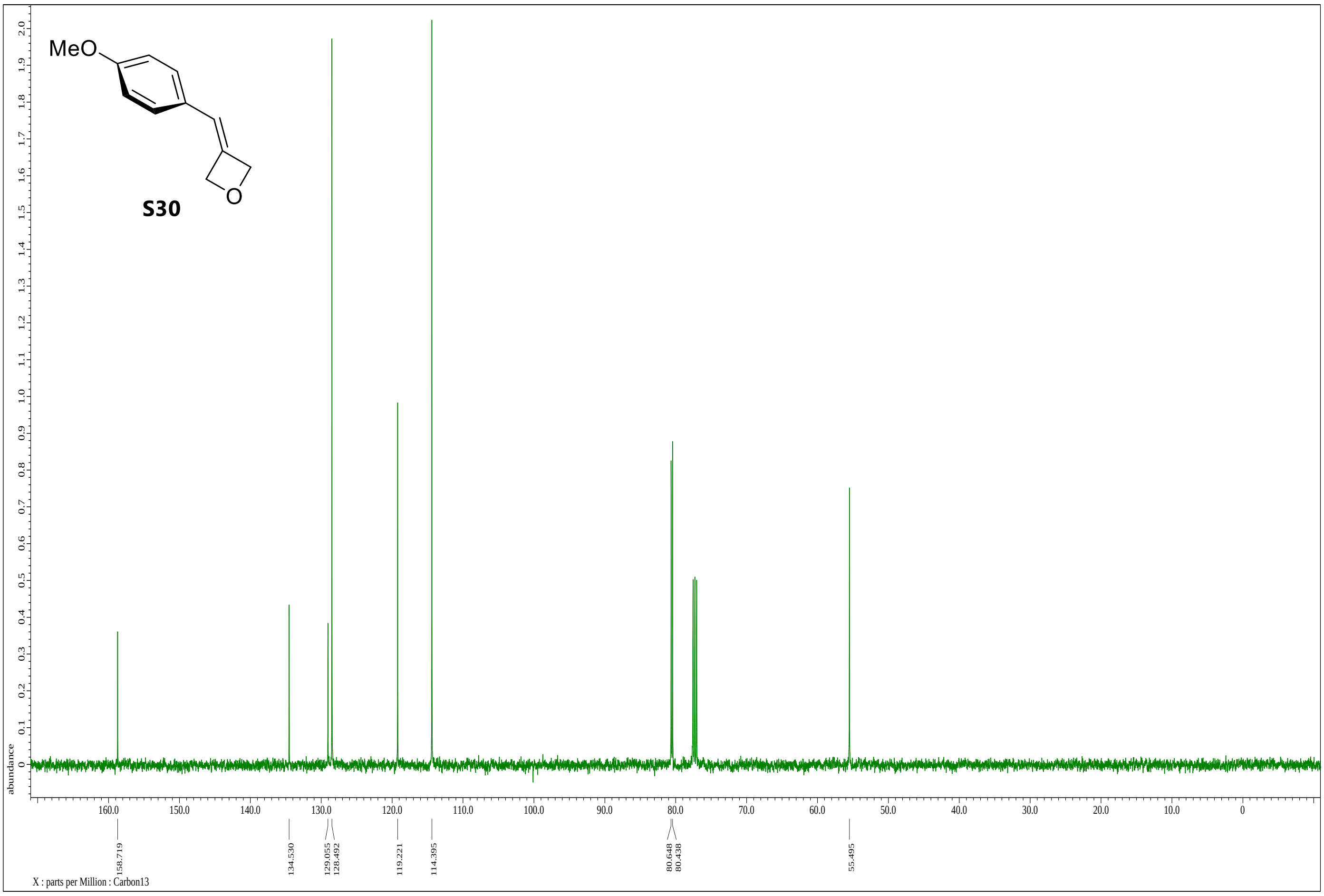
MeO
S30
X : parts per Million : Carbon13
abundance
158.719
134.530
129.055
128.492
119.221
114.395
80.648
80.438
55.495

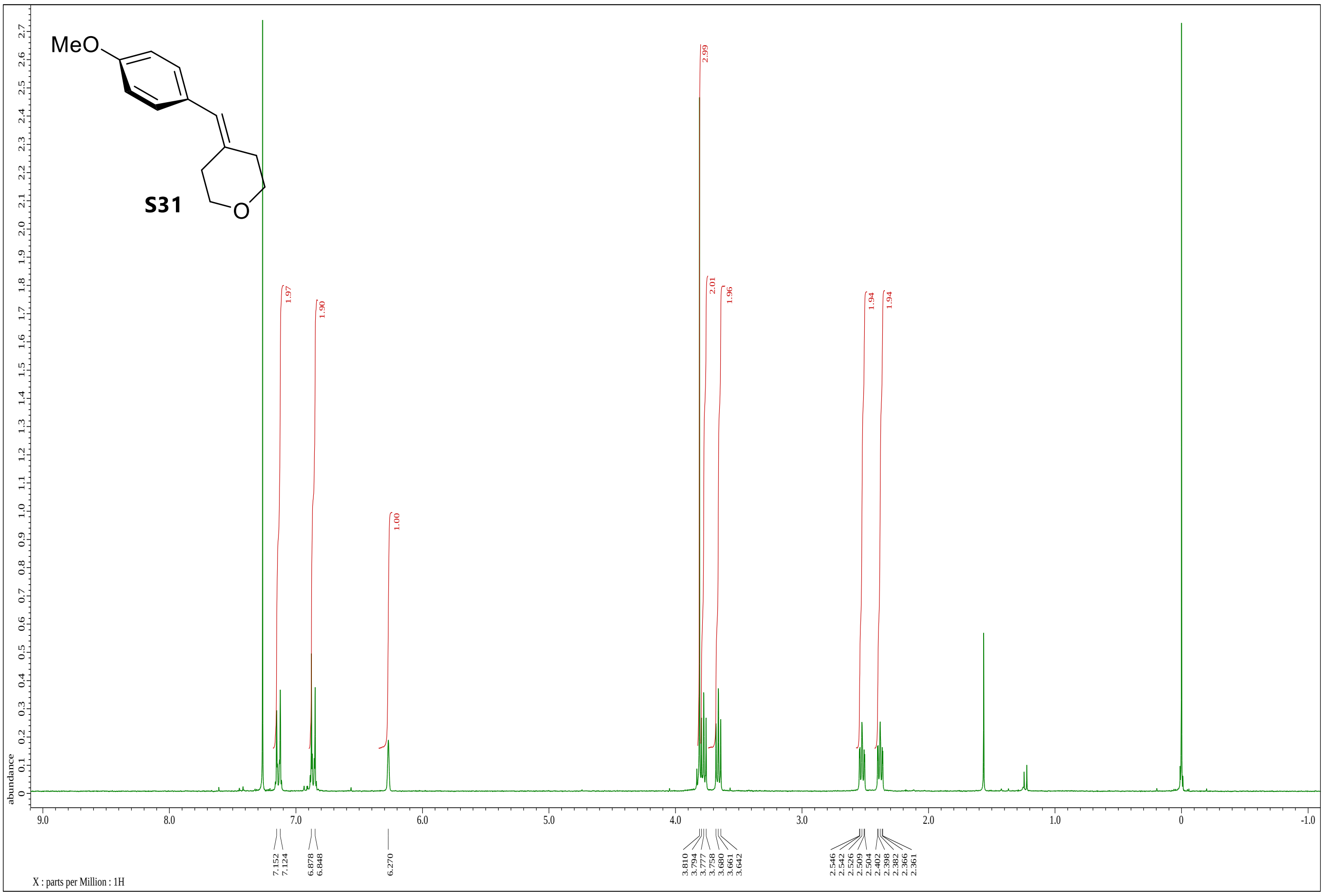
MeO
S31
O
abundance
X : parts per Million : 1H
2.99
2.01
1.96
1.97
1.90
1.00
1.94
1.94
7.152
7.124
6.878
6.848
6.270
3.810
3.794
3.777
3.758
3.680
3.661
3.642
2.546
2.542
2.526
2.509
2.504
2.402
2.398
2.382
2.366
2.361

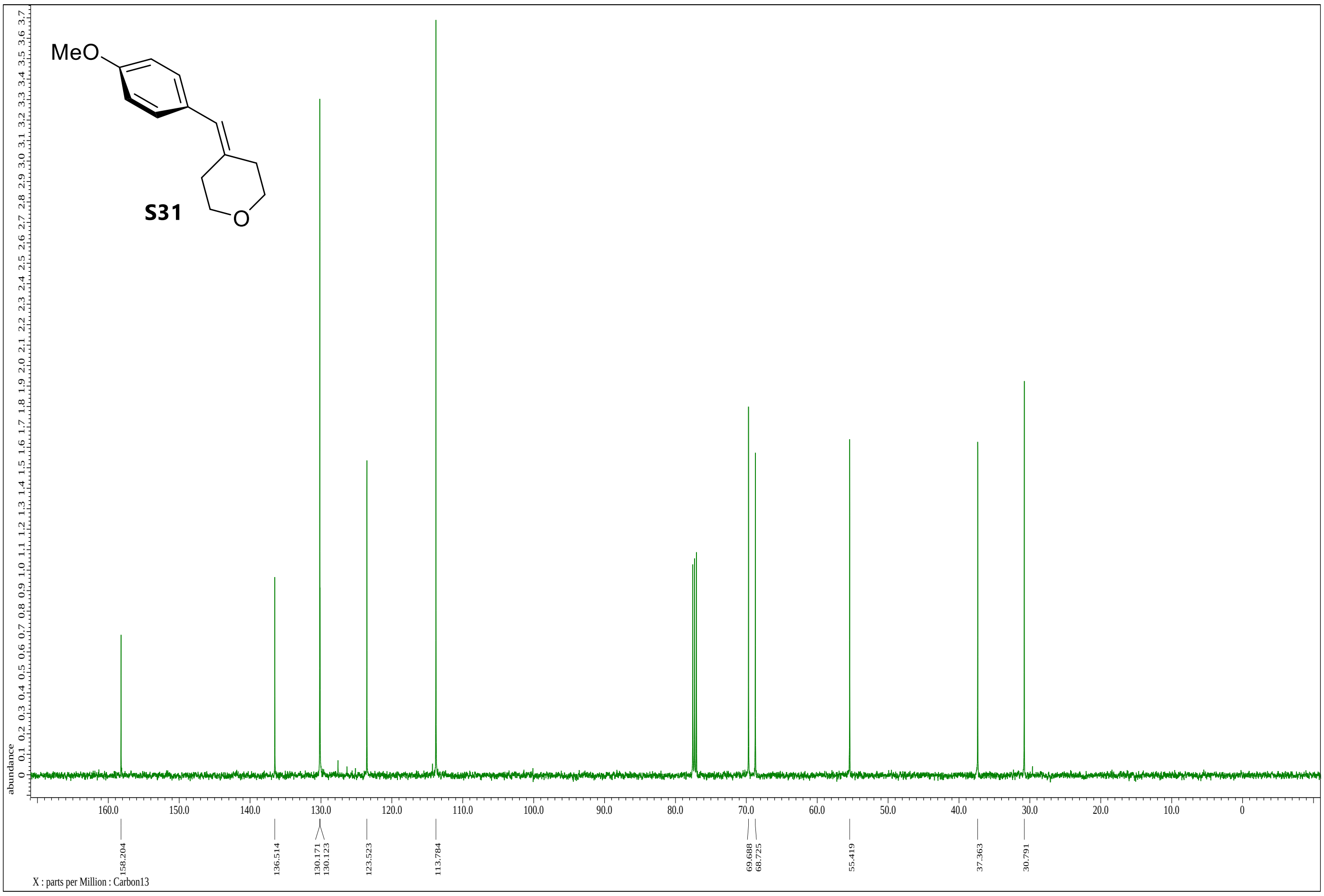
MeO
S31
O
abundance
X : parts per Million : Carbon13
158.204
136.514
130.171
130.123
123.523
113.784
69.688
68.725
55.419
37.363
30.791

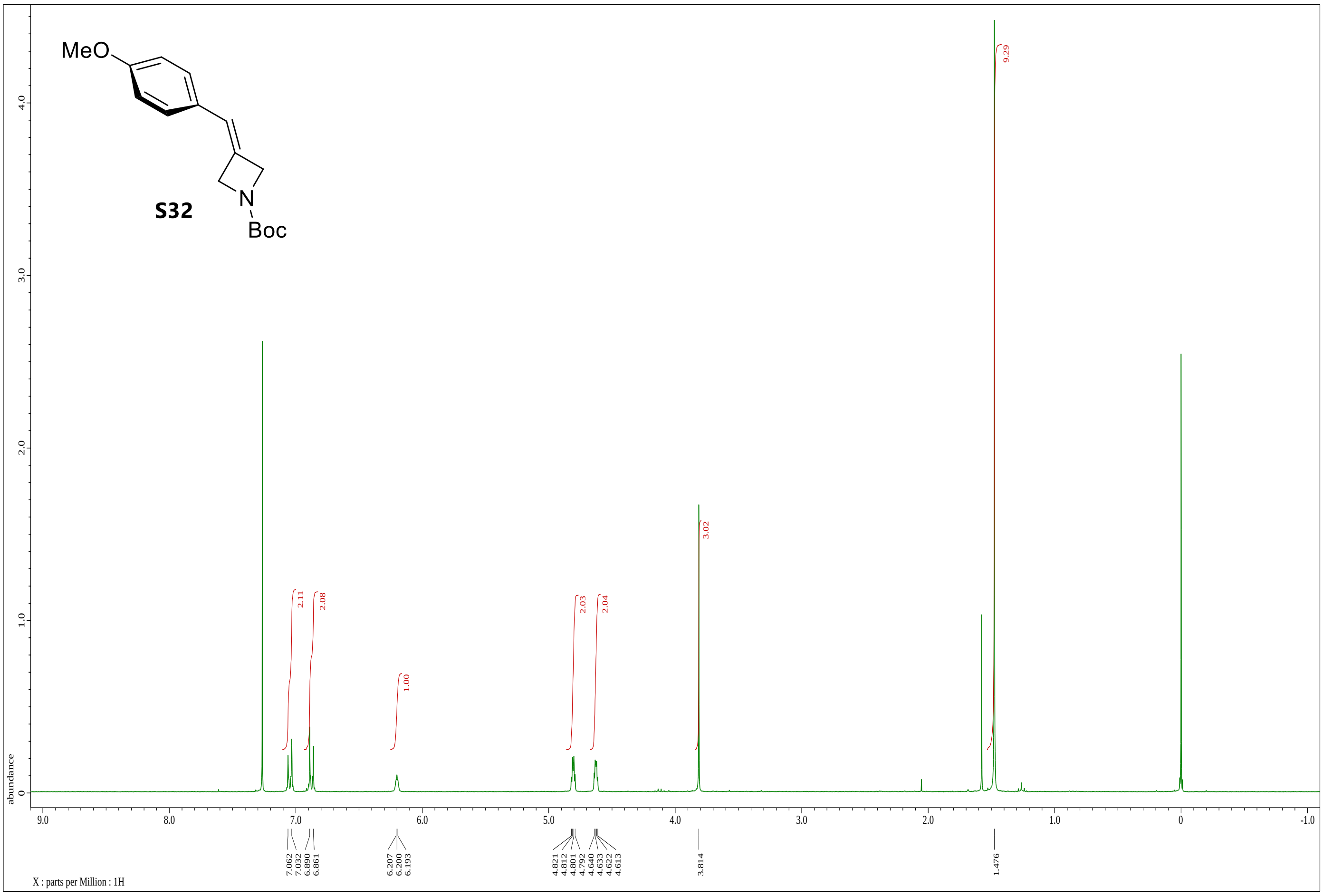
MeO
S32
N
Boc
abundance
9.29
3.02
2.11
2.08
1.00
2.03
2.04
9.0 8.0 7.0 6.0 5.0 4.0 3.0 2.0 1.0 0 -1.0
7.062 7.032 6.890 6.861
6.207 6.200 6.193
4.821 4.812 4.801 4.792 4.640 4.633 4.622 4.613
3.814
1.476
X : parts per Million : 1H

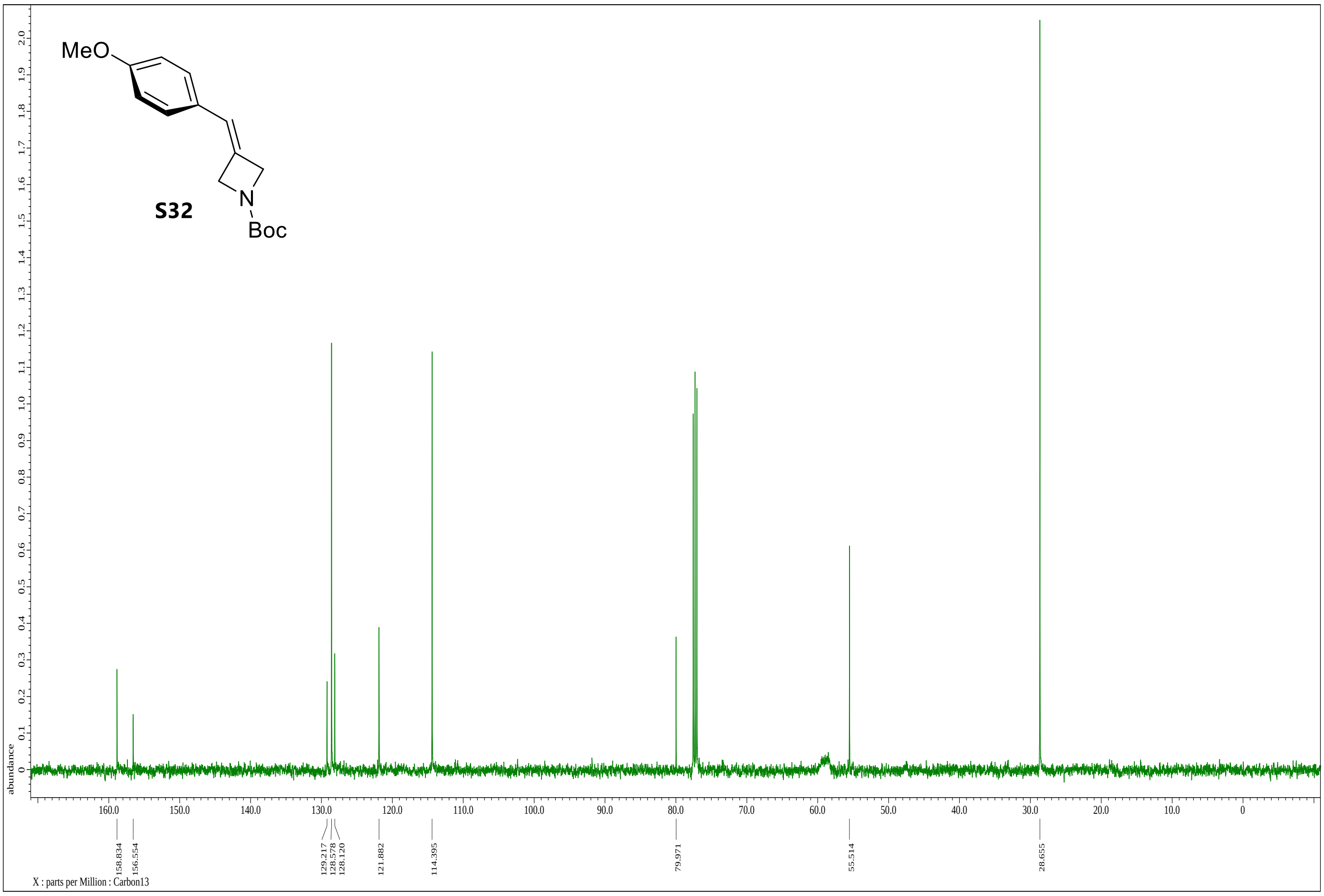
MeO
S32
N
Boc
abundance
160.0 150.0 140.0 130.0 120.0 110.0 100.0 90.0 80.0 70.0 60.0 50.0 40.0 30.0 20.0 10.0 0
158.834 156.554
129.217 128.578 128.120
121.882
114.395
79.971
55.514
28.655
X : parts per Million : Carbon13

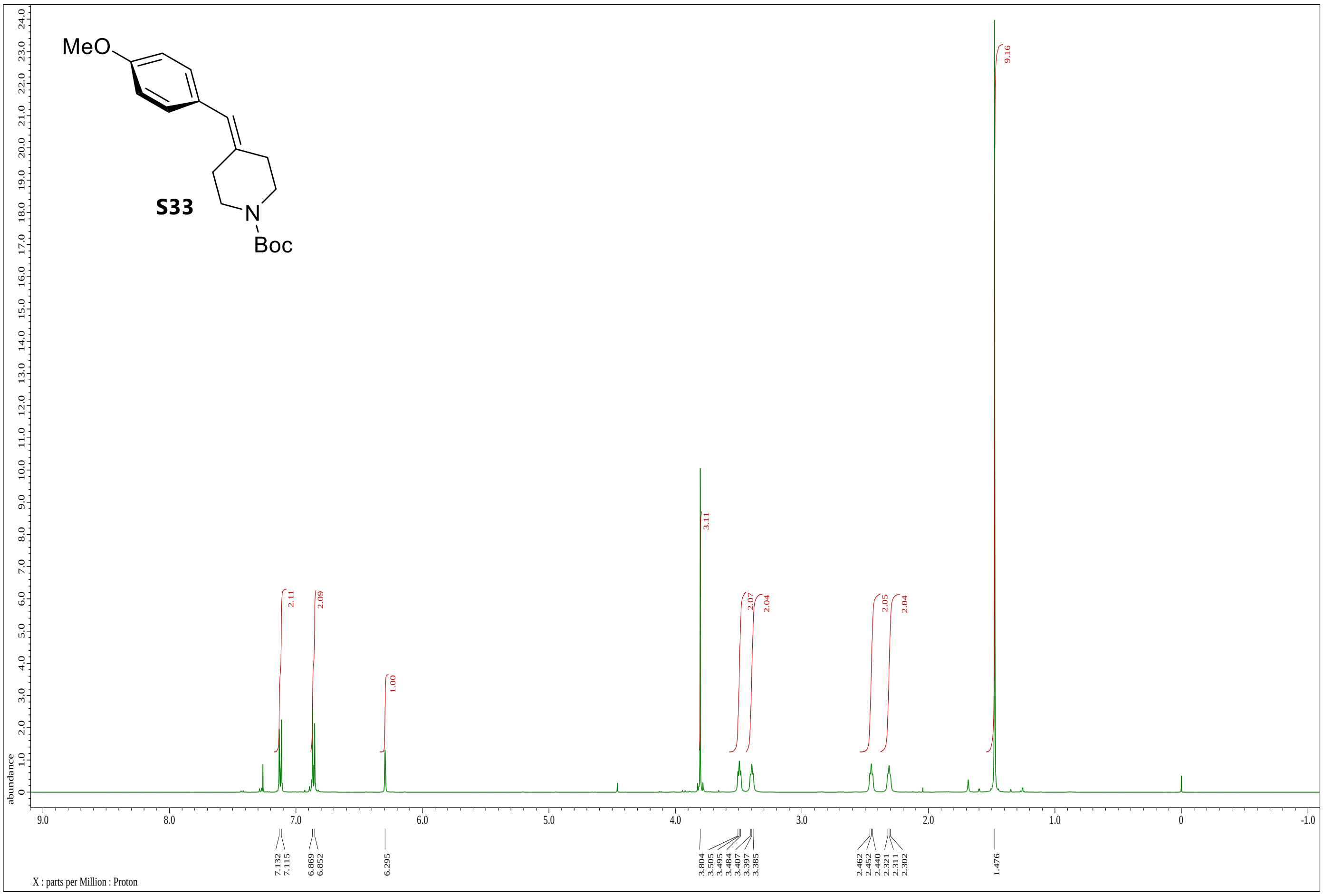
MeO
S33
N
Boc
abundance
2.11
2.09
1.00
3.11
2.07
2.04
2.05
2.04
9.16
7.132
7.115
6.869
6.852
6.295
3.804
3.505
3.495
3.484
3.407
3.397
3.385
2.462
2.452
2.440
2.321
2.311
2.302
1.476
X : parts per Million : Proton

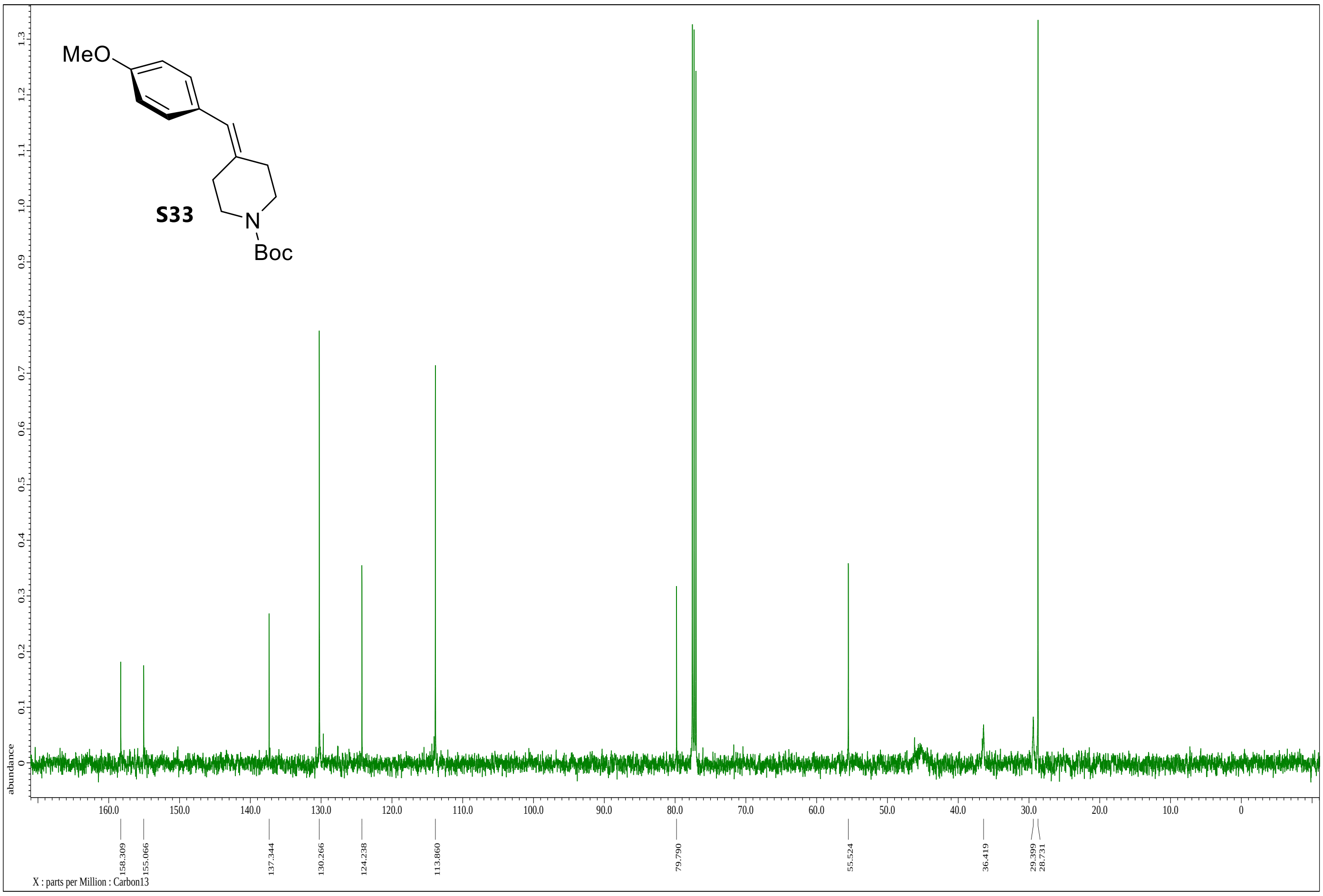
MeO
S33
N
Boc
abundance
158.309
155.066
137.344
130.266
124.238
113.860
79.790
55.524
36.419
29.399
28.731
X : parts per Million : Carbon13

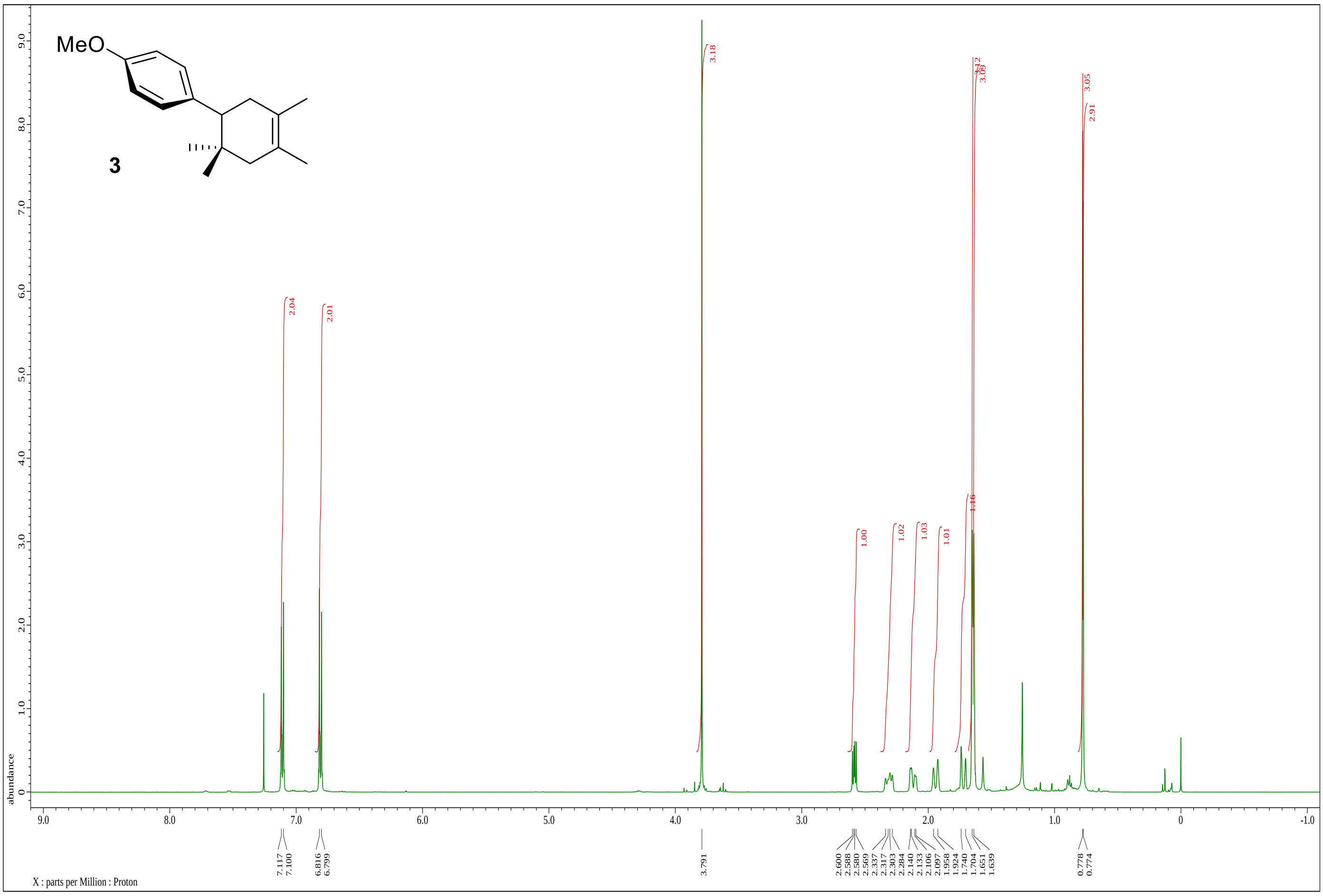
MeO
3
X : parts per Million : Proton
abundance

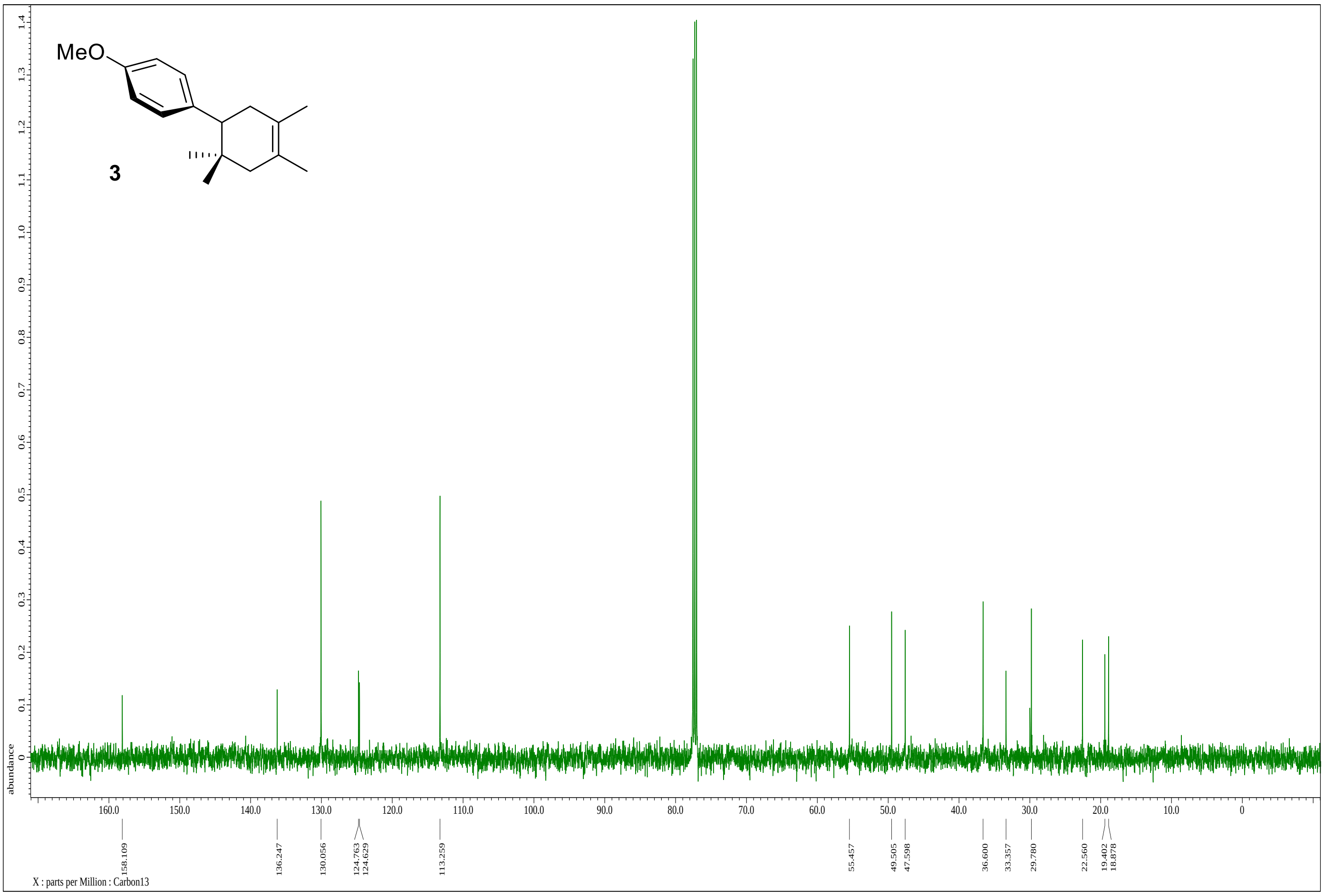
MeO
3
X : parts per Million : Carbon13
abundance

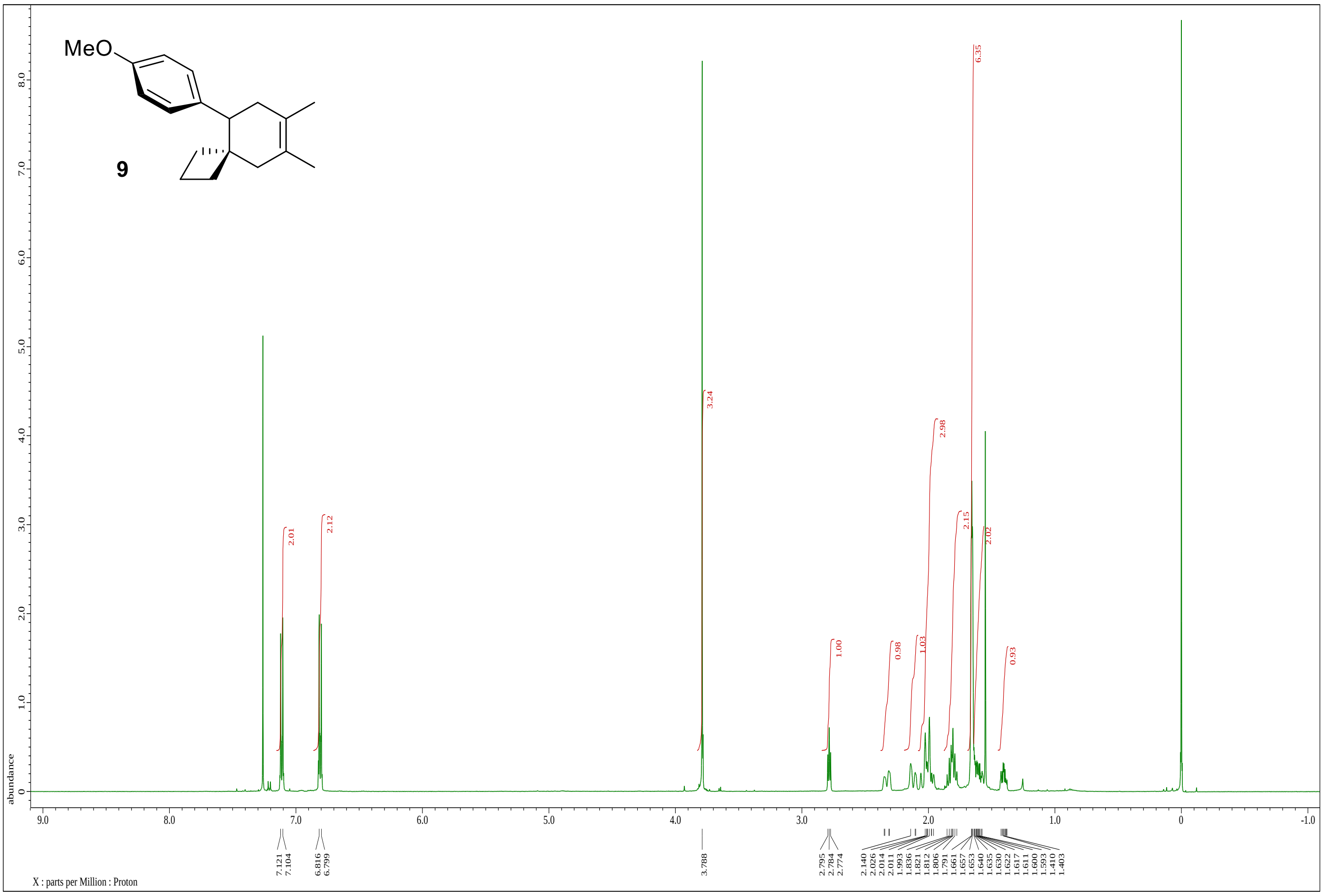
MeO
9
abundance
X : parts per Million : Proton
7.121
7.104
6.816
6.799
3.788
2.795
2.784
2.774
2.140
2.026
2.014
2.011
1.993
1.836
1.821
1.812
1.806
1.791
1.661
1.657
1.653
1.640
1.635
1.630
1.622
1.617
1.611
1.600
1.593
1.410
1.403
2.01
2.12
3.24
1.00
0.98
1.03
2.98
2.15
6.35
2.02
0.93

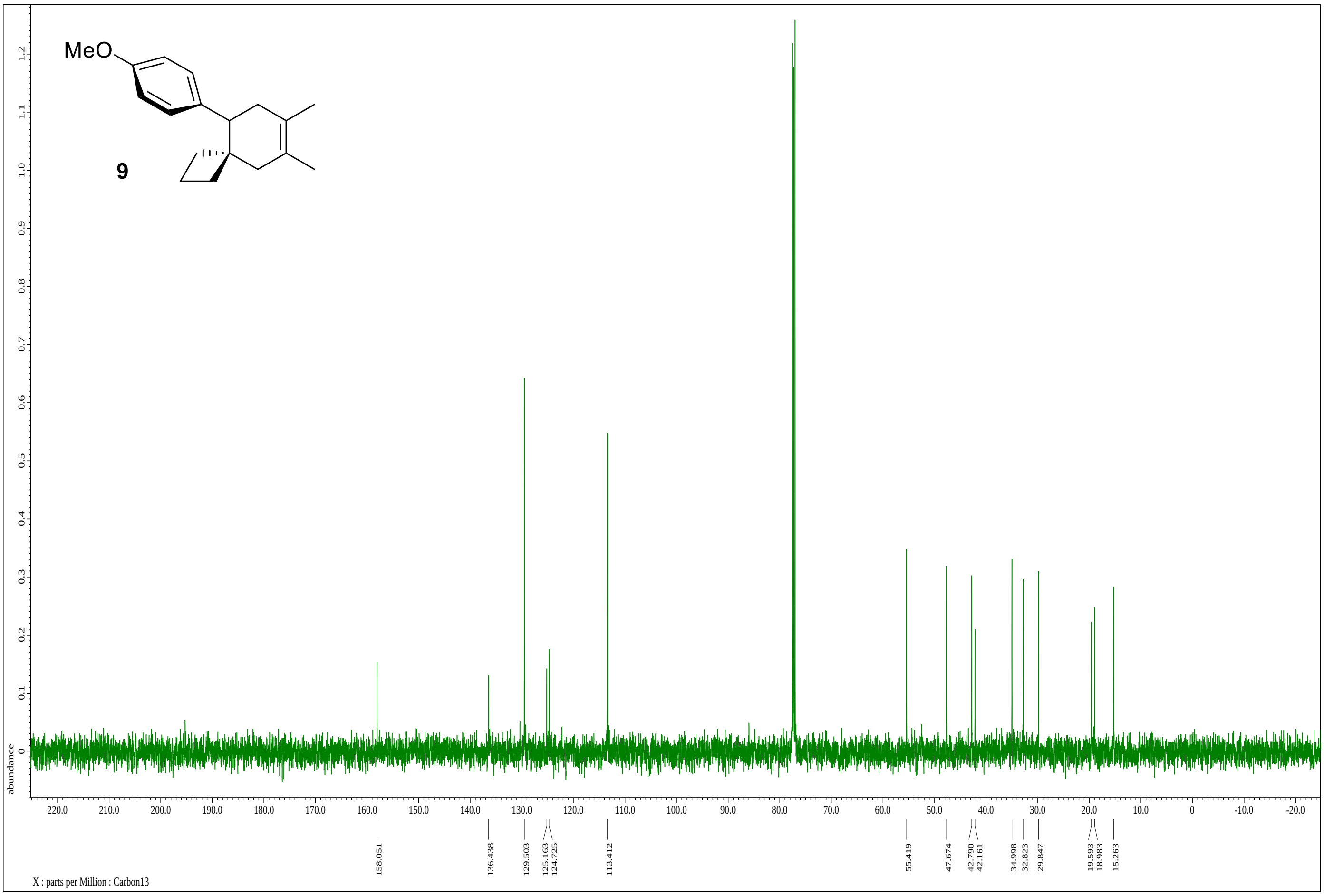
MeO
9
abundance
X : parts per Million : Carbon13
158.051
136.438
129.503
125.163
124.725
113.412
55.419
47.674
42.790
42.161
34.998
32.823
29.847
19.593
18.983
15.263

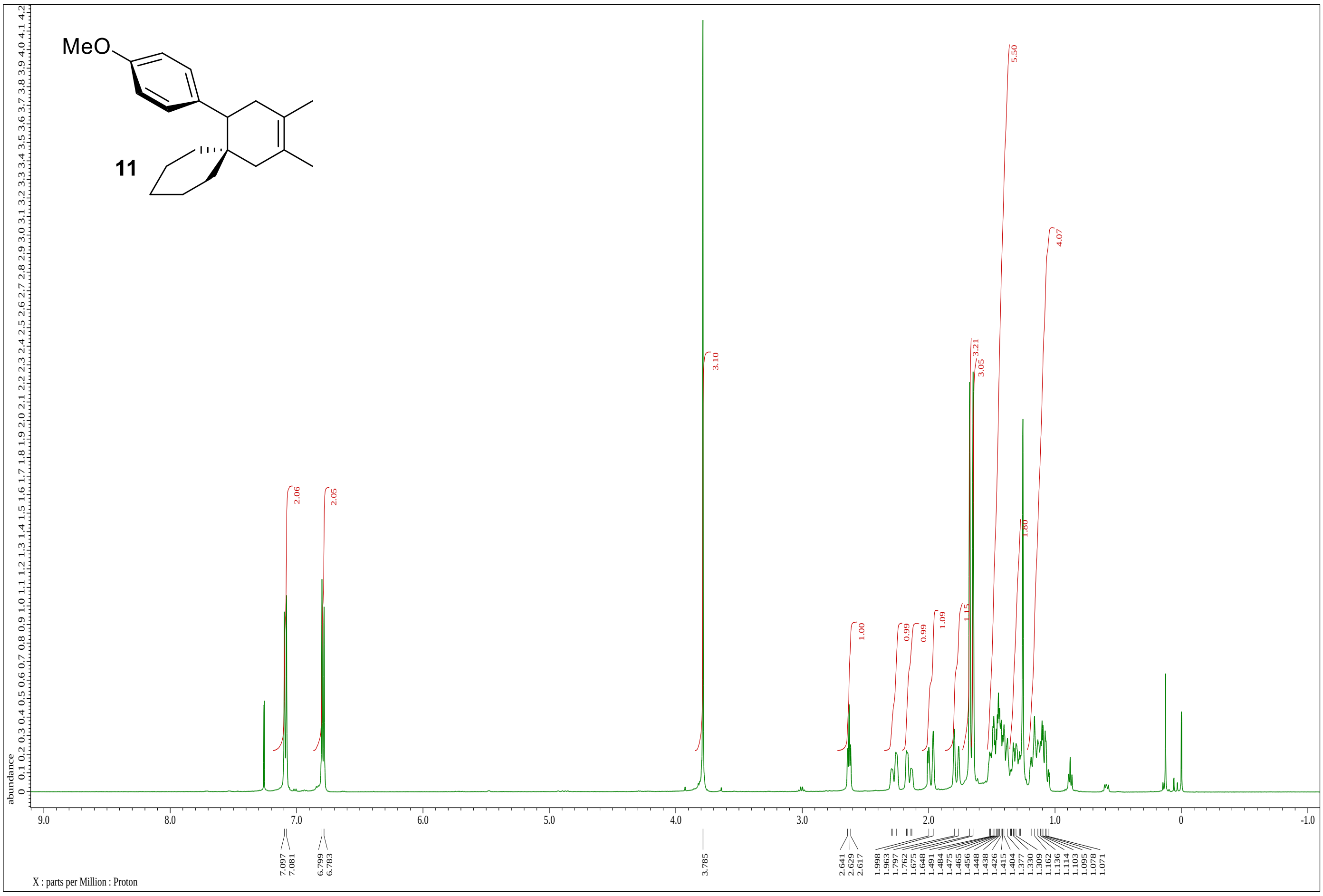
MeO
11
abundance
2.06
2.05
3.10
1.00
0.99
0.99
1.09
1.15
3.21
3.05
5.50
1.00
4.07
7.097
7.081
6.799
6.783
3.785
2.641
2.629
2.617
1.998
1.963
1.797
1.762
1.675
1.648
1.491
1.484
1.475
1.465
1.456
1.448
1.438
1.426
1.415
1.404
1.377
1.330
1.309
1.162
1.136
1.114
1.103
1.095
1.078
1.071
X : parts per Million : Proton

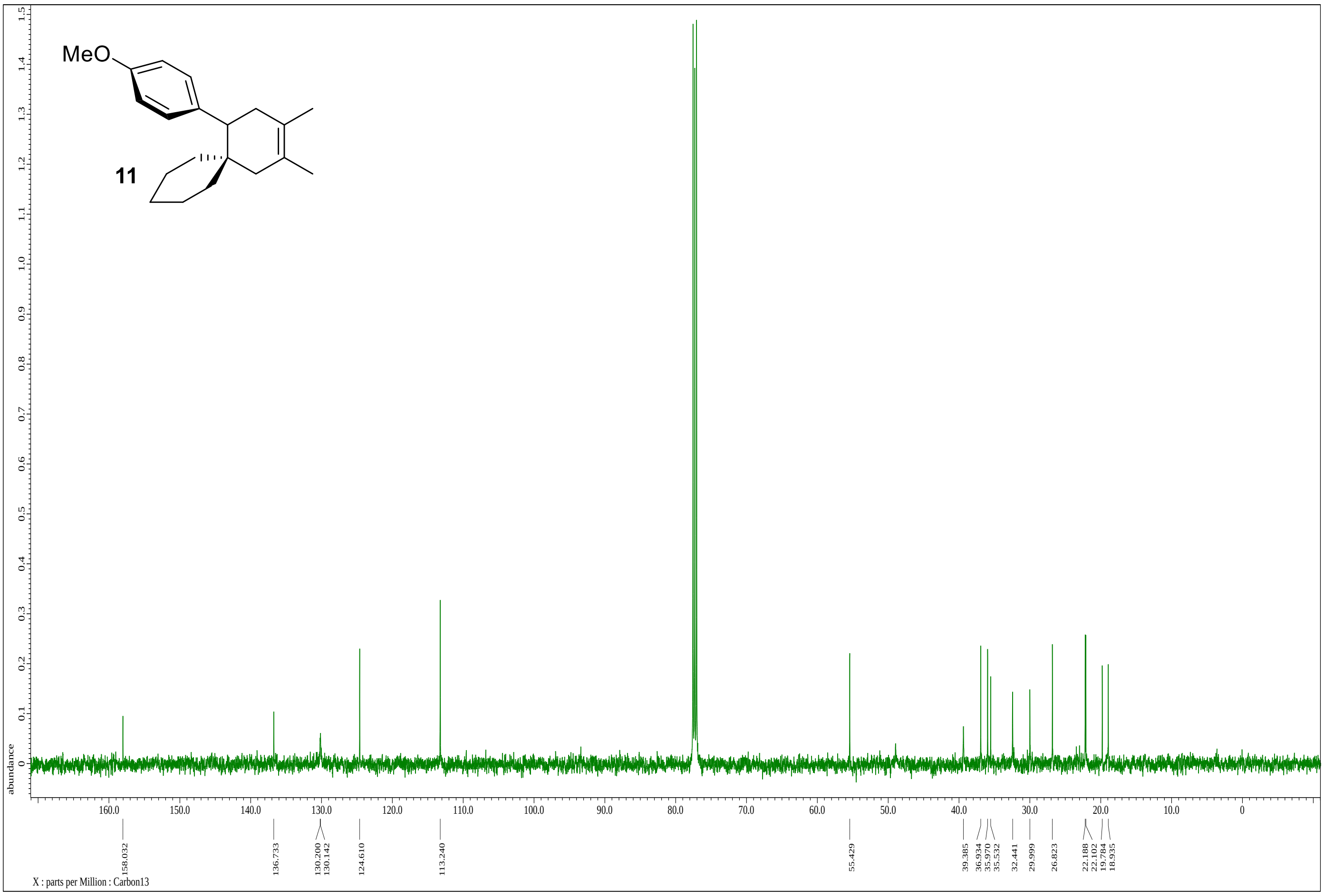
MeO
11
abundance
158.032
136.733
130.200
130.142
124.610
113.240
55.429
39.385
36.934
35.970
35.532
32.441
29.999
26.823
22.188
22.102
19.784
18.935
X : parts per Million : Carbon13

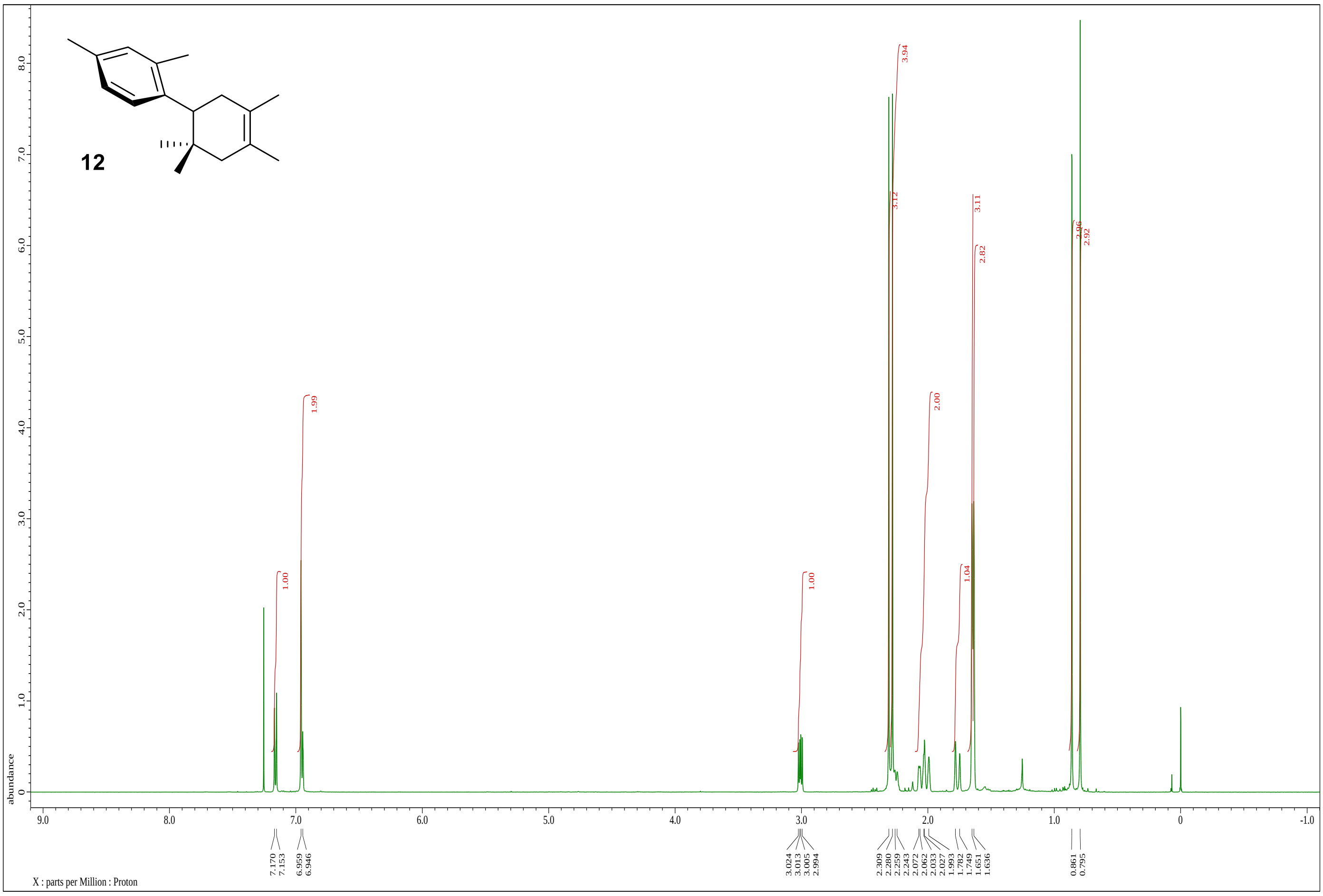
12
abundance
X : parts per Million : Proton
7.170
7.153
6.959
6.946
3.024
3.013
3.005
2.994
2.309
2.280
2.259
2.243
2.072
2.062
2.033
2.027
1.993
1.782
1.749
1.651
1.636
0.861
0.795
1.00
1.99
1.00
3.94
3.12
2.00
1.04
3.11
2.82
2.96
2.92

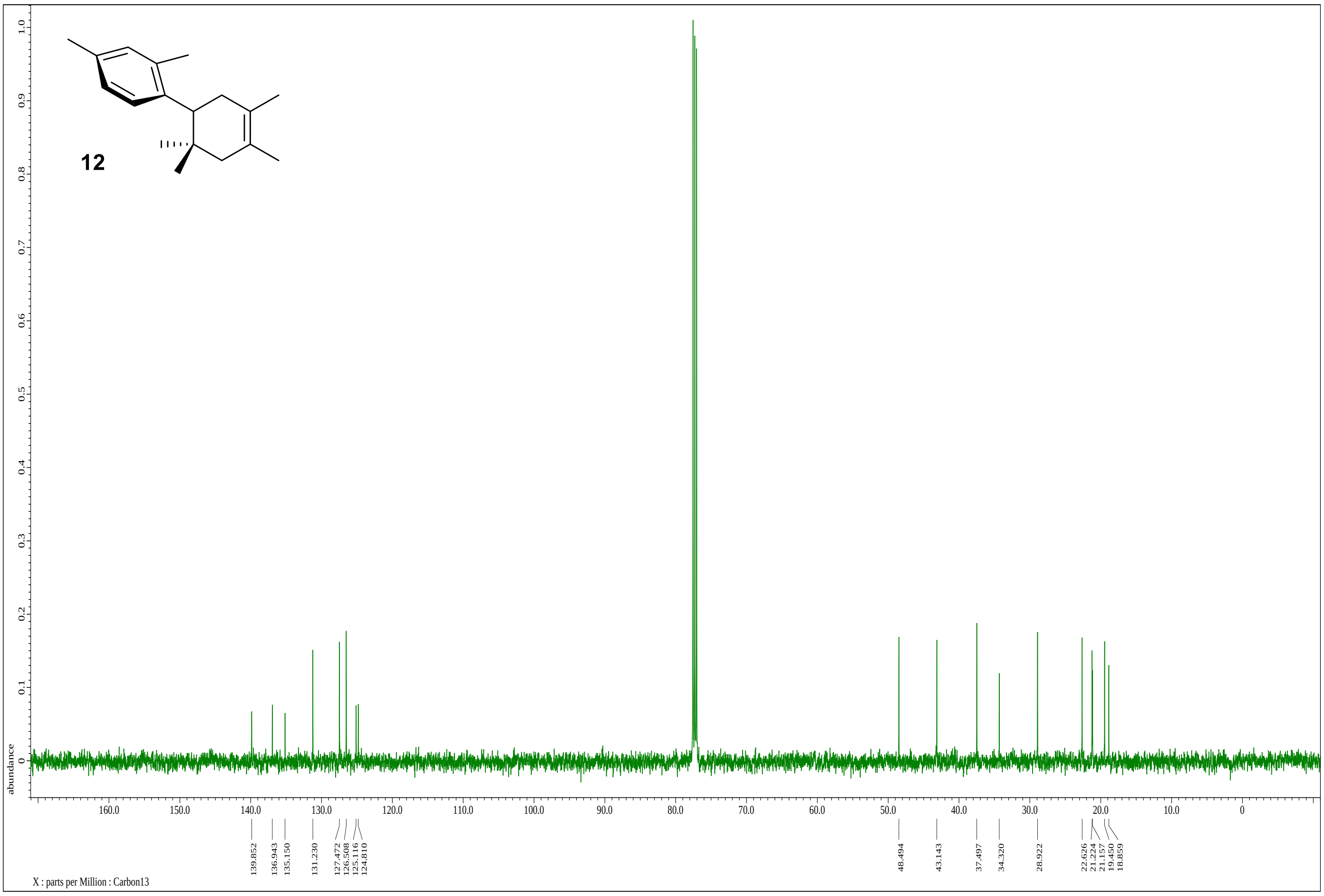
12
abundance
X : parts per Million : Carbon13
139.852
136.943
135.150
131.230
127.472
126.508
125.116
124.810
48.494
43.143
37.497
34.320
28.922
22.626
21.224
21.157
19.450
18.859

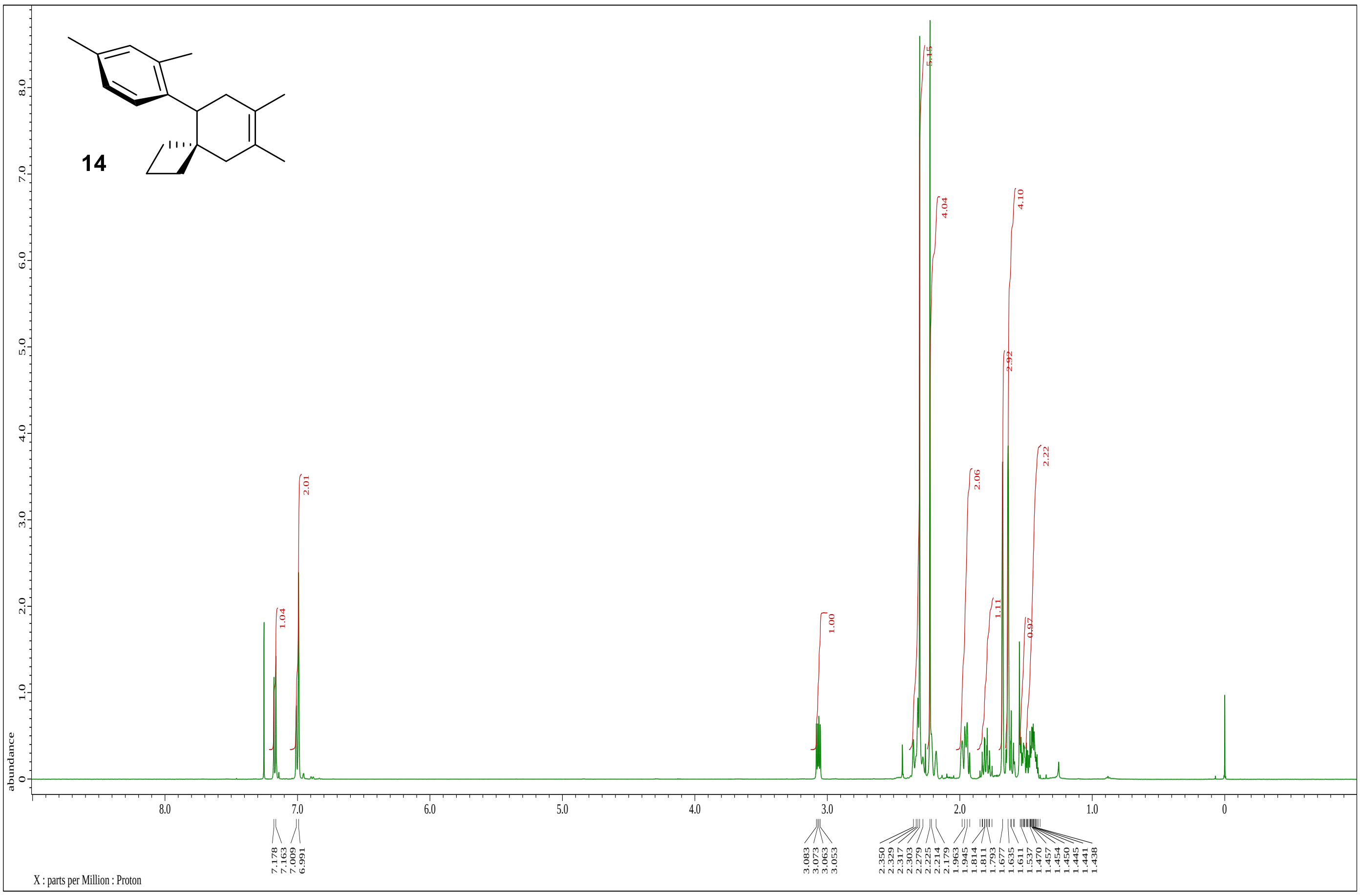
14
abundance
X : parts per Million : Proton
7.178
7.163
7.009
6.991
3.083
3.073
3.063
3.053
2.350
2.329
2.317
2.303
2.279
2.225
2.214
2.179
1.963
1.945
1.814
1.811
1.793
1.677
1.635
1.611
1.537
1.470
1.457
1.454
1.450
1.445
1.441
1.438
1.04
2.01
1.00
5.15
4.04
2.06
1.11
2.92
0.97
4.10
2.22

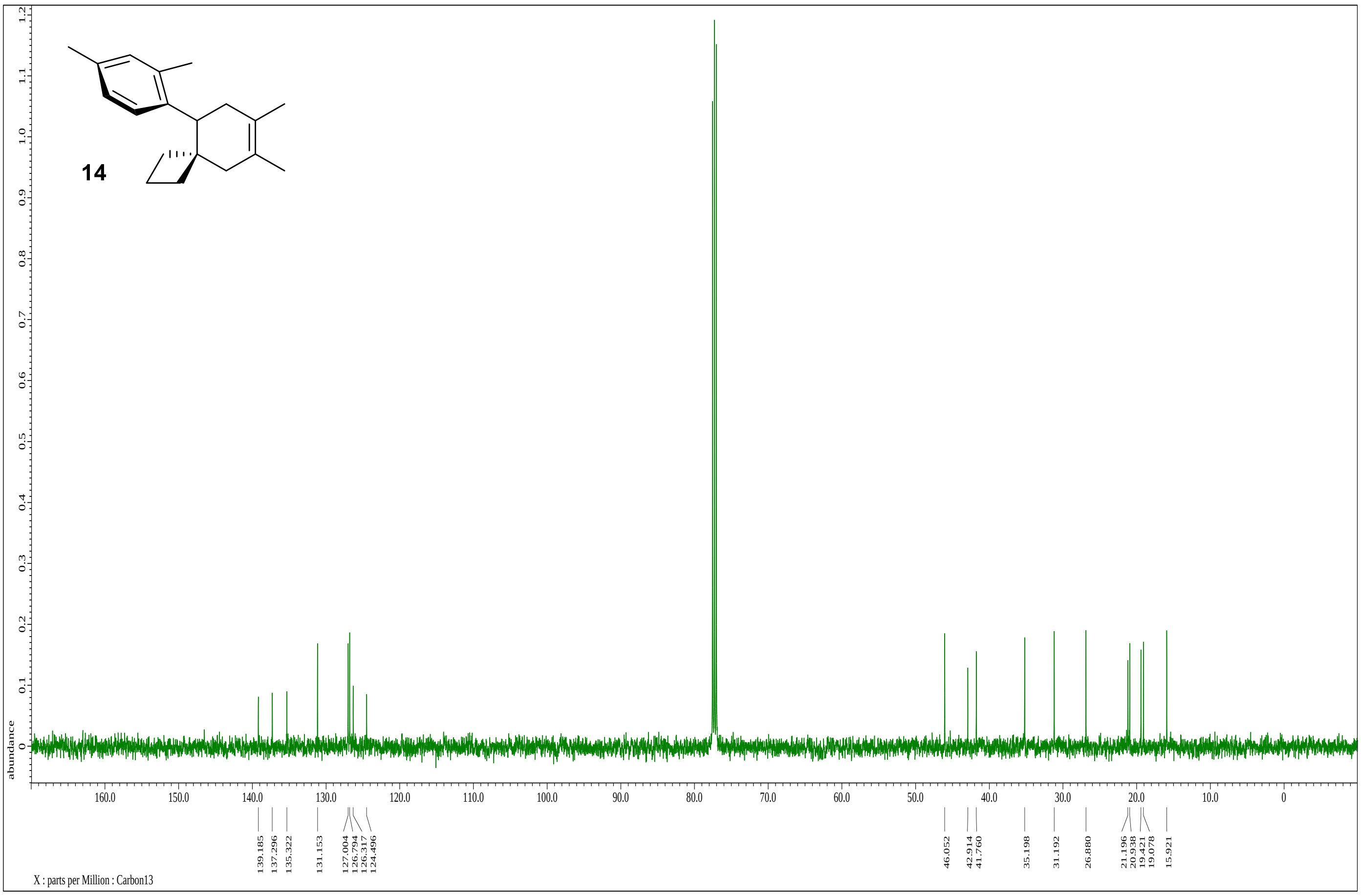
14
abundance
X : parts per Million : Carbon13
139.185
137.296
135.322
131.153
127.004
126.794
126.317
124.496
46.052
42.914
41.760
35.198
31.192
26.880
21.196
20.938
19.421
19.078
15.921

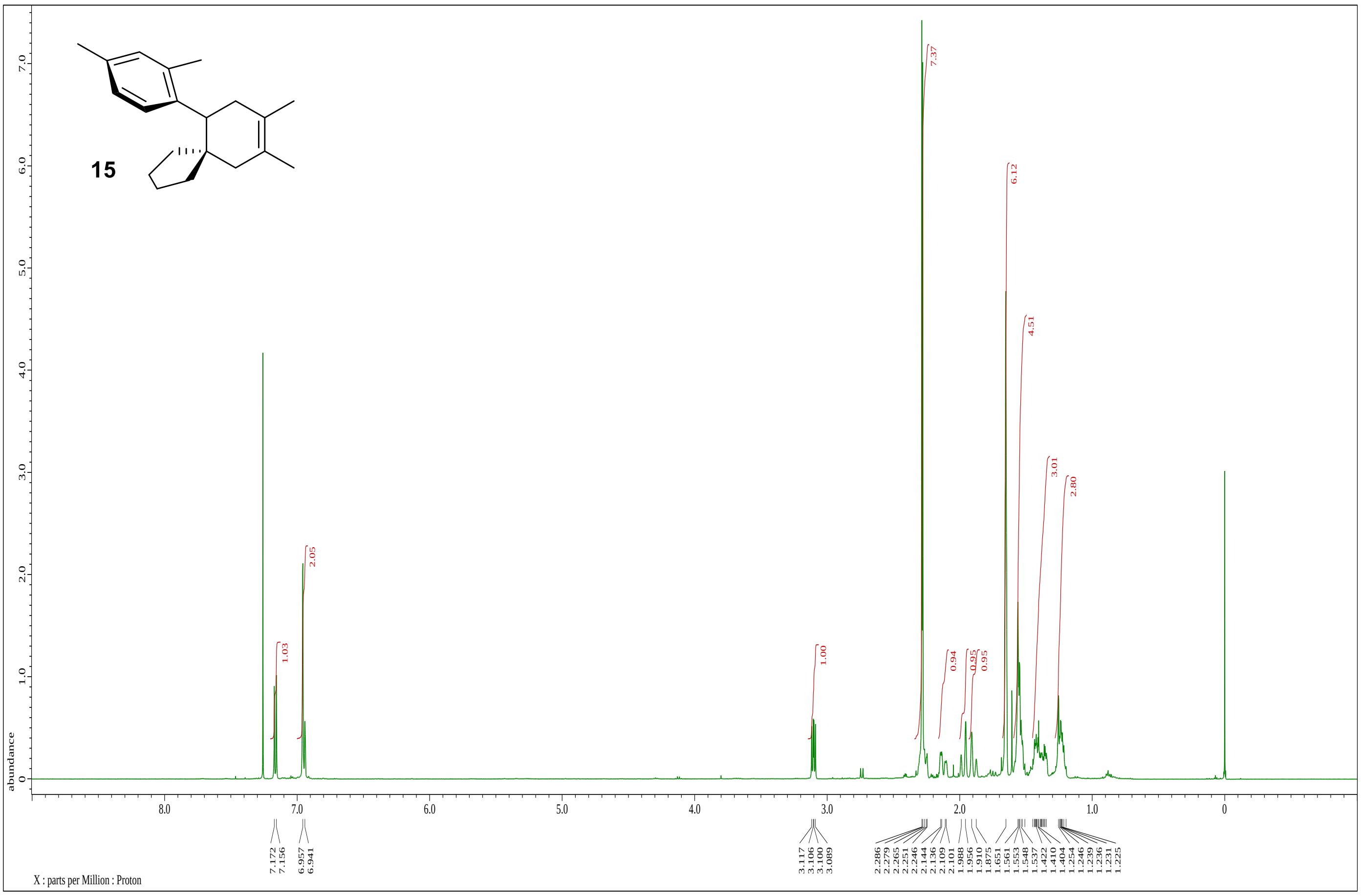
15
abundance
X : parts per Million : Proton
7.37
6.12
4.51
3.01
2.80
2.05
1.03
1.00
0.94
0.95
0.95
7.172
7.156
6.957
6.941
3.117
3.106
3.100
3.089
2.286
2.279
2.265
2.251
2.246
2.144
2.136
2.109
2.101
1.988
1.956
1.910
1.875
1.651
1.561
1.553
1.548
1.537
1.422
1.410
1.404
1.254
1.246
1.239
1.236
1.231
1.225

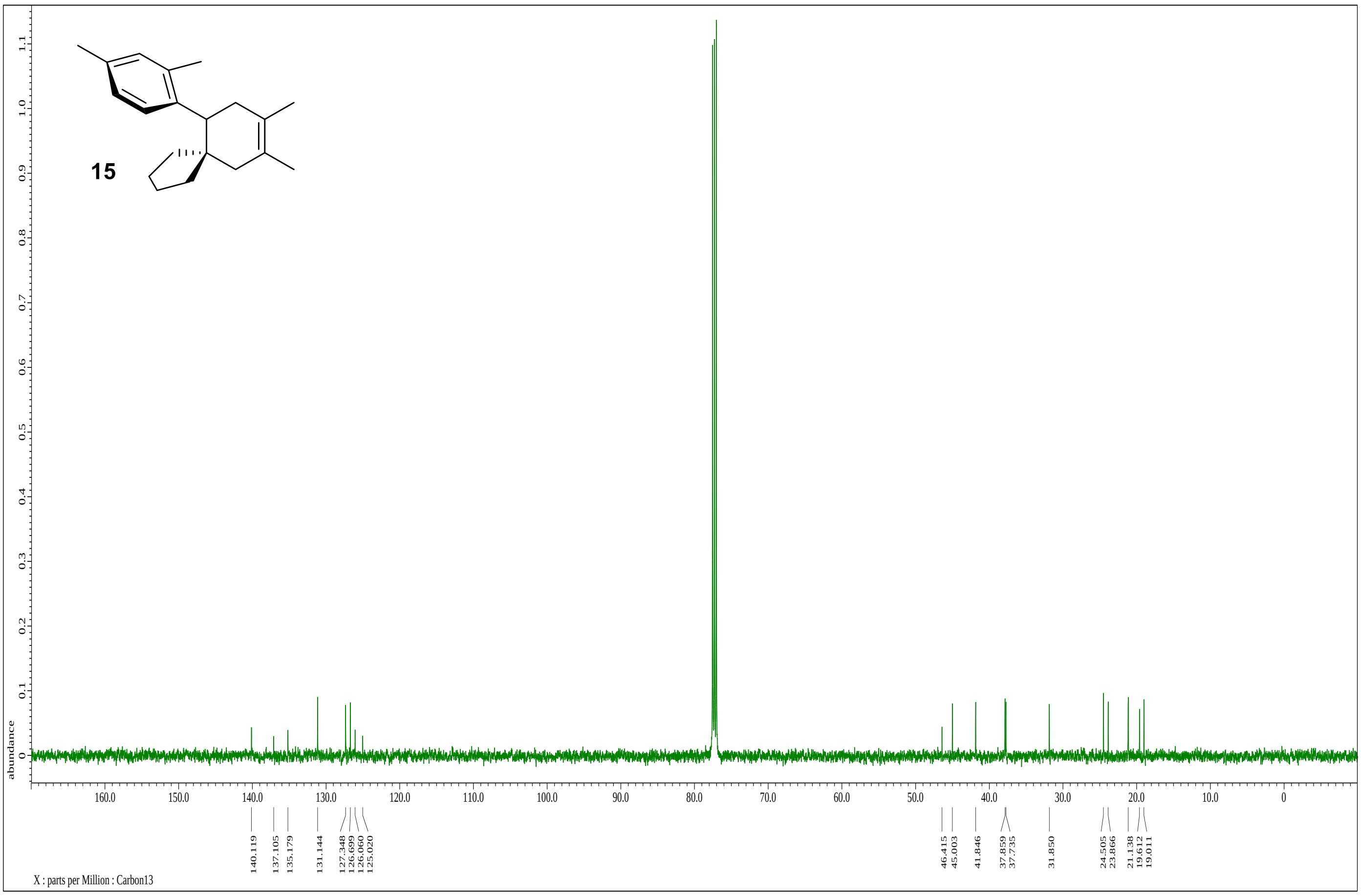
15
abundance
X : parts per Million : Carbon13
140.119
137.105
135.179
131.144
127.348
126.699
126.060
125.020
46.415
45.003
41.846
37.859
37.735
31.850
24.505
23.866
21.138
19.612
19.011

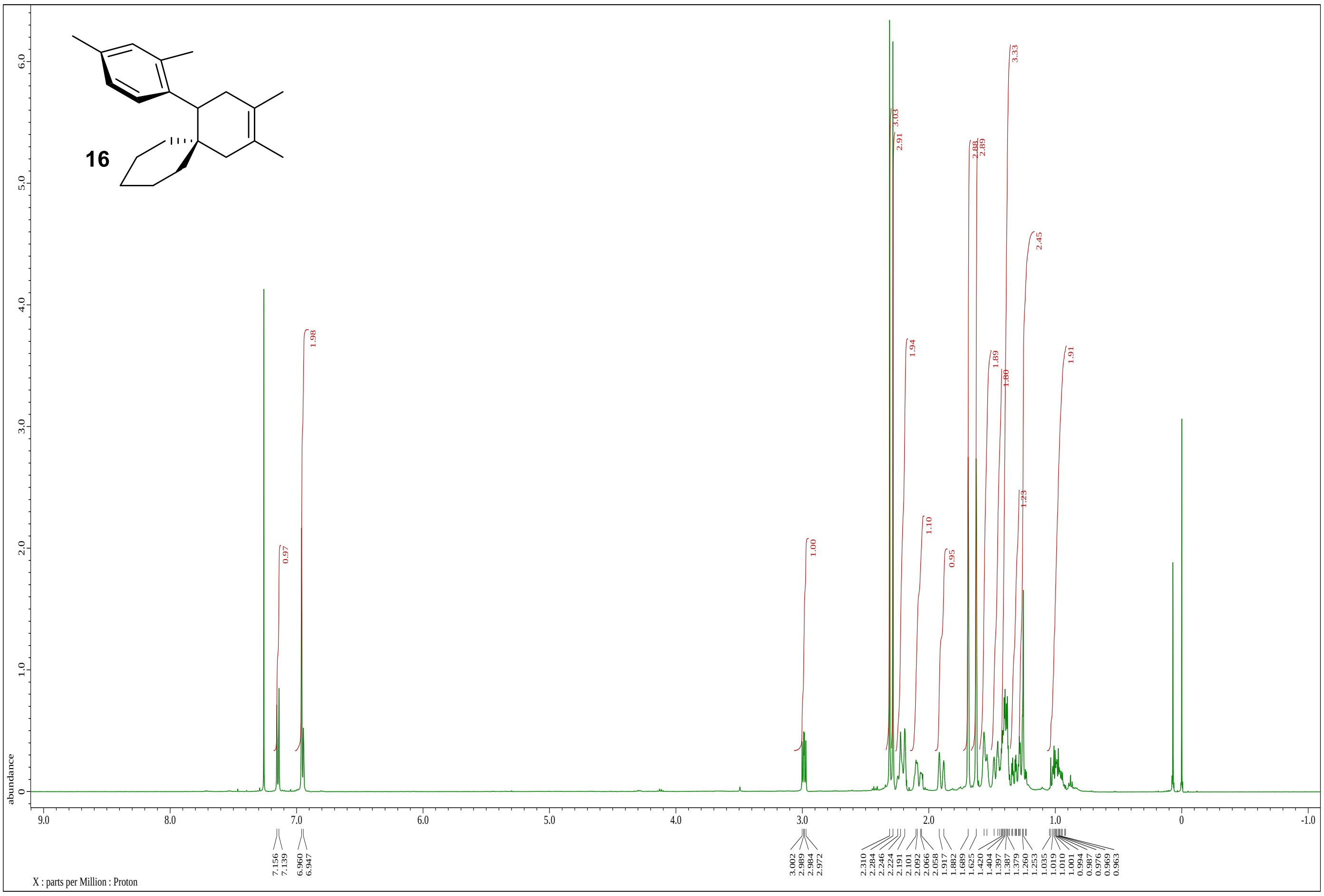
16
abundance
X : parts per Million : Proton
0.97
1.98
1.00
3.03
2.91
1.94
1.10
0.95
2.88
2.89
1.89
1.80
3.33
1.23
2.45
1.91
7.156
7.139
6.960
6.947
3.002
2.989
2.984
2.972
2.310
2.284
2.246
2.224
2.191
2.101
2.092
2.066
2.058
1.917
1.882
1.689
1.625
1.420
1.404
1.397
1.387
1.379
1.260
1.253
1.035
1.019
1.010
1.001
0.994
0.987
0.976
0.969
0.963

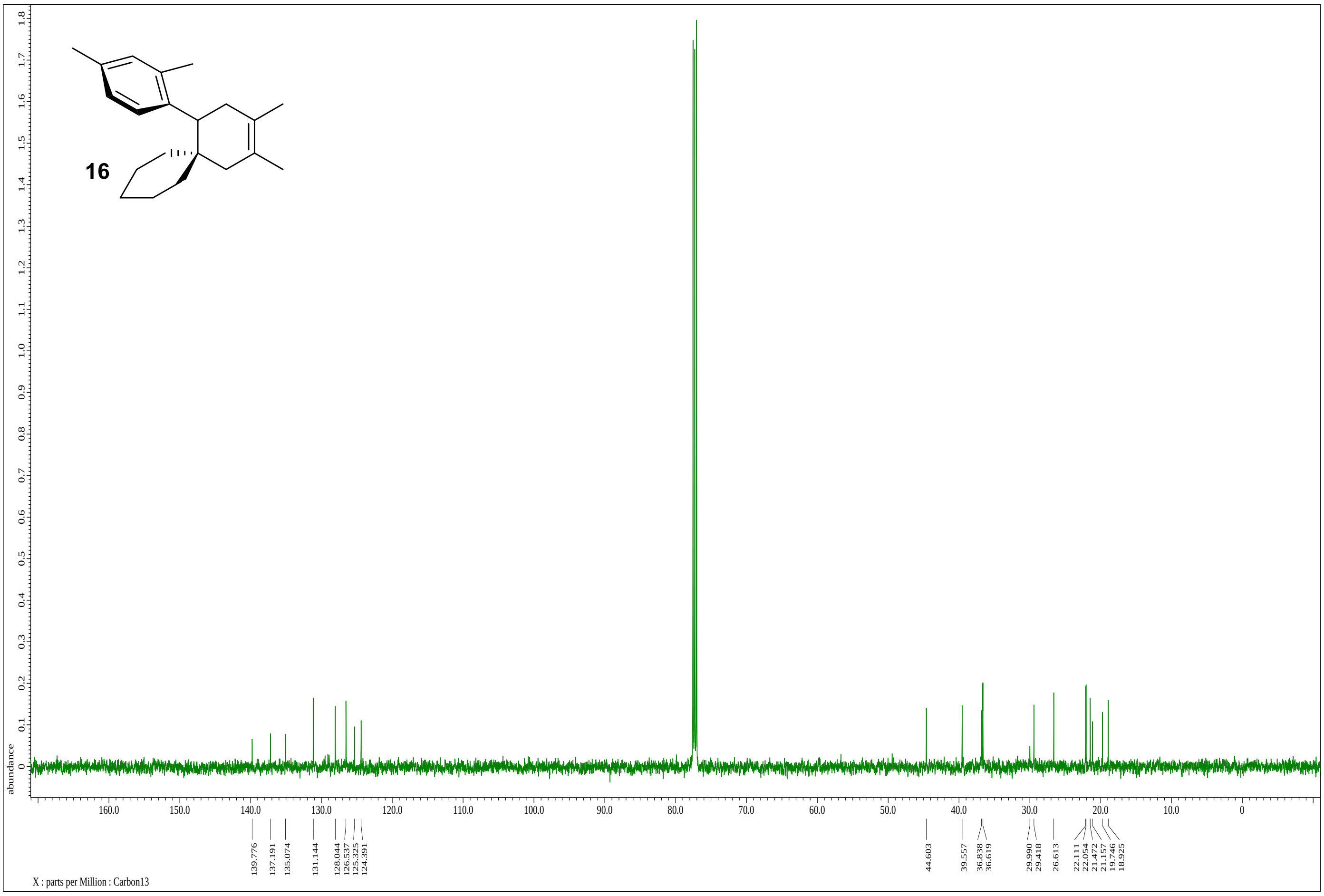
16
abundance
X : parts per Million : Carbon13
139.776
137.191
135.074
131.144
128.044
126.537
125.325
124.391
44.603
39.557
36.838
36.619
29.990
29.418
26.613
22.111
22.054
21.472
21.157
19.746
18.925

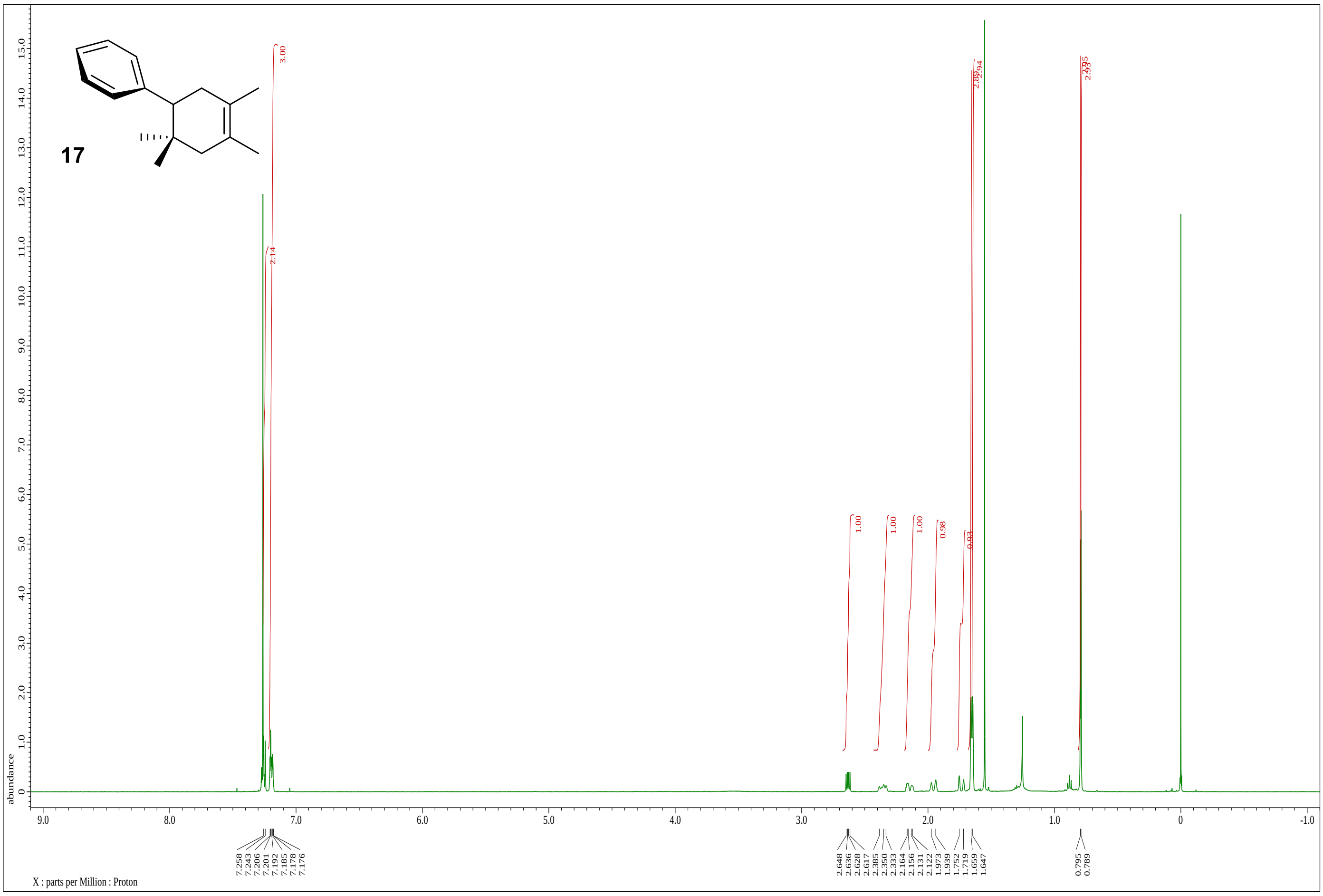
17
X : parts per Million : Proton
abundance
7.258
7.243
7.206
7.201
7.192
7.185
7.178
7.176
2.648
2.636
2.628
2.617
2.385
2.350
2.333
2.164
2.156
2.131
2.122
1.973
1.939
1.752
1.719
1.659
1.647
0.795
0.789

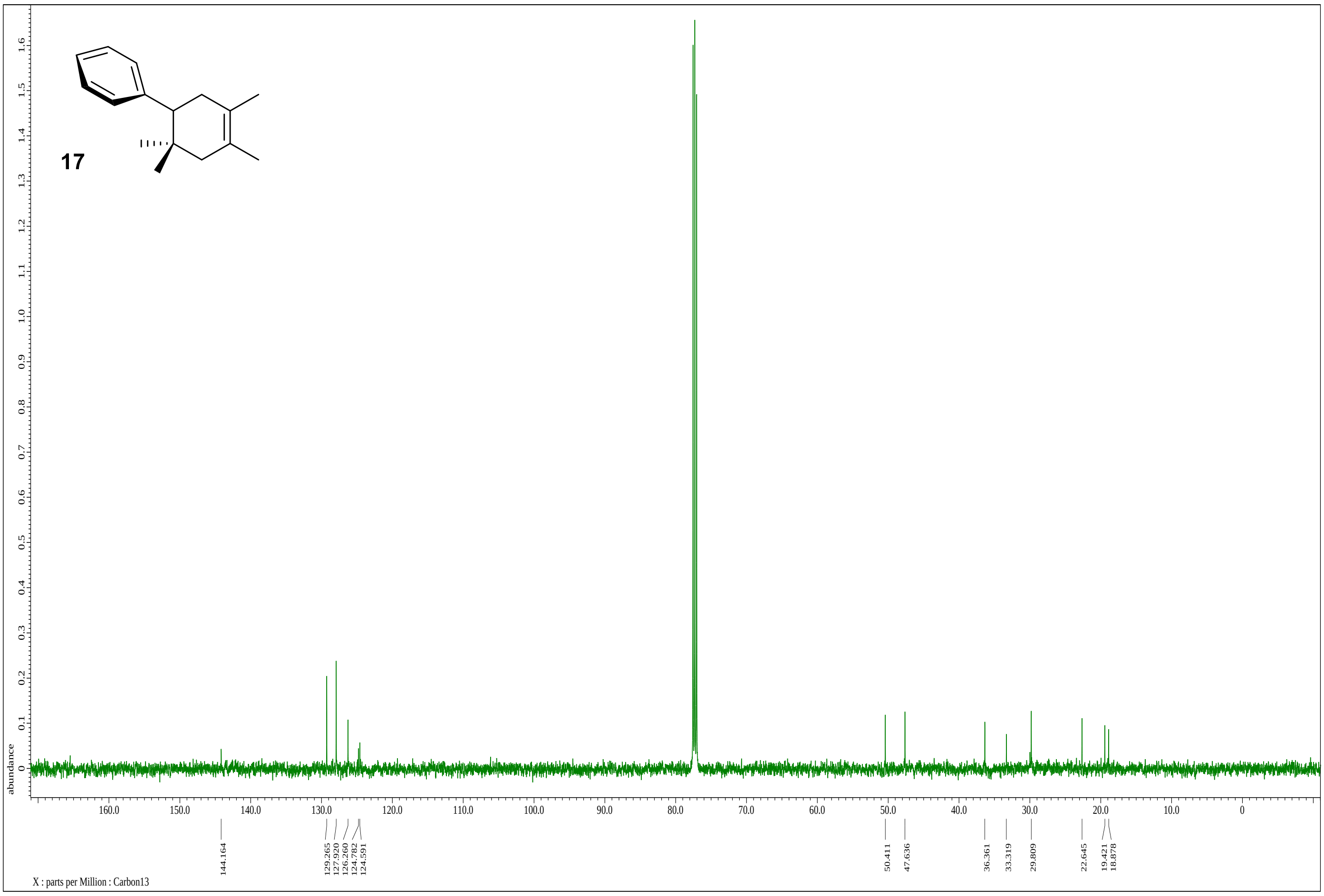
17
X : parts per Million : Carbon13
abundance
144.164
129.265
127.920
126.260
124.782
124.591
50.411
47.636
36.361
33.319
29.809
22.645
19.421
18.878

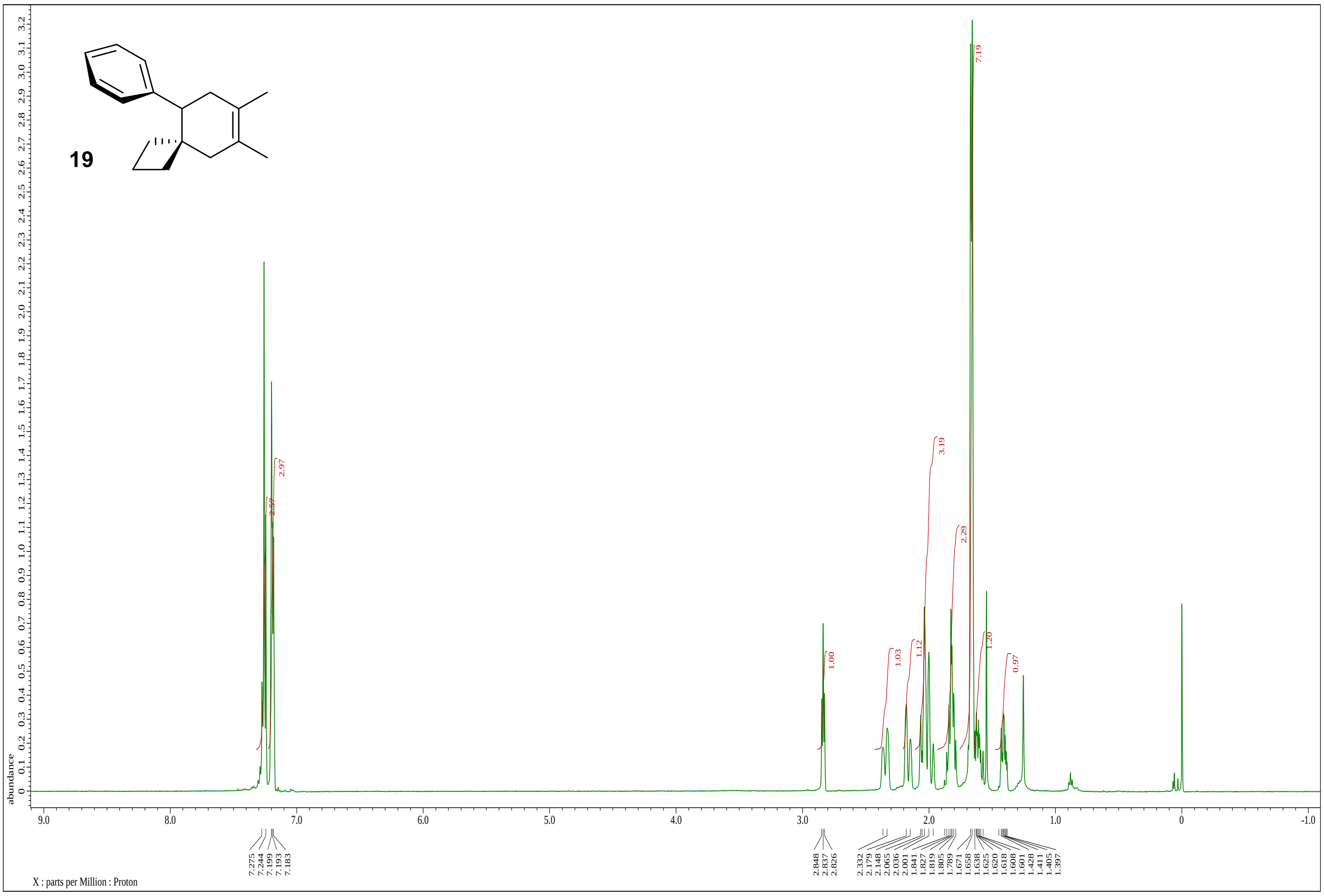

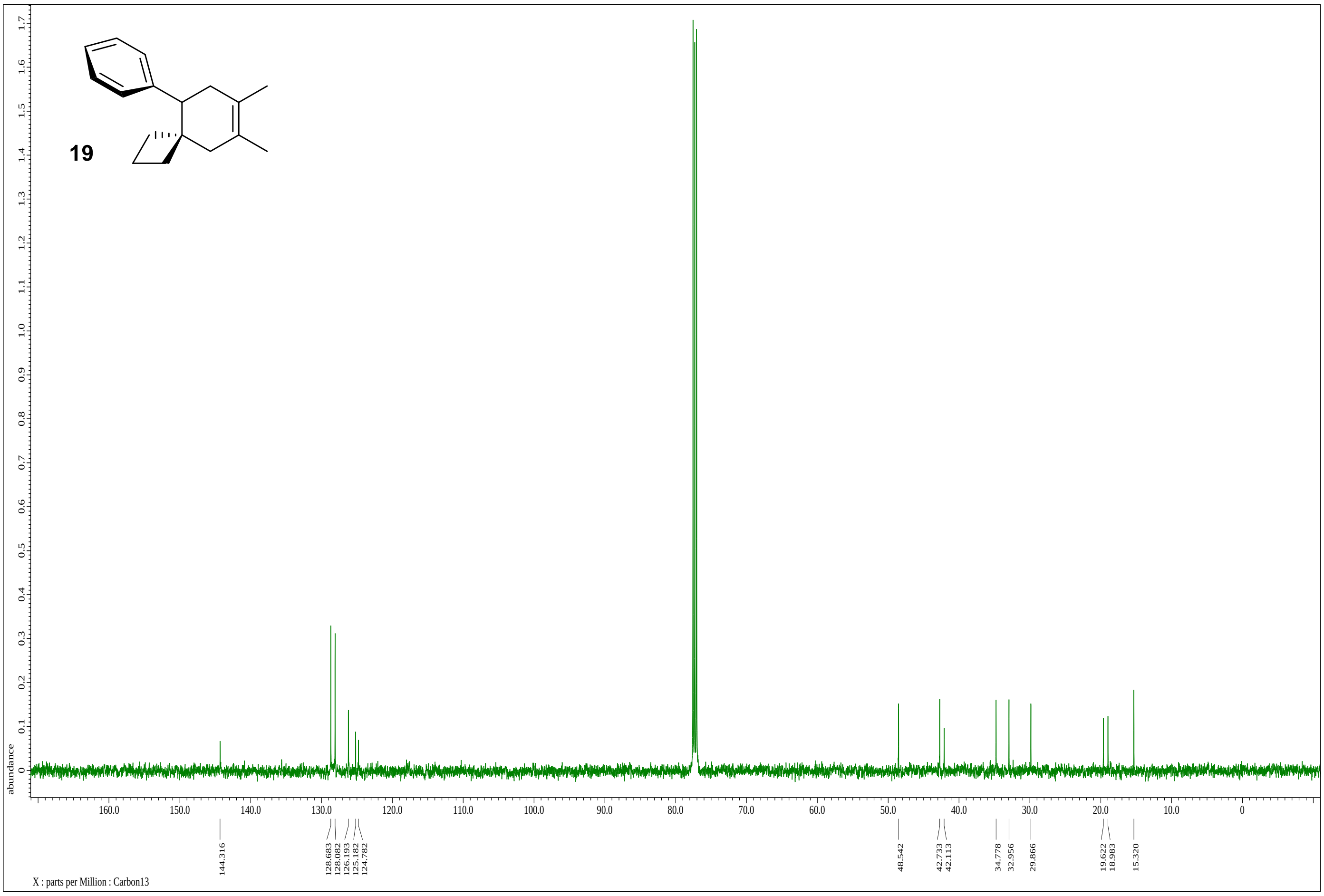

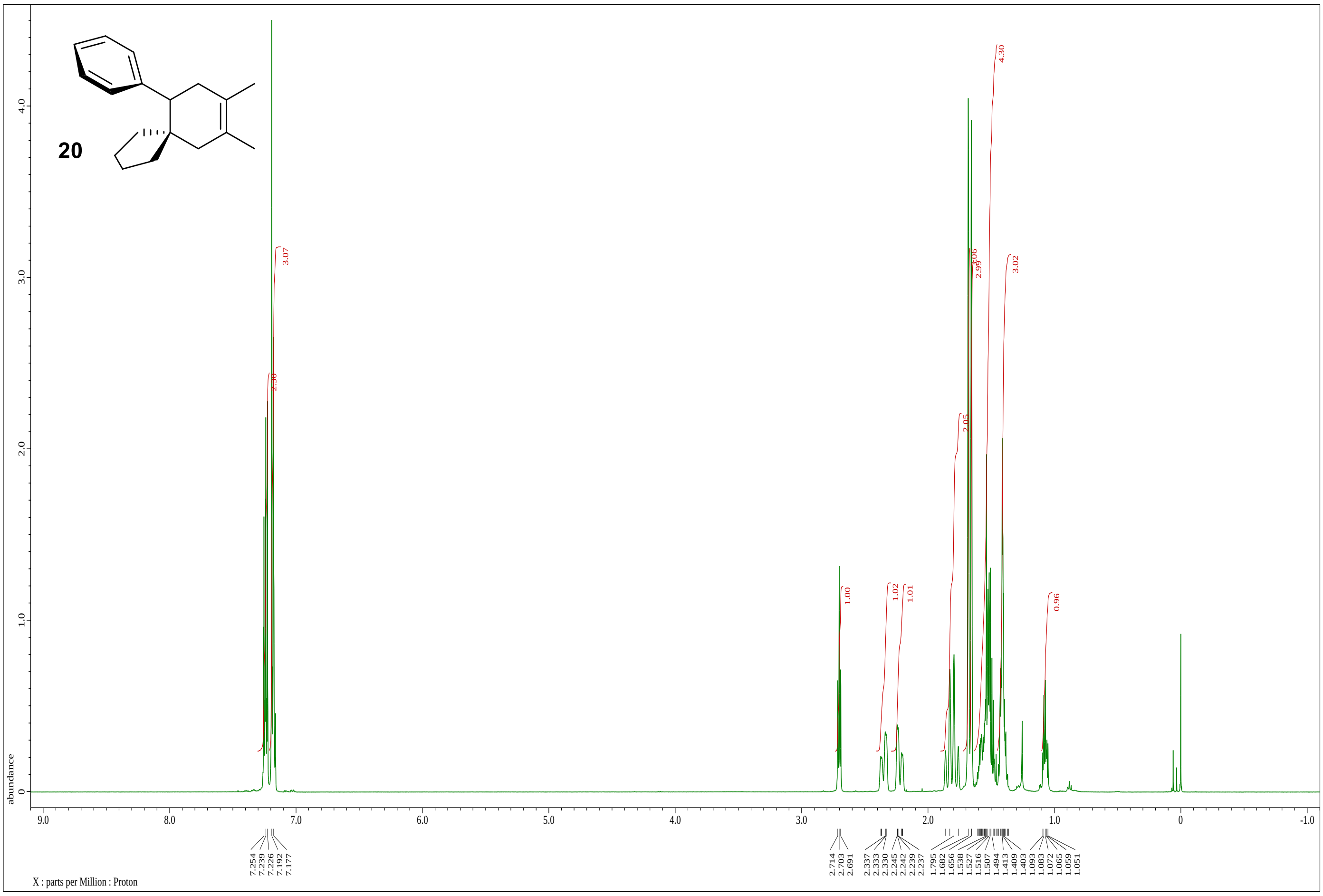
20
abundance
X : parts per Million : Proton
3.07
2.30
1.00
1.02
1.01
2.05
3.06
2.99
3.02
4.30
0.96
7.254
7.239
7.226
7.192
7.177
2.714
2.703
2.691
2.337
2.333
2.330
2.245
2.242
2.239
2.237
1.795
1.682
1.656
1.538
1.527
1.516
1.507
1.494
1.413
1.409
1.403
1.093
1.083
1.072
1.065
1.059
1.051

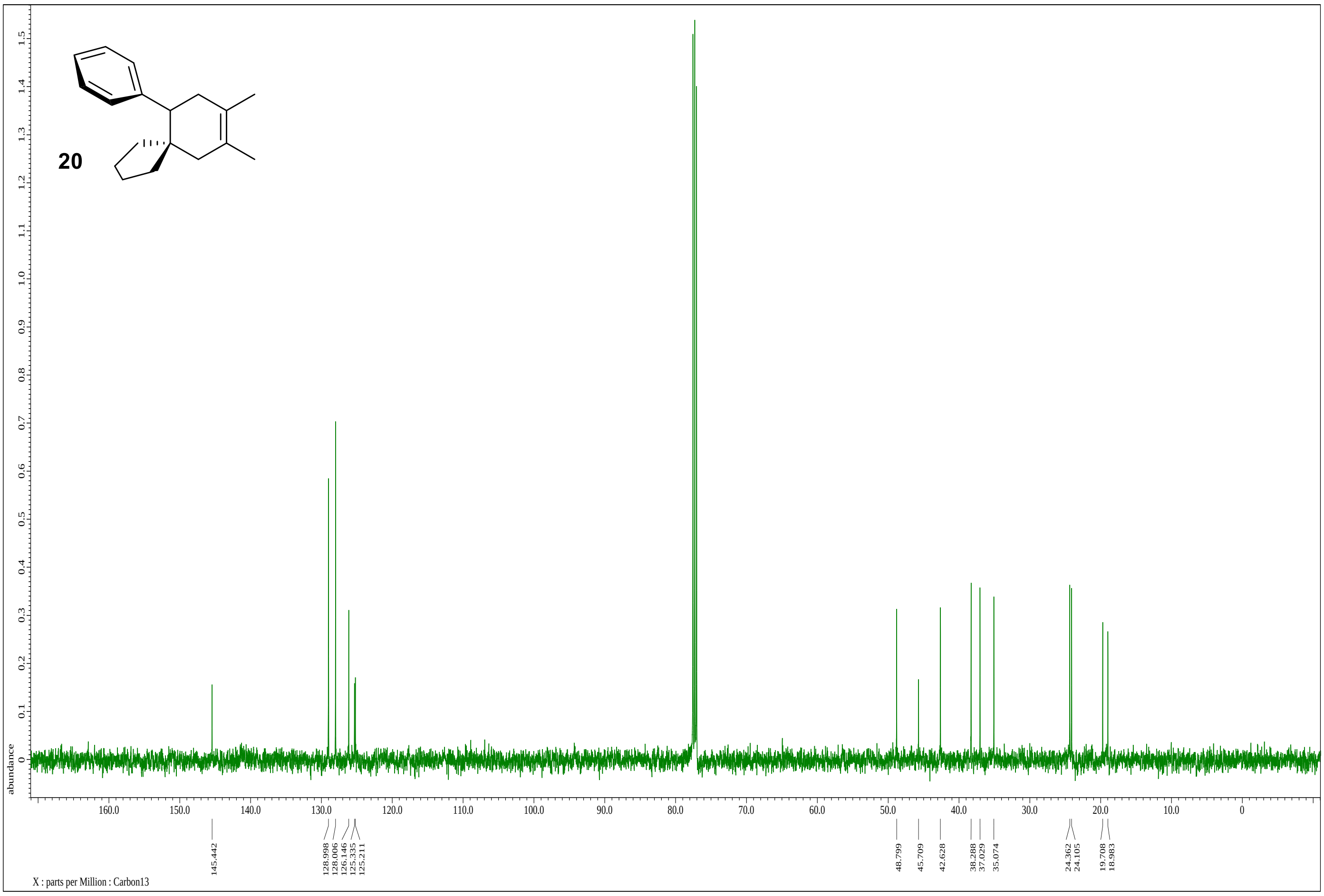
20
abundance
X : parts per Million : Carbon13
145.442
128.998
128.006
126.146
125.335
125.211
48.799
45.709
42.628
38.288
37.029
35.074
24.362
24.105
19.708
18.983

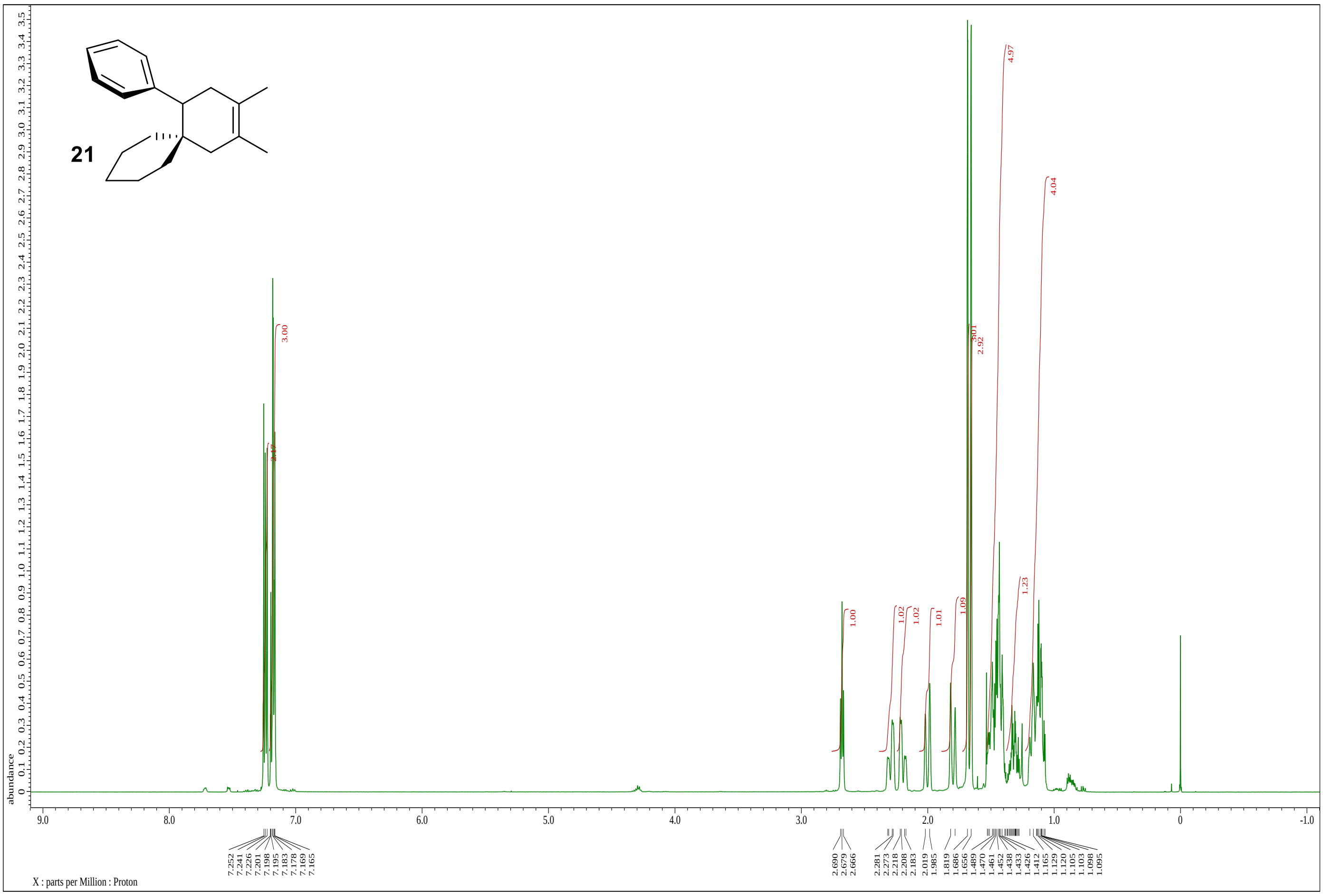
21
abundance
X : parts per Million : Proton
7.252
7.241
7.226
7.201
7.198
7.195
7.183
7.178
7.169
7.165
2.690
2.679
2.666
2.281
2.273
2.218
2.208
2.183
2.019
1.985
1.819
1.686
1.656
1.489
1.470
1.461
1.452
1.438
1.433
1.426
1.412
1.165
1.129
1.120
1.105
1.103
1.098
1.095
3.00
1.00
1.02
1.02
1.01
1.09
3.01
2.92
4.97
1.23
4.04

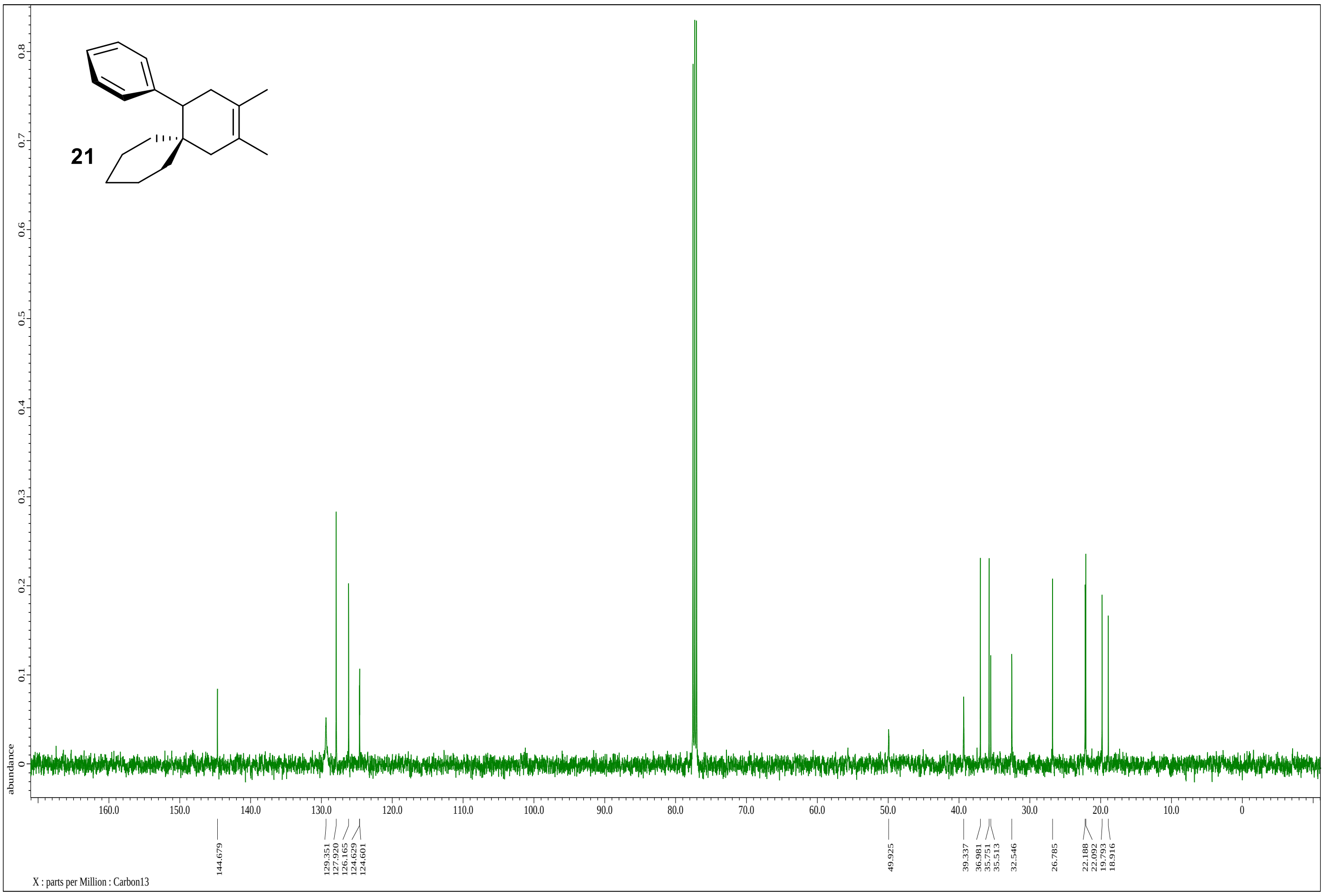
21
abundance
X : parts per Million : Carbon13
144.679
129.351
127.920
126.165
124.629
124.601
49.925
39.337
36.981
35.751
35.513
32.546
26.785
22.188
22.092
19.793
18.916

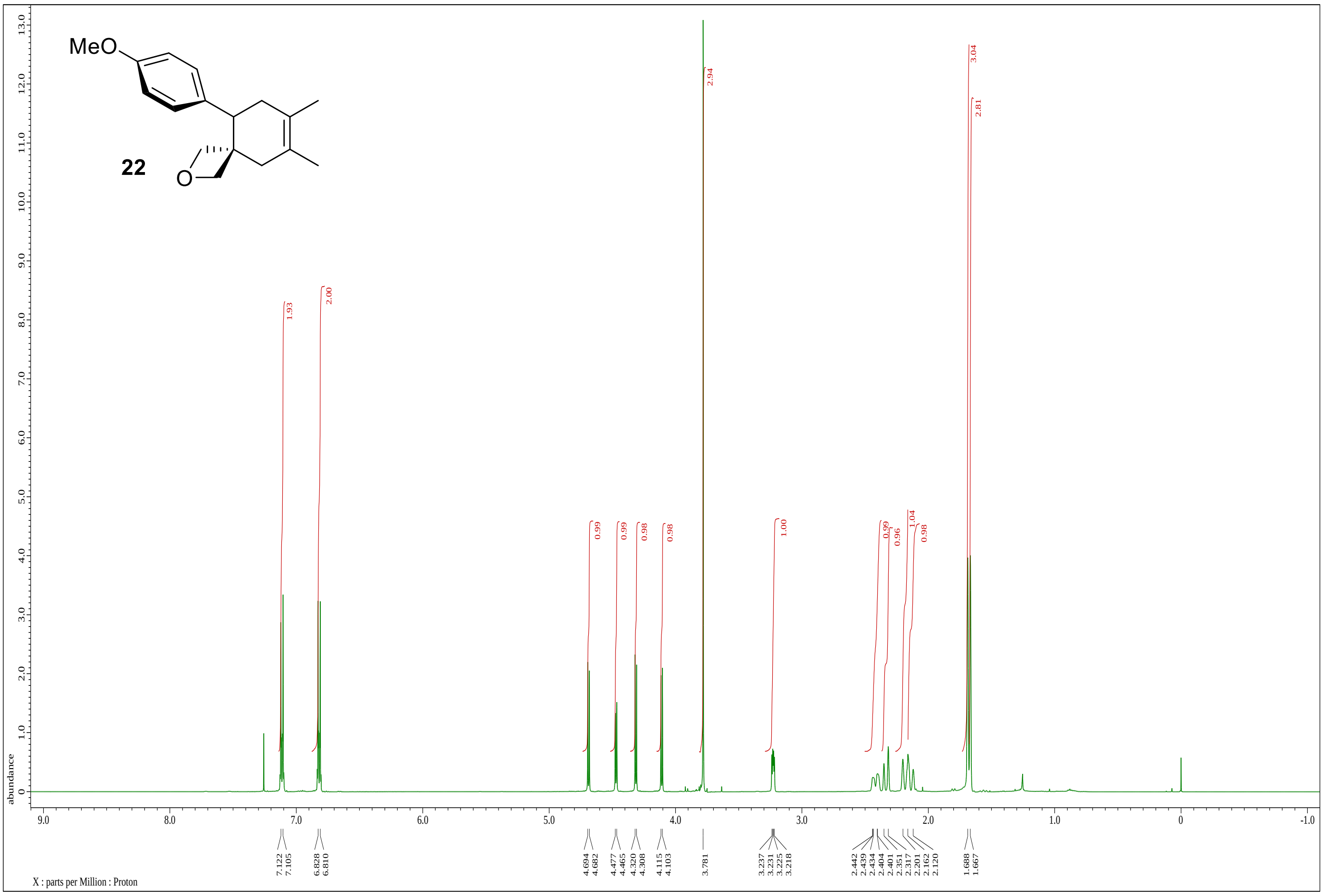
MeO
22
O
abundance
7.122
7.105
6.828
6.810
4.694
4.682
4.477
4.465
4.320
4.308
4.115
4.103
3.781
3.237
3.231
3.225
3.218
2.442
2.439
2.434
2.404
2.401
2.351
2.317
2.201
2.162
2.120
1.688
1.667
1.93
2.00
0.99
0.99
0.98
0.98
2.94
1.00
0.99
0.96
1.04
0.98
3.04
2.81
X : parts per Million : Proton

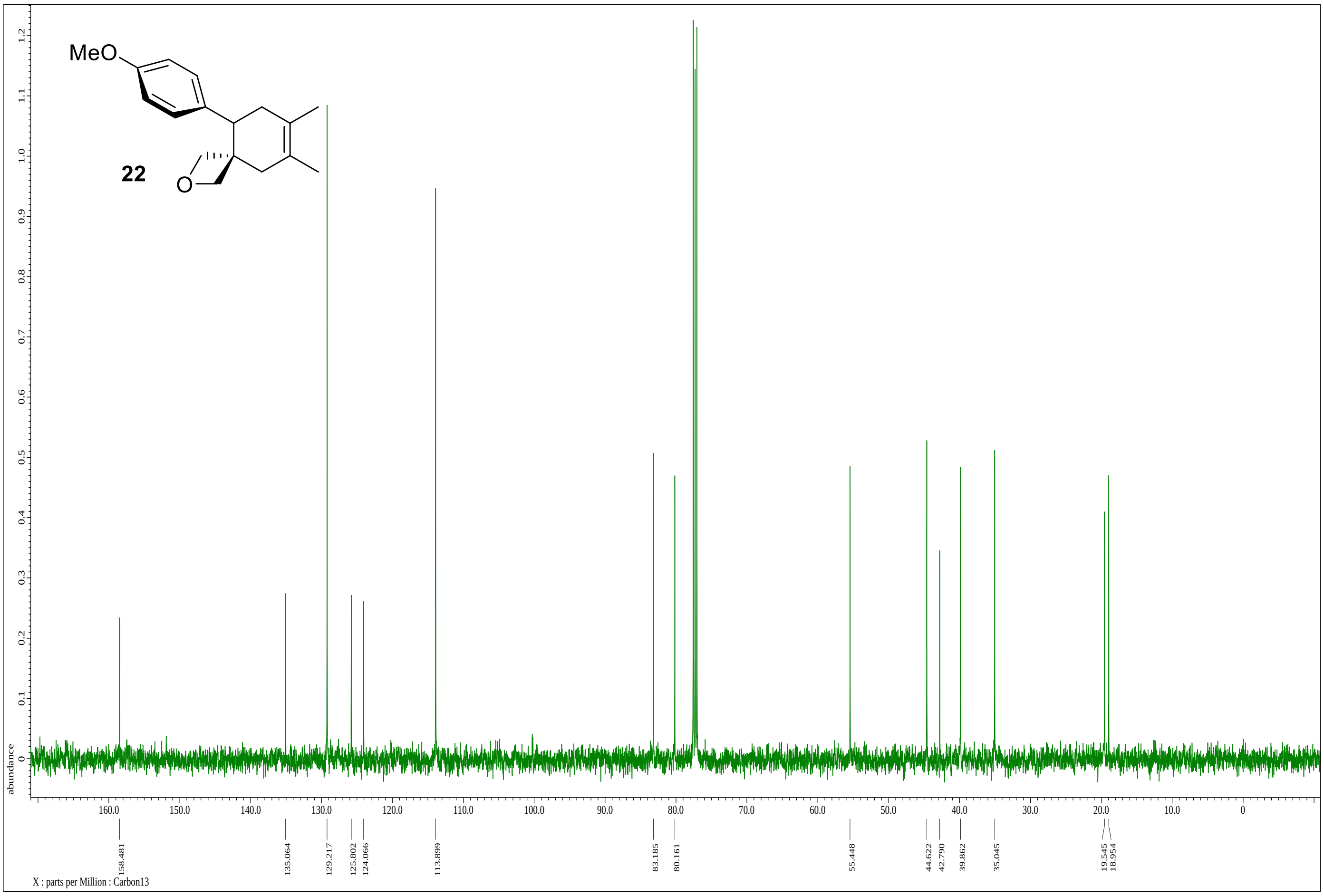
MeO
22
O
abundance
158.481
135.064
129.217
125.802
124.066
113.899
83.185
80.161
55.448
44.622
42.790
39.862
35.045
19.545
18.954
X : parts per Million : Carbon13

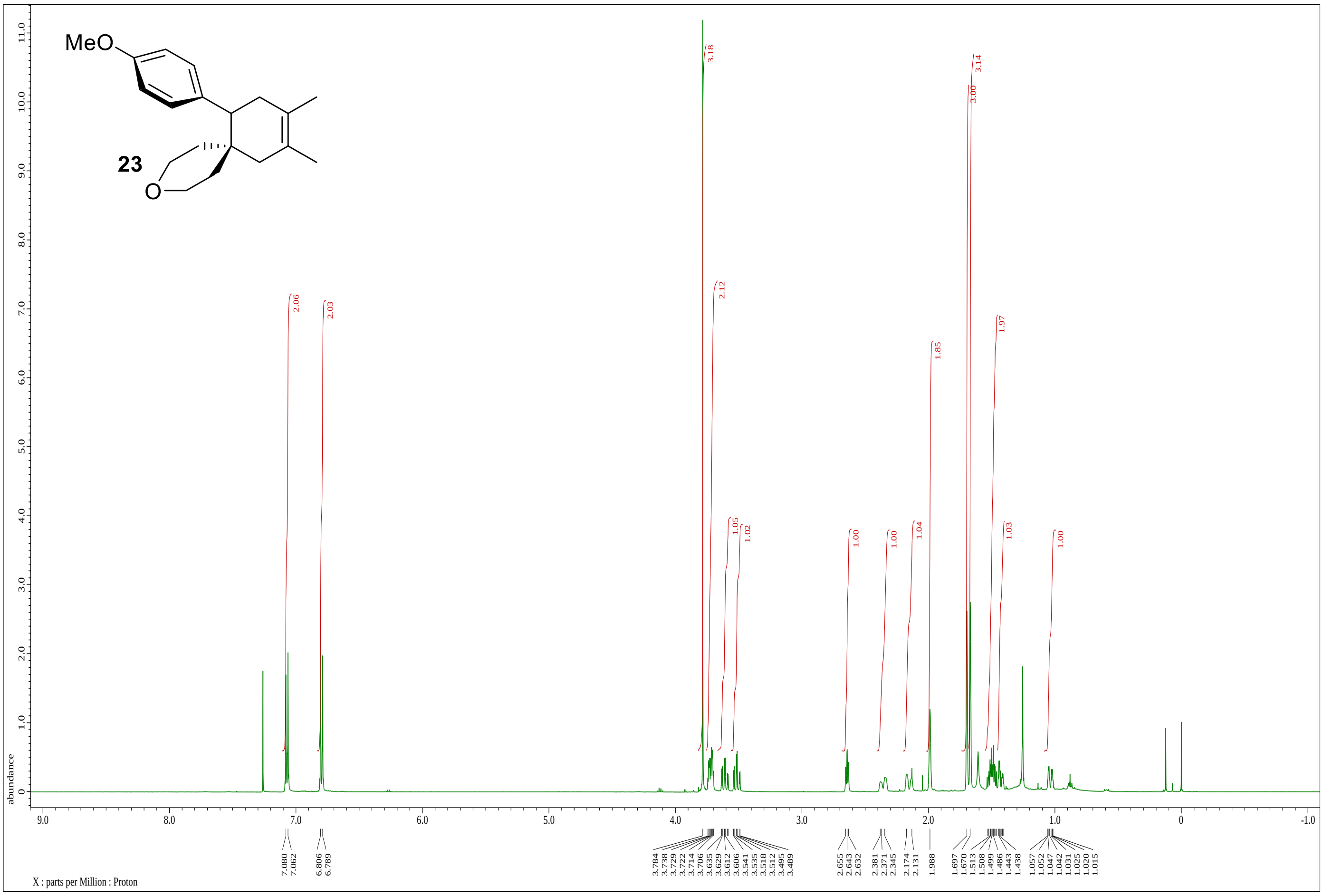
MeO
23
O
3.18
2.06
2.03
2.12
1.05
1.02
1.00
1.00
1.04
1.85
3.00
3.14
1.97
1.03
1.00
7.080
7.062
6.806
6.789
3.784
3.738
3.729
3.722
3.714
3.706
3.635
3.629
3.612
3.606
3.541
3.535
3.518
3.512
3.495
3.489
2.655
2.643
2.632
2.381
2.371
2.345
2.174
2.131
1.988
1.697
1.670
1.513
1.508
1.499
1.486
1.443
1.438
1.057
1.052
1.047
1.042
1.031
1.025
1.020
1.015
abundance
X : parts per Million : Proton

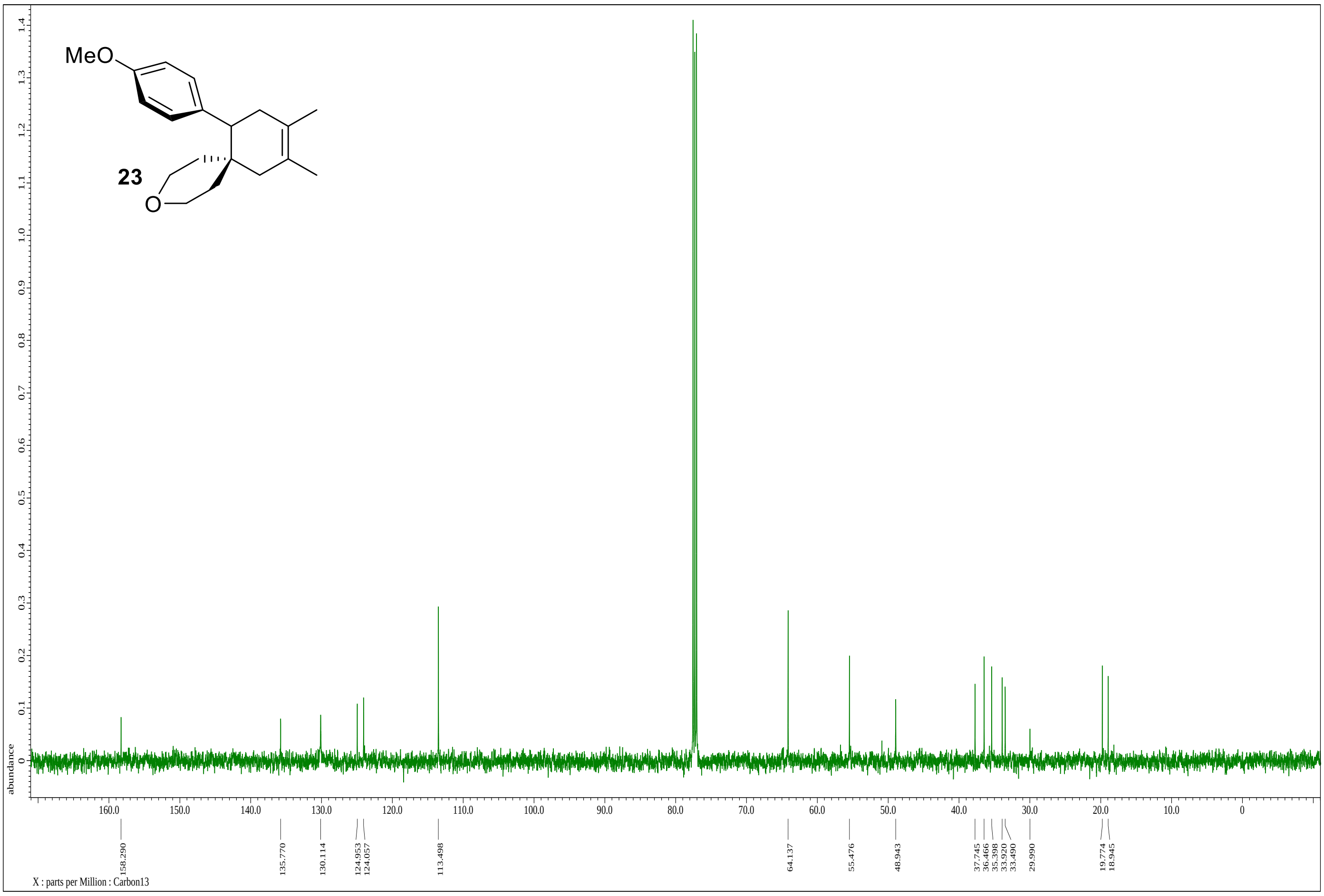
MeO
23
O
158.290
135.770
130.114
124.953
124.057
113.498
64.137
55.476
48.943
37.745
36.466
35.398
33.920
33.490
29.990
19.774
18.945
abundance
X : parts per Million : Carbon13

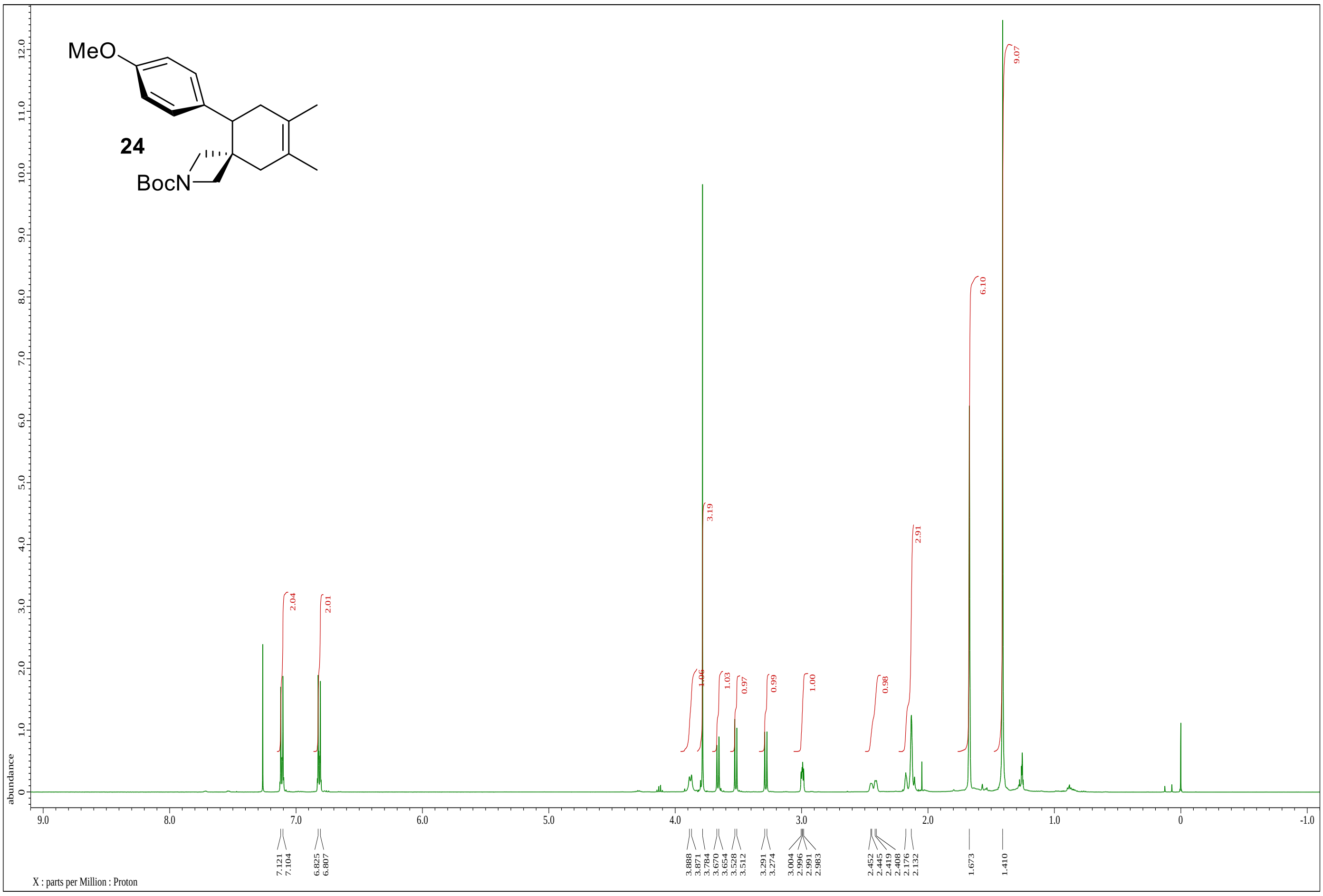

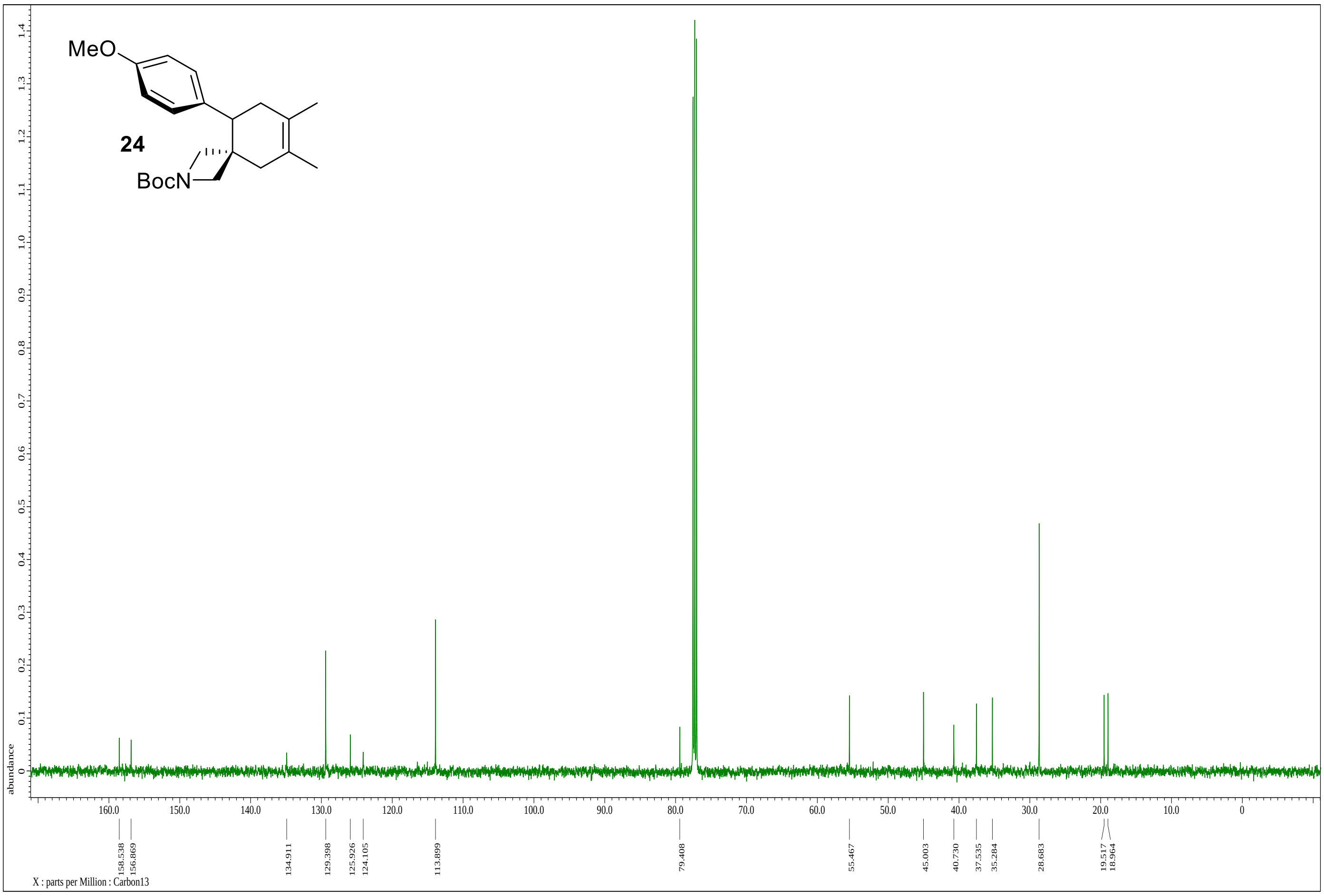

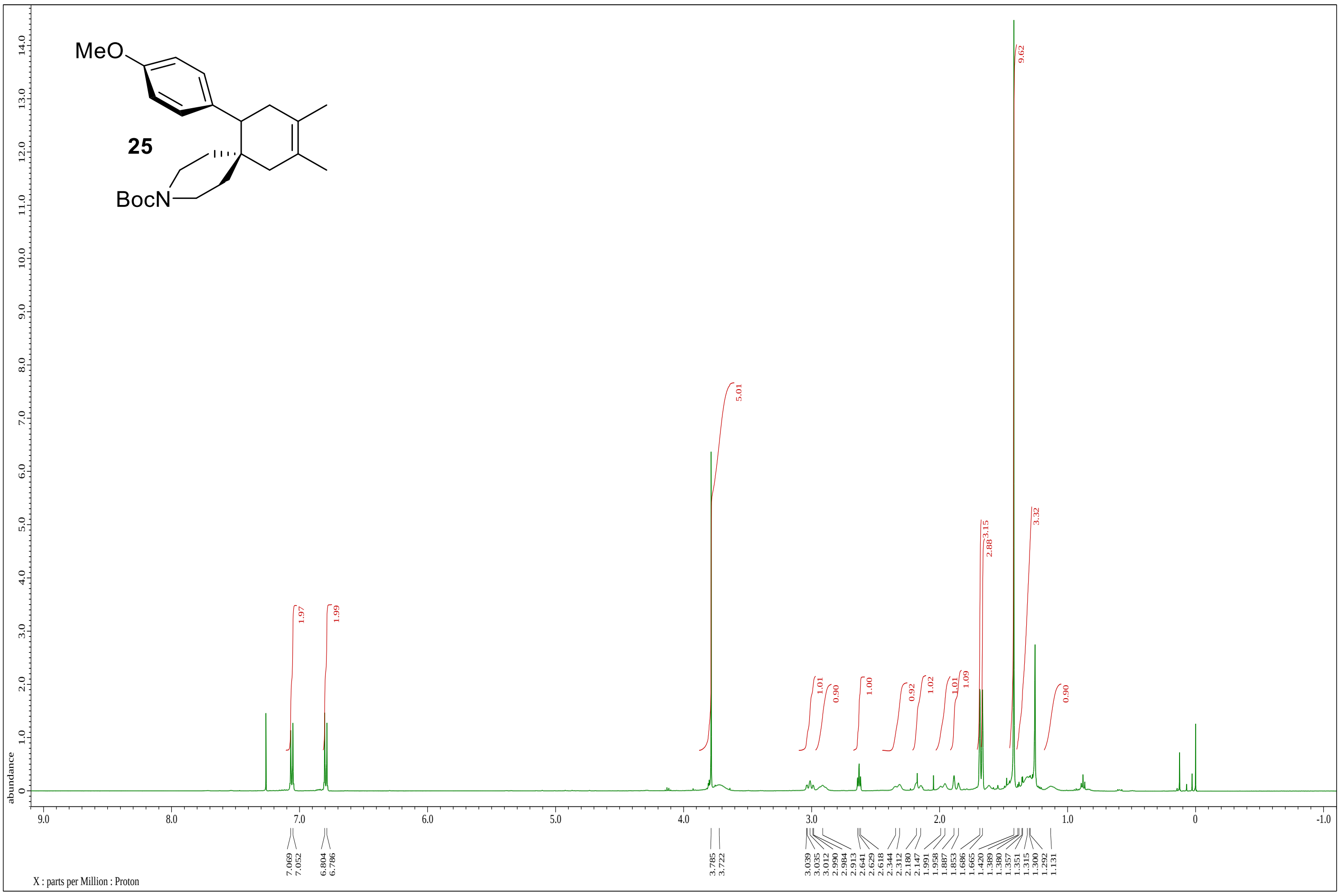
MeO
25
BocN
X : parts per Million : Proton
abundance
7.069
7.052
6.804
6.786
3.785
3.722
3.039
3.035
3.012
2.990
2.984
2.913
2.641
2.629
2.618
2.344
2.312
2.180
2.147
1.991
1.958
1.887
1.853
1.686
1.665
1.420
1.389
1.380
1.357
1.351
1.315
1.300
1.292
1.131

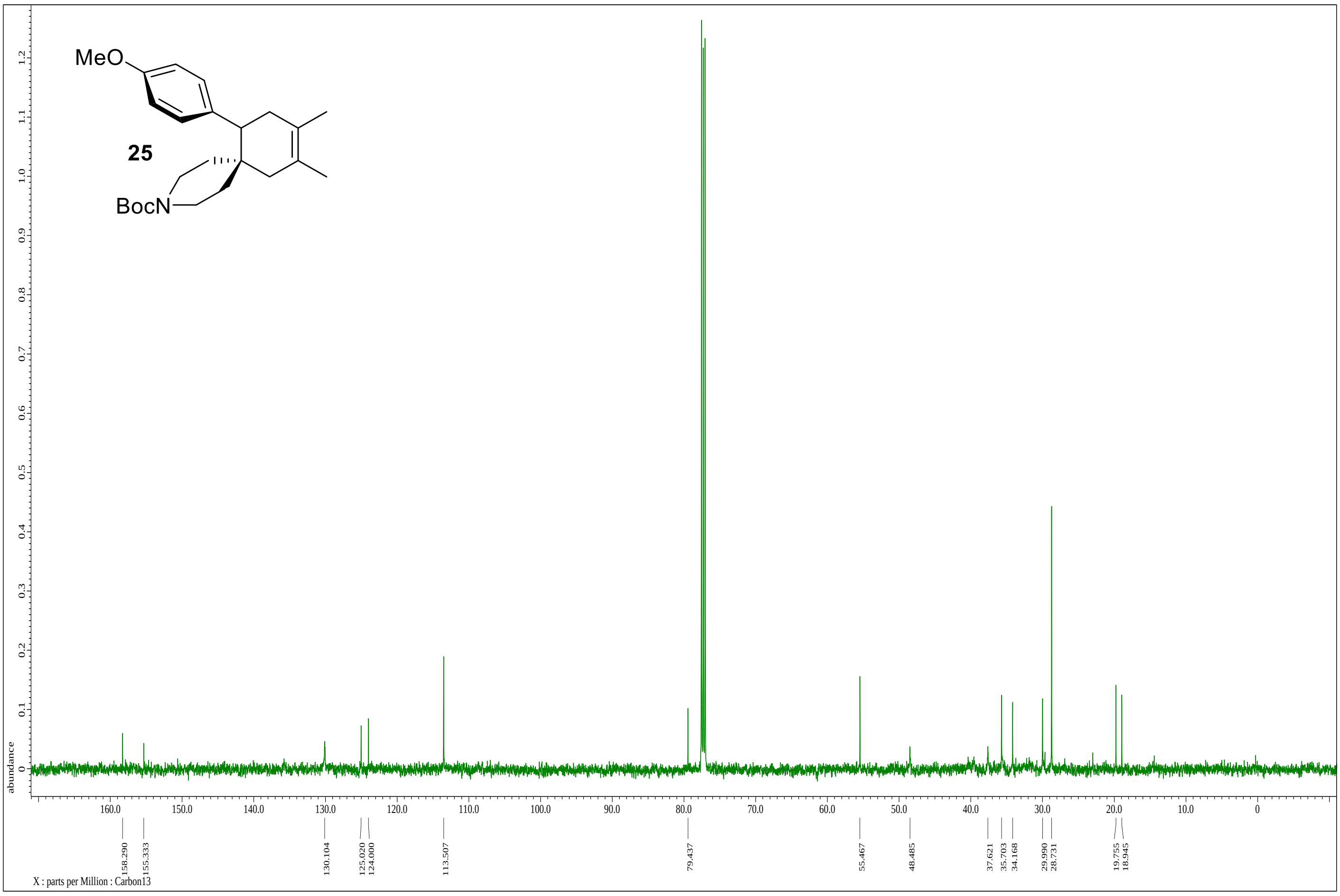
MeO
25
BocN
X : parts per Million : Carbon13
abundance
158.290
155.333
130.104
125.020
124.000
113.507
79.437
55.467
48.485
37.621
35.703
34.168
29.990
28.731
19.755
18.945
